# Ultrasonic-assisted-synthesis of isoindolin-1-one derivatives†

**DOI:** 10.1039/d2ra02720h

**Published:** 2022-06-29

**Authors:** Muhammad Idham Darussalam Mardjan, Muhamad Fadhly Hariadi, Indah Mutiara Putri, Nilna Amalia Musyarrofah, Muflihah Salimah, Priatmoko, Bambang Purwono, Laurent Commeiras

**Affiliations:** Department of Chemistry, Faculty of Mathematics and Natural Sciences, Universitas Gadjah Mada Bulaksumur POS BLS 21 Yogyakarta 55281 Indonesia idham.darussalam@ugm.ac.id; Aix Marseille Univ., CNRS, Centrale Marseille, iSm2 Marseille 13013 France

## Abstract

A small library of 3-hydroxyisoindolin-1-ones has been prepared from 3-alkylidenephtalides under ultrasonic irradiation. This practical synthesis is featured by group tolerance, high efficiency and yields. The reaction can also be performed in multigram scale and be further extended to access other motifs of isoindolin-1-ones in a one-pot fashion.

## Abstract 2

Functionalized isoindolin-1-ones have been prepared in short reaction time and excellent yields from 3-alkylidenephtalides and primary amines under ultrasonic irradiation.

## Graphical abstract

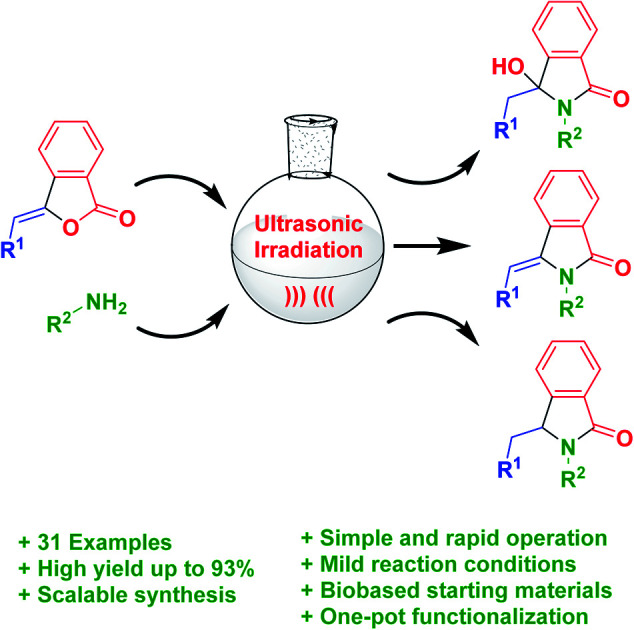

## Introduction

**Fig. 1:**
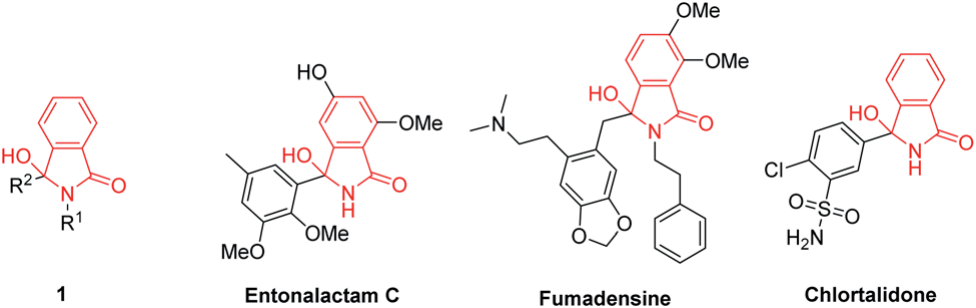
Representatives of bioactive molecules containing 3-hydroxyisoindolin-1-one moiety.

3-Hydroxyisoindolin-1-ones 1 are interesting heterocyclic compounds as they are present in numerous of natural products and pharmaceutical molecules with a broad spectrum of biological activities. Natural products carrying 3-hydroxyisoindolin-1-one cores include entonalactam C and fumadensine.^1^ These scaffolds have also been found in a commercial drug namely chlortalidone (Fig. 1). Besides, 3-hydroxyisoindolin-1-ones 1 are versatile precursors in the synthesis of various compounds.^2–4^ Due to their wide range of pharmaceutical activities and synthetic applications, great attention has being devoted to develop efficient strategies to access 3-hydroxyisoindolin-1-ones.

A variety of 3-hydroxyisoindolin-1-ones 1 were synthesized through selective addition of organometallic reagents (for example RMgX, RLi or R_2_Zn)^5–8^ as well as reduction^9,10^ of phthalimides. The secondary benzamides and aldehydes have been exploited as the starting materials to generate the hydroxyisoindolin-1-ones through tandem oxidative C–H activation and annulation reactions in the presence of palladium or rhodium catalysts.^11,12^ Furthermore, the treatment of 2-alkynylbenzoic acids with primary amines has given rise to the formation of the hydroxyisoindolinones.^13^ Despite several approaches have been reported, some of these methods still suffer from several drawbacks like the application of harsh reaction conditions (such as high reaction temperature or anhydrous system), low accessibility of starting materials, poor selectivity, unsatisfactory yields as well as the requirement of expensive catalysts. In addition, most of the synthetic strategies were carried out in a small scale.

Sustainable chemistry has become an essential consideration in the chemical process due to the rising concern on the detrimental effects of chemicals and the excessive process costs. In this context, ultrasonic irradiation has emerged as one of green synthetic approach. As a sustainable technology, ultrasonic irradiation offers tremendous advantages in chemical synthesis by improving the reaction rate, yields and selectivity as well as applying less hazardous materials and milder reaction conditions.^14–17^ Recent reports demonstrated that various substituted 3-methyleneisoindolin-1-ones and isoquinolin-1(2*H*)-ones have been generated in good yields in shorter reaction time *via* metal-catalyzed-cascade reaction (comprising of Sonogashira type coupling-heterocyclization reactions) under ultrasonic irradiation.^18–20^ Due to these valuable features, the ultrasonic irradiation has been widely employed in organic synthesis and industrial process.

Our group is interested in developing efficient synthetic protocols for the synthesis of bioactive heterocycles. Based on our previous reports, the hemiaminal group of 3-hydroxyisoindolin-1-ones 1 may be generated through the nucleophilic addition reaction of primary amines into 3-alkylidenelactones.^21–23^ However, several reports demonstrated that high reaction temperature and long reaction time were required for this transformation leading to the high cost of production and environmental issues.^13,24,25^ To best of our knowledge, the scale-up of this reaction has not been reported.

To address these limitations, we decide to develop an efficient synthesis of 3-hydroxyisoindolin-1-ones 1*via* nucleophilic addition reaction under ultrasonic irradiation. We also present the multigram synthesis and the synthetic applications of our methodology to access various motifs of isoindolin-1-ones.

## Results and discussion

To achieve the optimum reaction conditions for the ultrasonic-assisted-formation of 3-hydroxyisoindolin-1-ones, we initially run the process under various conditions. For this purpose, the readily available 3-benzylidenephtalide 2a and *n*-butylamine 3a were selected as model substrates.

To our delight, performing the reaction in methanol at 60 °C gave total conversion of starting material 2a within 30 min (based on TLC monitoring) and the desired product 1a was isolated as the sole product in 60% yield (Table 1, entry 1). Moderate yields of 1a were also obtained when of ethanol, *n*-butanol and iso-amyl alcohol, dichloromethane and acetonitrile were employed as the solvent (entries 2, 4–7). The results showed that iso-propanol was the suitable solvent which furnished the product in 75% yield (entry 3). We also intrigued to perform the reaction in water. However, we did not obtain the satisfactory results presumably due to the solubility problem of the starting materials (entry 8). Upon the sonication condition, the starting material 2a was hardened at the bottom of the flask thus limiting the interaction with butylamine 3a. We still observed high amount of 2a even after 6 h of reaction. Attempt to perform the reaction in iso-propanol : water (1 : 1) only generated the product in low yields (entry 9).

**Table 1:** Optimization of the reaction conditions^a^

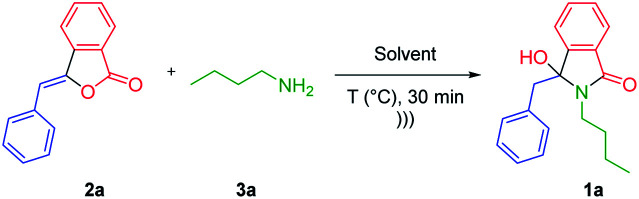				
Entry	Solvent	*T* (°C)	3a (equiv.)	Yield^b^ (%)
1	Methanol	60	2	60
2	Ethanol	60	2	54
3	Iso-propanol	60	2	75
4	*n*-Butanol	60	2	47
5	Iso-amyl alcohol	60	2	55
6	Dichloromethane	60	2	44
7	Acetonitrile	60	2	63
8	Water	60	2	<5
9	Iso-propanol : water (1 : 1)	60	2	23
10	Iso-propanol	50	2	93
11	Iso-propanol	40	2	85
12	Iso-propanol	30	2	46
13	Iso-propanol	50	1.1	80
14	Iso-propanol	50	1.5	85
15^c^	Iso-propanol	50	2	82

a The experiments were performed using 0.5 mmol of 2a under ultrasonic irradiation (47 kHz, 35 W) for 30 min.

b Isolated yields.

c The reaction was conducted at 50 °C for 5 h using conventional heating.

We observed the formation of the product 1a in excellent yield of 93% when the reaction was carried out at 50 °C (Table 1, entry 10). Lowering the temperature led to the decrease in the yields of the product 1a due to the low conversion of the starting material 2a (entries 11 and 12). Upon reducing the quantity of primary amines to 1.1 or 1.5 equiv., high isolated yields of 1a were still observed (entries 13 and 14). However, we found that low number of amines was not suitable for the less nucleophilic primary amines. In this case, longer reaction time was required to achieve total consumption of the precursor 2. We then decided to use 2 equiv. of primary amines for the scope of reaction. Compared to the conventional heating (entries 3 *vs.* 15), the ultrasound irradiation provided advantageous by reducing the reaction time (30 min *vs.* 5 h) and increasing the reaction yield (93% *vs.* 82%). The ultrasound waves may provide the mechanical effect of cavitation. They may supply energy to the reaction medium through the generation of repeating pattern of compressions and rarefactions leading to the enhancements of the reaction rates and yields.^26–28^

**Scheme 1:**
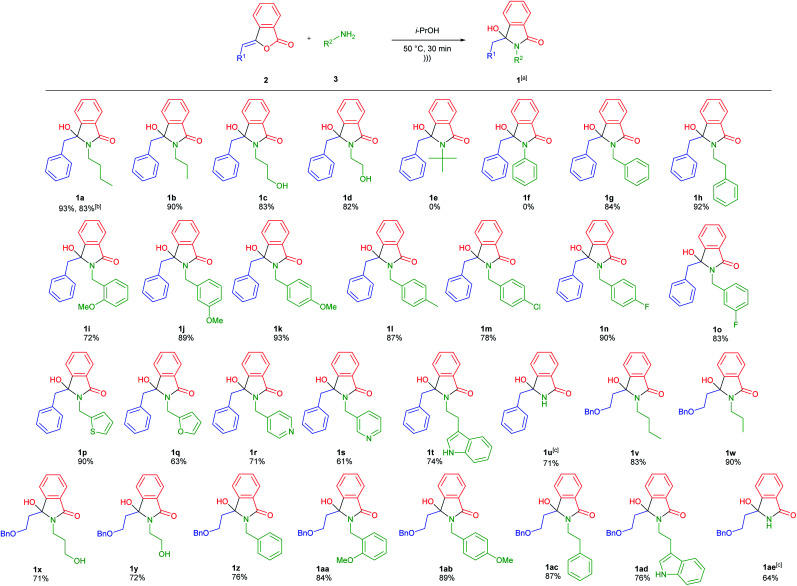
Reaction scope; ^*a*^ reagents and conditions: 2 (0.5 mmol), 3 (1 mmol, 2 equiv.), i-PrOH (2 mL), under ultrasonic irradiation (47 kHz, 35 W) at 50 °C for 30 min. ^*b*^ Reaction was conducted using 10 mmol of 2. ^*c*^ 5 equiv. of NH_4_OAc was required for the full conversion of 3-alkylidenephtalides 2.

Having obtained the optimum reaction conditions, we turned our focus to the scope of ultrasound-assisted-synthesis of 3-hydroxyisoindolin-1-ones 1 (Scheme 1). For the reaction scope, various 3-alkylidenephtalides 2 (0.5 mmol, 1 equiv.) and primary amines 3 (2 equiv.) were reacted in iso-propanol. It should be mentioned that we also utilized some bio-based primary amines^29^ including *n*-butylamine, ethanolamine, 3-amino-1-propanol and furfurylamine as well as some biogenic amines such as phenethylamine and tryptamine.

In general, the reaction was group tolerance, where 3-benzylidenephtalide 2a could smoothly react with primary amines 3 to afford diversely substituted 3-hydroxyisoindolin-1-ones 1 in good to excellent yields in short reaction time (Scheme 1). Besides the aliphatic primary amines, the aminoalcohols were tolerated under the optimized conditions and produced the corresponding lactams 1a–d in more than 80% yields. However, when *t*-butylamine and aniline were subjected to the reaction, the desired compounds 1e and 1f were not generated presumably due to the big steric hindrance and low nucleophilicity of the amines, respectively.^30^ In this case, starting material 2a was recovered at the end of reaction. Attempts to improve the reaction performance by increasing reaction time and the number of amines did not give the expected products.

We also found that the reaction was dependent on the nucleophilicity of the primary amines (1f–h).^31^ Various benzylamines bearing both electron donating and withdrawing groups at different position worked well while maintaining the reaction effectiveness (1i–o). We also managed to prepare heteroaromatic-functionalized-lactams 1p–t and *N*-unsubstituted-lactam 1u. For the latter case, 5 equiv. of NH_4_OAc was required to have complete consumption of the starting material 2a. To further explore the reaction scope, 3-alkylidenephtalide 2b namely ((*Z*)-3-(2-(benzyloxy)ethylidene)-isobenzofuran-1(3*H*)-one) was coupled with various primary amines resulted in the formation of the 3-hydroxyindolin-1-ones 1v–1ae in good yields.

The structure elucidation of all products 1 was carried out by means of ^1^H-NMR, ^13^C-NMR, HRMS and FTIR spectrometers. The formation of 3-hydroxyisoindolin-1-ones 1a–1u was confirmed by the presence of two doublet peaks at 3.00–3.70 ppm representing the benzylic protons. In the case of 1v–1ae, the methylene protons adjacent to the hemiaminal carbon give either doublet of triplets or multiplet peaks at 2.10–2.70 ppm. The appearance of the hemiaminal carbon around 90 ppm clearly demonstrated the success of our reaction. In addition, the found mass of the products at HRMS spectrum is in accordance with their corresponding calculated mass.

The scalability of this process was investigated by performing the reaction using 2.22 g (10 mmol) of 3-benzylidenephtalide 2a and *n*-butylamine 3a (20 mmol, 2 equiv.). We were pleased that the hydroxyisoindolinone 1a was isolated in 83% yields. Indeed, the large-scale synthesis under ultrasonic irradiation may allow us to reduce chemical and energy consumptions, giving our strategy an efficient character.

**Scheme 2:**
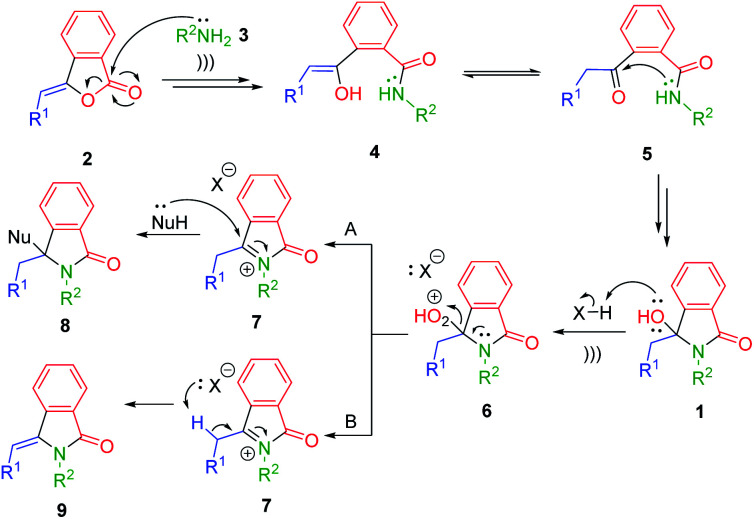
Plausible reaction mechanism for ultrasound-assisted synthesis of isoindolin-1-one derivatives.

The plausible reaction mechanism is depicted in Scheme 2. The reaction was started by nucleophilic addition of primary amines 3 into 3-alkylidenephtalides 2. Based on Table 1 (entries 3 *vs.* 13), the ultrasound irradiation might accelerate the nucleophilic addition reaction giving the enol intermediates 4 which could be tautomerized to the corresponding keto forms 5. Further intramolecular nucleophilic addition between the nitrogen amides and ketones 5 generated the desired 3-hydroxyisoindolin-1-ones 1.

By taking the advantage of 3-hydroxyisoindolin-1-ones 1 as the synthetic equivalents of the reactive *N*-acyliminium ion (NAI) intermediates 7,^32,33^ we were capable of extending our methodology to access other scaffolds of isoindoline-1-ones 8 and 9 in one-pot fashion (Scheme 2). In this context, the reaction between 3-alkylidenephtalides 2 and primary amines 3 would generate *in situ* 3-hydroxyisoindolinones 1, which in turn could be transformed into the corresponding NAI intermediates 7 under acidic condition. The generated *N*-acyliminium ions 7 could be trapped with various nucleophiles to produce 3-substituted-isoindolin-1-ones 8 (pathway A). On the other hand, the reactive intermediates 7 may undergo the β-elimination reaction in the absence of nucleophiles to give 3-alkylideneisoindolin-1-ones 9 (pathway B).

**Scheme 3:**
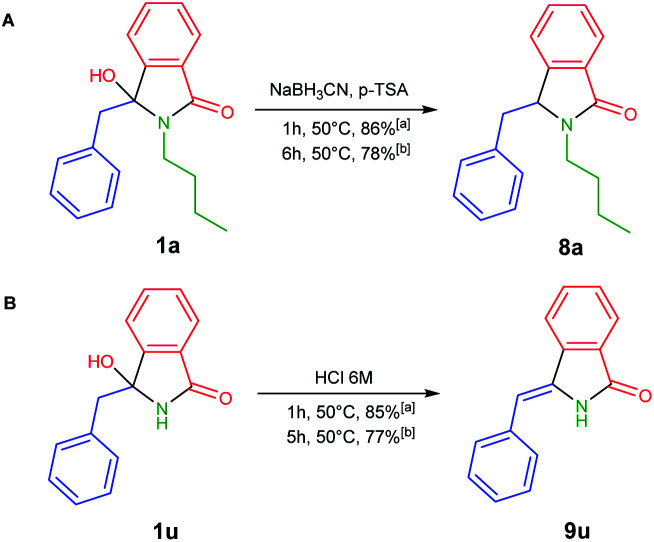
NAI reactions in (A) the presence and (B) the absence of nucleophile under (a) ultrasonic irradiation and (b) conventional heating.

To develop one-pot NAI reaction, we initially performed the NAI reaction involving 3-hydroxyisoindolin-1-ones 1a or 1u in the presence and the absence of nucleophile (NaBH_3_CN) under ultrasonic irradiation and heating process (Scheme 3). The former produced the desired products 8a or 9u in higher yields in only 1 h, whereas the complete consumption of 1 was achieved in longer reaction time by employing the latter method. The results indicated that the ultrasound irradiation might enhance the rate of NAI formation (Scheme 2).

**Scheme 4:**
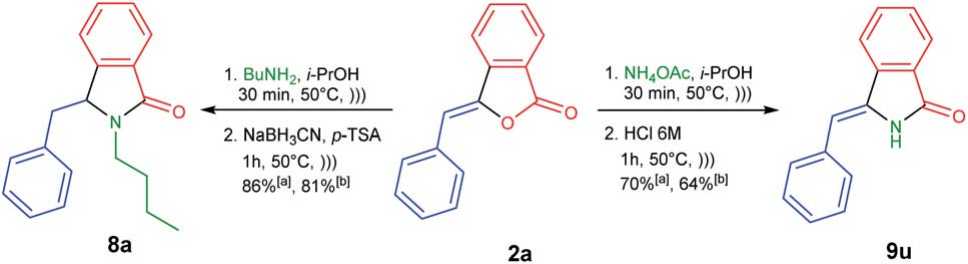
One-pot synthesis of 3-benzylisoindolin-1-one 8a and 3-benzylideneisoindolin-1-one 9u using (a) 0.5 mmol and (b) 10 mmol of 2a*via* NAI reactions.

To demonstrate the versatility of our methodology (Scheme 4), we were able to efficiently synthesize 3-benzylisoindolin-1-one 8a from readily available 3-benzylidenephtalide 2a thanks to the one-pot NAI reaction. The treatment of 2a with butylamine under ultrasonic irradiation would generate the corresponding 3-hydroxyisoindolin-1-one 1a, which in turn underwent the transformation to NAI intermediate in the presence of *p*-TSA as the acid catalyst. The resulting intermediate could be trapped with a nucleophile of NaBH_3_CN to produce 3-benzylisoindolin-1-one 8a in 86% yield.

Next, we performed one-pot NAI reaction by reacting 3-benzylidenephtalide 2a (0.5 mmol) and ammonium acetate (5 equiv.) under ultrasound irradiation for 30 min (Scheme 4). After the complete consumption of 2a, we sequentially added the aqueous solution of HCl 6 M to the reaction mixture and continued the reaction for 1 h. We are pleased that the one-pot reaction produced 3-benzylideneisoindolin-1-one 9u in 70% yield. Based on the NOESY analysis, *Z*-isomer was obtained as the sole product. It should be noted that 3-alkylideneisoindolin-1-ones 9 are important intermediates in the total synthesis of natural products, such as lennoxamine.^34^

Encouraged by these results, we then conducted the one-pot NAI reactions by using 10 mmol (2.22 g) of 3-benzylidenephtalide 2a (Scheme 4). To our satisfaction, the one-pot reaction can be scaled up without significantly reduced the reaction yield of 8a and 9u.

## Conclusions

In summary, we have developed a facile method to access isoindolin-1-ones under ultrasonic irradiation. These nitrogen heterocycles can also be prepared in multigram scale. It should be highlighted that our synthetic method can be employed to efficiently produce various motifs of isoindolin-1-ones which are important building blocks in medicinal and natural product chemistry. Further study on the functionalization of isoindolin-1-ones through the one-pot NAI reaction is underway.

## Experimental section

### General procedures for the synthesis of 3-hydroxyisoindolin-1-ones (1)

(*Z*)-3-Alkylideneisobenzofuran-1(3*H*)-ones 2 (0.5 mmol, 1 equiv.) was dissolved in 2 mL of iso-propanol. Next, primary amines 3 (1 mmol, 2 equiv.) was added to the solution. The mixture was placed in the ultrasonic bath and the reaction was conducted at 50 °C for 30 min. The mixture was partitioned with ethyl acetate (10 mL) and distilled water (10 mL). The aqueous layer was then extracted with ethyl acetate (3 × 10 mL). The combined organic layer was washed with brine (2 × 10 mL), dried over Na_2_SO_4_, filtered and concentrated *in vacuo*. The pure product was obtained from column chromatography of the crude mixture using *n*-hexane/ethyl acetate gradient system.

### Synthesis of 3-benzyl-2-butylisoindolin-1-one (8a)

(*Z*)-3-Benzylideneisobenzofuran-1(3*H*)-one 2a (111 mg, 0.5 mmol, 1 equiv.) and *n*-butylamine (0.099 mL, 1 mmol, 2 equiv.) was dissolved in 2 mL of iso-propanol. The flask was placed in the pre-heated ultrasonic bath (50 °C) and the reaction was carried out for 30 min. The reaction mixture was placed in the ice bath, followed with the addition of NaBH_3_CN (314 mg, 5 mmol, 10 equiv.) and *p*-toluene sulfonic acid monohydrate (950 mg, 5 mmol, 10 equiv.). The reaction was continued under ultrasonic irradiation at 50 °C for 1 h. The reaction was quenched with the addition of the saturated aqueous solution of NaHCO_3_, followed with the extraction with dichloromethane (3 × 5 mL). The combined organic layer was washed with brine, dried over Na_2_SO_4_, filtered and removed under vacuum. The crude product was purified by column chromatography using the eluent of *n*-hexane/ethyl acetate (9 : 1).

### Synthesis of (*Z*)-3-benzylideneisoindolin-1-one (9u)

In 25 mL of round-bottomed-flask was added (*Z*)-3-benzylideneisobenzofuran-1(3*H*)-one 2a (111 mg, 0.5 mmol, 1 equiv.), ammonium acetate (193 mg, 2.5 mmol, 5 equiv.) and iso-propanol (2 mL). The mixture was reacted under ultrasonic irradiation at 50 °C for 30 min. The flask was then placed in the ice bath and the aqueous solution of HCl (6 M, 1.67 mL, 10 mmol, 20 equiv.) was then subjected to the mixture. The reaction was continued under ultrasonic irradiation at 50 °C for 1 h. After the consumption of the intermediate 1u (based on TLC analysis), ethyl acetate (10 mL) and distilled water (10 mL) were added to the cooled reaction mixture. The aqueous phase was extracted with ethyl acetate (3 × 10 mL). The combined organic phase was washed with brine, dried over Na_2_SO_4_, filtered and evaporated to dryness under reduced pressure. The desired product 9u was isolated by column chromatography using the mixture of *n*-hexane/ethyl acetate (7 : 3).

## Author contributions

Conceptualization, MIDM and LC; methodology and analysis, MFH, IMP, NAM, MS, and MIDM; supervision, MIDM, P and BP; writing-draft preparation, MIDM and LC; writing-review and editing, MIDM and LC. All authors have read the manuscript.

## Conflicts of interest

There are no conflicts to declare.

## Supplementary Material

RA-012-D2RA02720H-s001
