# Supplementary material for: Ultrasonic-assisted-synthesis of isoindolin-1-one derivatives
Source: RSC Adv. 2022 Jun 29;12(29):19016–21. doi: 10.1039/d2ra02720h (PMC9241359; doi:10.1039/d2ra02720h)
Supplement: RA-012-D2RA02720H-s001 [file RA-012-D2RA02720H-s001.pdf]

Electronic Supplementary Material (ESI) for RSC Advances.
This journal is © The Royal Society of Chemistry 2022

Supporting Information

Ultrasonic-Assisted-Synthesis of Isoindolin-1-one Derivatives

Muhammad Idham Darussalam Mardjan,*[a] Muhamad Fadhly Hariadi,[a] Indah Mutiara Putri,[a] Nilna Amalia Musyarrofah,[a] Muflihah Salimah,[a] Priatmoko,[a] Bambang Purwono,[a] and Laurent Commeiras[b]

[a]Department of Chemistry, Faculty of Mathematics and Natural Sciences, Universitas Gadjah Mada, Yogyakarta 55281, Indonesia

[b]Aix Marseille Univ, CNRS, Centrale Marseille, iSm2, Marseille, France

e-mail: idham.darussalam@ugm.ac.id

General Method

All the experiments were carried out under argon atmosphere in ultrasonic bath (Branson 1210, 47 kHz, 35 W). While the chemicals (Merck) were used without further purification, the solvents for the synthetic purposes were distilled from calcium hydride. The analytical thin layer chromatography was conducted on Merck Kieselgel 60 F254 plates and was observed under UV light (254 nm). The column chromatography was performed on Merck Kiesgel 60 (0.040-0.063 nm).

Melting point was determined by means of electrothermal apparatus Electrothermal 9100. The NMR spectra were recorded on JEOL JNM-ECZ500R (at 500 MHz for ^{1}H and 125 MHz for ^{13}C), Bruker AC400 (at 400 MHz for ^{1}H and 100 MHz for ^{13}C) and Bruker AC300 (at 300 MHz for ^{1}H and 75 MHz for ^{13}C). The chemical shifts were reported in parts per million (ppm) relative to the internal solvent signal of CDCl3 (δ_H 7.26 ppm and δ_C 77.16 pm) or DMSO-d6 (δ_H 2.50 ppm and δ_C 39.52 pm). Multiplicity is indicated as follows: s (singlet), d (doublet), t (triplet), m (multiplet), ddd (doublet of doublet of doublet), dt (doublet of triplet), td (triplet of doublet) and br s (broad singlet). The ^{1}H and ^{13}C NMR spectra were assigned using analyses of DEPT, COSY and HMQC. The high-resolution mass spectrometry experiments were conducted using a QSTAR Elite mass spectrometer (Applied Biosystems SCIEX) or a SYNAPT G2 HDMS mass spectrometer (Waters) equipped with an electrospray ionization source operated in the positive ion mode. The IR spectra were obtained from Shimadzu Prestige-21, where the samples were prepared as KBr pellets.

General procedure for the synthesis of (Z)-3-Alkylideneisobenzofuran-1(3H)-ones (2)

Into the solution of 2-iodobenzoic acid (248 mg, 1 mmol, 1 equiv.) in DMSO (4 mL) was added $NaHCO_3$ (168 mg, 2 mmol, 2 equiv.). The mixture was carefully degassed at 25 °C for 3x10 min and backfilled with argon. Then, terminal alkynes (2 mmol, 2 equiv.) and CuI (95 mg, 0.5 mmol, 0.5 equiv.) were respectively introduced to the reaction mixture. The mixture was rapidly degassed and backfilled with argon. The reaction was performed under ultrasonic irradiation at 25 °C for 3h. The saturated solution of ammonium acetate as well as ethyl acetate was added to the mixture to quench the reaction. The mixture was then filtered over a pad of celite®, following with the extraction of the filtrate using ethyl acetate (3x20 mL). The combined organic layer was washed with brine, dried over Na_2SO_4, evaporated under reduced pressure. The crude product was subjected to column chromatography on silica gel using eluent of *n*-hexane/ethyl acetate (95:5).

(Z)-3-benzylideneisobenzofuran-1(3H)-one (2a)

Following the procedure for the synthesis of (*Z*)-3-Alkylideneisobenzofuran-1(3*H*)-ones using phenylacetylene (0.22 mL, 2 mmol, 2 equiv.) with purification using column chromatography (*n*-hexane/ethyl acetate, 95:5), afforded the desired compound **2a.**

Light yellow solid; yield: 181 mg (82%); mp: 90-95°C.

IR (KBr): 3063, 1767, 1666, 1474, 1273, 1088, 980, 756, 687 cm^{-1}.

^{1}H NMR ($CDCl_3$, 300 MHz): δ = 7.78 (d, *J* = 9 Hz, 1H, CH_{Ar}), 7.69 (d, *J* = 9 Hz, 2H, CH_{Ar}), 7.59 (t, *J* = 12 Hz, 1H, CH_{Ar}), 7.58 (d, *J* = 12 Hz, 1H, CH_{Ar}), 7.38 (t, *J* = 9 Hz, 1H, CH_{Ar}), 7.59 (t, *J* = 9 Hz, 2H, CH_{Ar}), 7.19-7.11 (m, 1H, CH_{Ar}), 6.26 (s, 1H, -CH=).

^{13}C NMR ($CDCl_3$, 75 MHz): δ = 167.1 (C=O), 144.4 (-C=), 140.6 (C_{Ar}), 134.5 (CH_{Ar}), 133.8 (CH_{Ar}), 130.2 (2CH_{Ar}), 129.8 (CH_{Ar}), 128.8 (2CH_{Ar}), 128.4 (CH_{Ar}), 125.5 (C_{Ar}), 123.4 (C_{Ar}), 119.9 (CH_{Ar}), 107.1 (-CH=).

HRMS (ESI): m/z $[M+H]^+$ cald for $C_{15}H_{11}O_2^+$: 223.0754, found: 223.0751.

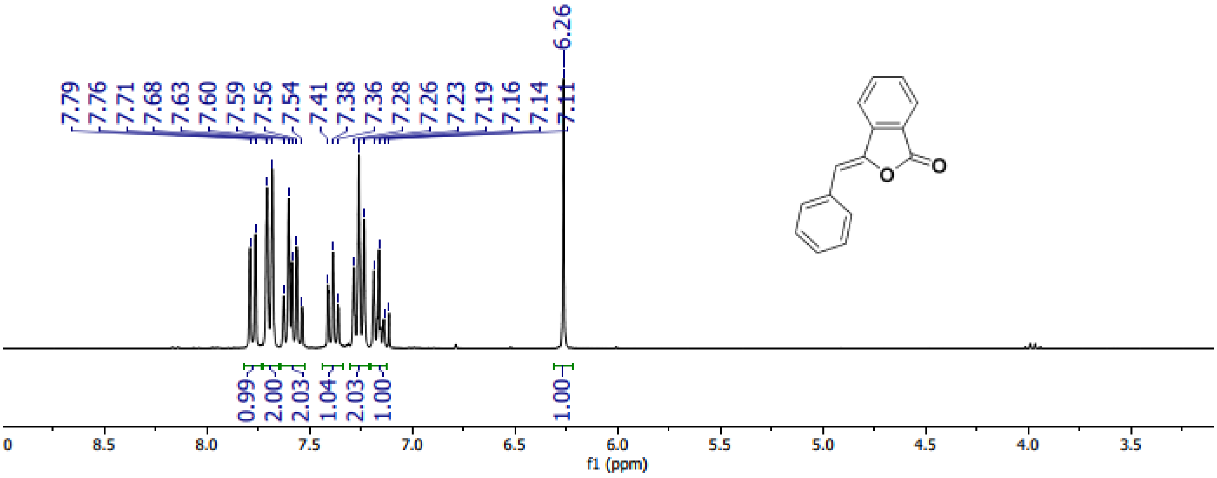
7.79
7.76
7.71
7.68
7.63
7.60
7.59
7.56
7.54
7.41
7.38
7.36
7.28
7.26
7.23
7.19
7.16
7.14
7.11
6.26
0.99
2.00
2.03
1.04
2.03
1.00
1.00
f1 (ppm)

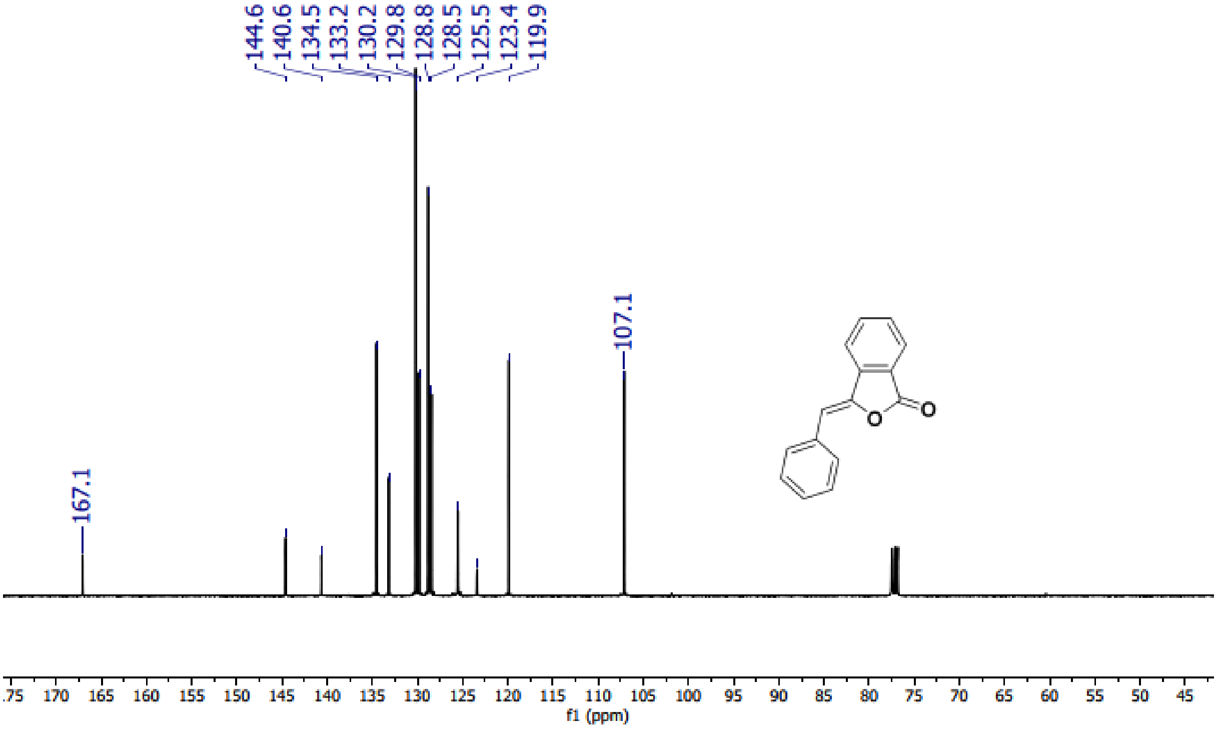
144.6
140.6
134.5
133.2
130.2
129.8
128.8
128.5
125.5
123.4
119.9
107.1
167.1
f1 (ppm)

(*Z*)-3-(2-(benzyloxy)ethylidene)isobenzofuran-1(3*H*)-one (2b)

Following the procedure for the synthesis of (*Z*)-3-Alkylideneisobenzofuran-1(3*H*)-ones using benzyl propargyl ether (0.29 mL, 2 mmol, 2 equiv.) with purification using column chromatography (*n*-hexane/ethyl acetate, 95:5), afforded the desired compound **2b.**

Light yellow solid; yield: 210 mg (79%); mp: 101-103°C.

IR (KBr): 3039, 1782, 1689, 1473, 1273, 1056, 979, 740, 694 cm^{-1}.

^{1}H NMR ($CDCl_3$, 500 MHz): δ = 7.87 (d, *J* = 7.5 Hz, 1H, CH_{Ar}), 7.69-7.62 (m, 2H, CH_{Ar}), 7.52 (t, *J* = 7.5 Hz, 1H, CH_{Ar}), 7.35-7.31 (m, 4H, CH_{Ar}), 7.28-7.24 (m, 1H, CH_{Ar}), 5.76 (t, *J* = 7 Hz, 1H, -CH=), 4.56 (s, 2H, CH_2), 4.46 (d, *J* = 7 Hz, 2H, CH_2).

^{13}C NMR ($CDCl_3$, 125 MHz): δ = 166.6 (C=O), 146.9 (-C=), 139.1 (C_{Ar}), 137.9 (C_{Ar}), 134.7 (CH_{Ar}), 130.1 (CH_{Ar}), 128.7 ($2CH_{Ar}$), 128.1 ($2CH_{Ar}$), 127.9 (CH_{Ar}), 125.5 (CH_{Ar}), 124.7 (C_{Ar}), 120.4 (CH_{Ar}), 104.8 (-CH=), 72.8 (CH_2), 64.2 (CH_2).

HRMS (ESI): m/z $[M+NH_4]^+$ cald for $C_{17}H_{18}NO_3^+$: 284.1281, found: 284.1281.

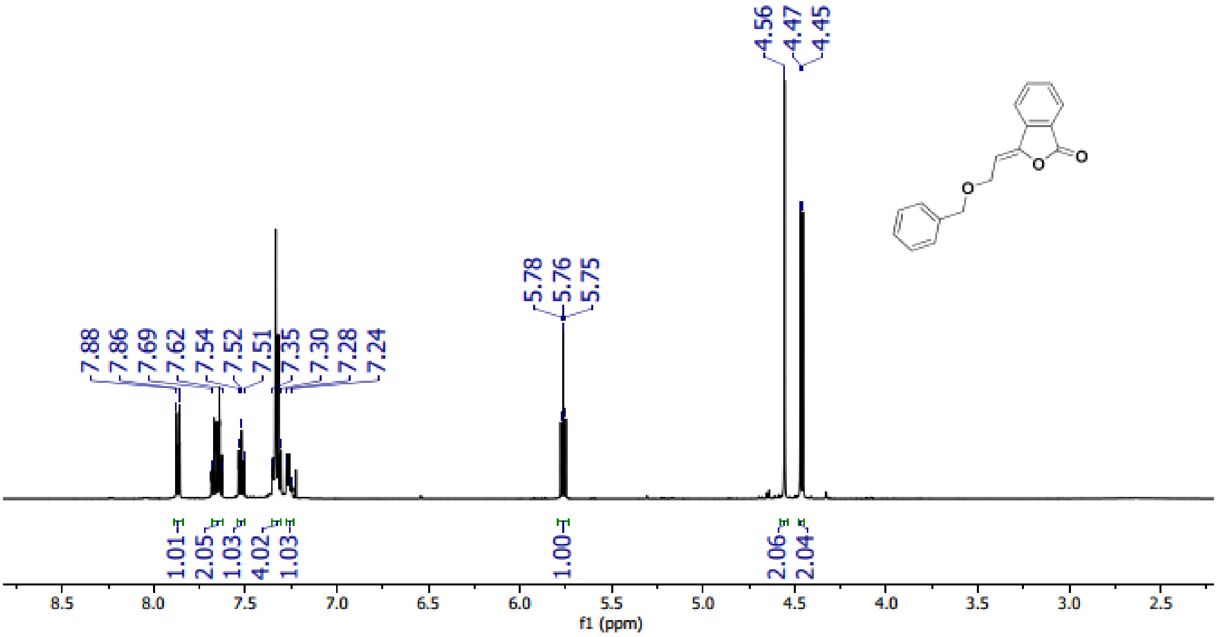
7.88
7.86
7.69
7.62
7.54
7.52
7.51
7.35
7.30
7.28
7.24
5.78
5.76
5.75
4.56
4.47
4.45
1.01
2.05
1.03
4.02
1.03
1.00
2.06
2.04
8.5
8.0
7.5
7.0
6.5
6.0
5.5
5.0
4.5
4.0
3.5
3.0
2.5
f1 (ppm)
O
O
O

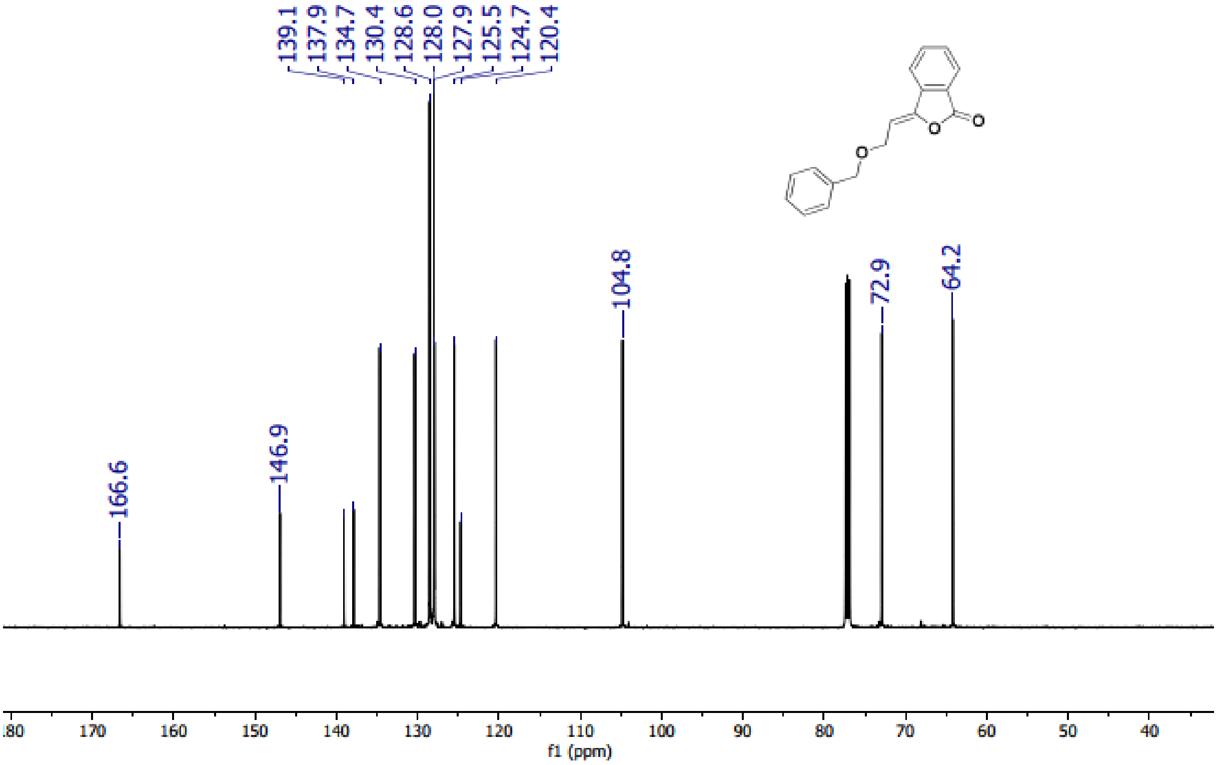
139.1
137.9
134.7
130.4
128.6
128.0
127.9
125.5
124.7
120.4
166.6
146.9
104.8
72.9
64.2
170
160
150
140
130
120
110
100
90
80
70
60
50
40
f1 (ppm)
O
O
O

General procedures for the synthesis of 3-hydroxyisoindolin-1-ones (1)

(*Z*)-3-Alkylideneisobenzofuran-1(3*H*)-ones (3-alkylidenephtalides) **2** (0.5 mmol, 1 equiv.) was dissolved in 2 mL of *iso*-propanol. Next, primary amines **3** (1 mmol, 2 equiv.) was added to the solution. The mixture was placed in the ultrasonic bath and the reaction was conducted at 50 °C for 30 min. The mixture was partitioned with ethyl acetate (10 mL) and distilled water (10 mL). The aqueous layer was then extracted with ethyl acetate (3x10 mL). The combined organic layer was washed with brine (2x10 mL), dried over Na_2SO_4, filtered and concentrated in vacuo. The pure product was obtained from column chromatography of the crude mixture using *n*-hexane/ethyl acetate gradient system.

3-Benzyl-2-butyl-3-hydroxyisoindolin-1-one (1a)

Following the procedure for the synthesis of 3-hydroxyisoindolin-1-ones **1** using (*Z*)-3-benzylideneisobenzofuran-1(3*H*)-one **2a** (111 mg, 0.5 mmol, 1 equiv.) and *n*-butylamine (0.099 mL, 1 mmol, 2 equiv.) with purification using column chromatography (*n*-hexane/ethyl acetate, 70:30), afforded the desired compound **1a.**

For scaled up synthesis of **1a**, the same procedure (small scale) of **1a** was conducted by using 2,22 g of (*Z*)-3-benzylideneisobenzofuran-1(3*H*)-one **2a** (10 mmol, 1 equiv.) and 1,98 mL of *n*-butylamine (20 mmol, 2 equiv.).

Small scale: white solid; yield: 137 mg (93%); mp: 153-155°C.

Scaled up synthesis: white solid; 2.45 g (83%), mp: 153-156 °C.

IR (KBr): 3318, 3032, 2955, 1690, 1420, 1072, 764, 702 cm^{-1}.

^{1}H-NMR ($CDCl_3$, 500 MHz): δ = 7.59 (dd, *J* = 6.5 and 1 Hz, 1H, CH_{Ar}), 7.43-7.36 (m, 2H, CH_{Ar}), 7.18-7.12 (m, 3H, CH_{Ar}), 7.07 (d, *J* = 6.5 Hz, 1H, CH_{Ar}), 6.94-6.92 (m, 2H, CH_{Ar}), 3.67 (dt, *J* = 14 and 8 Hz, 1H, CH_2), 3.53 (d, *J* = 14 Hz, 1H, CH_2), 3.31 (dt, *J* = 14 and 7.5 Hz, 1H, CH_2), 3.09 (d, *J* = 14 Hz, 1H, CH_2), 2.79 (br s, 1H, OH), 1.78-1.71 (m, 2H, CH_2), 1.44-1.36 (m, 2H, CH_2), 0.96 (t, *J* = 7.5 Hz, 3H, CH_3).

^{13}C-NMR ($CDCl_3$, 125 MHz): δ = 166.9 (C=O), 146.1 (C_{Ar}), 134.8 (C_{Ar}), 131.7 (C_{Ar}), 131.6 (CH_{Ar}), 130.4 (2CH_{Ar}), 129.7 (CH_{Ar}), 128.1 (2CH_{Ar}), 127.1 (CH_{Ar}), 123.2 (CH_{Ar}), 122.9 (CH_{Ar}), 91.4 (C), 43.2 (CH_2), 39.5 (CH_2), 31.7 (CH_2), 20.8 (CH_2), 14.0 (CH_3).

HR-MS (ESI): m/z $[M+Na]^+$ cald for $C_{19}H_{21}NO_2Na^+$: 318.1465, found: 318.1464.

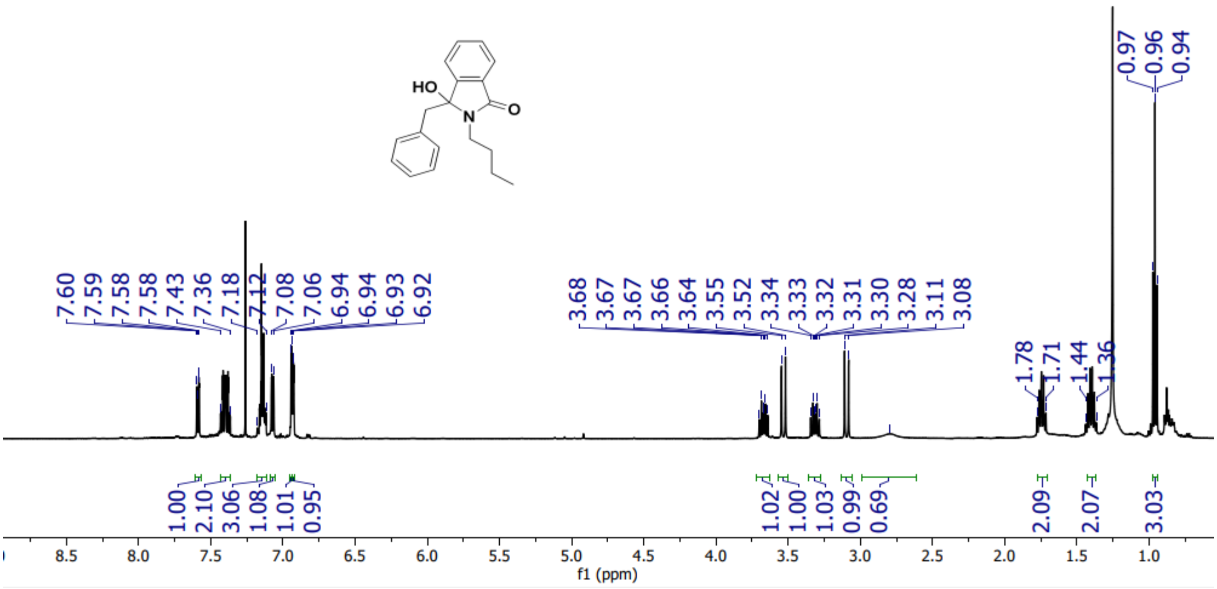

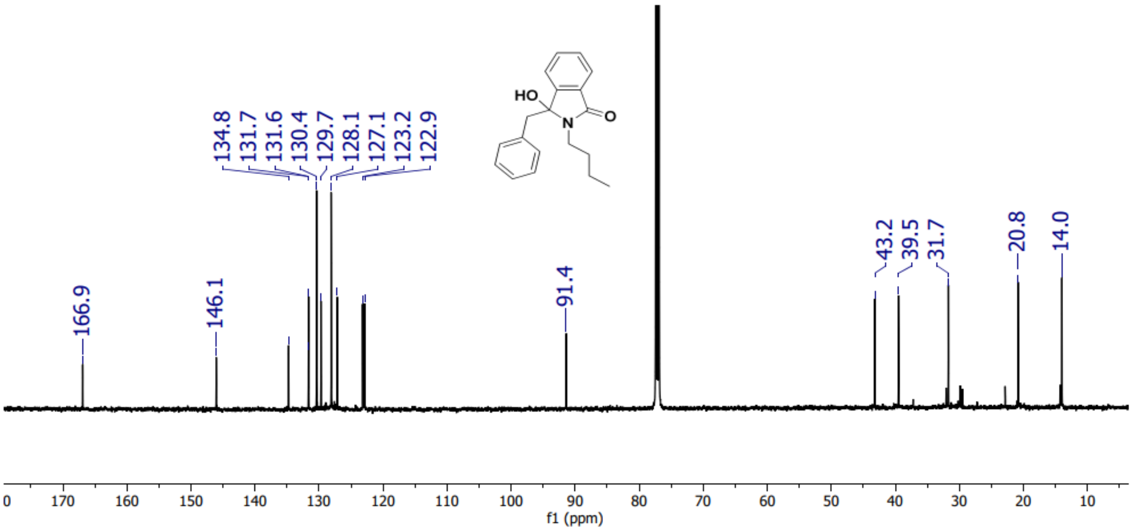

3-Benzyl-3-hydroxy-2-propylisoindolin-1-one (1b)

Following the procedure for the synthesis of 3-hydroxyisoindolin-1-ones using (*Z*)-3-benzylideneisobenzofuran-1(3*H*)-one **2a** (111 mg, 0.5 mmol, 1 equiv.) and *n*-propylamine (0.083 mL, 1 mmol, 2 equiv.) with purification using column chromatography (*n*-hexane/ethyl acetate, 70:30), afforded the desired compound **1b.**

White solid; yield: 126 mg (90%); mp: 152-155°C.

IR (KBr): 3318, 2962, 2931, 1681, 1411, 1080, 763, 702 cm^{-1}.

^{1}H-NMR ($CDCl_3$, 500 MHz): δ = 7.47 (d, *J* = 7 Hz, 1H, CH_{Ar}), 7.38 (t, *J* = 7 Hz, 1H, CH_{Ar}), 7.33 (t, *J* = 7 Hz, 1H, CH_{Ar}), 7.17-7.11 (m, 3H, CH_{Ar}), 7.03 (d, *J* = 7 Hz, 1H, CH_{Ar}), 6.93 (d, *J* = 7 Hz, 2H, CH_{Ar}), 3.58-3.52 (m, 1H, CH_2), 3.51 (d, *J* = 14 Hz, 1H, CH_2), 3;33 (br s, 1H, OH), 3.23-3.17 (m, 1H, CH_2), 3.05 (d, *J* = 14 Hz, 1H, CH_2), 1.79-1.70 (m, 2H, CH_2), 0.94 (t, *J* = 7.5 Hz, 3H, CH_3)

^{13}C-NMR ($CDCl_3$, 125 MHz): δ = 167.1 (C=O), 146.2 (C_{Ar}), 134.8 (C_{Ar}), 131.5 (CH_{Ar}), 131.4 (C_{Ar}), 130.4 (2CH_{Ar}), 129.5 (CH_{Ar}), 128.0 (2CH_{Ar}), 127.1 (CH_{Ar}), 123.1 (CH_{Ar}), 122.9 (CH_{Ar}), 91.4 (C), 43.3 (CH_2), 41.3 (CH_2), 22.7 (CH_2), 11.9 (CH_2).

HR-MS (ESI): m/z $[M+Na]^+$ cald for $C_{18}H_{19}NO_2Na^+$: 304.1308, found: 304.1309.

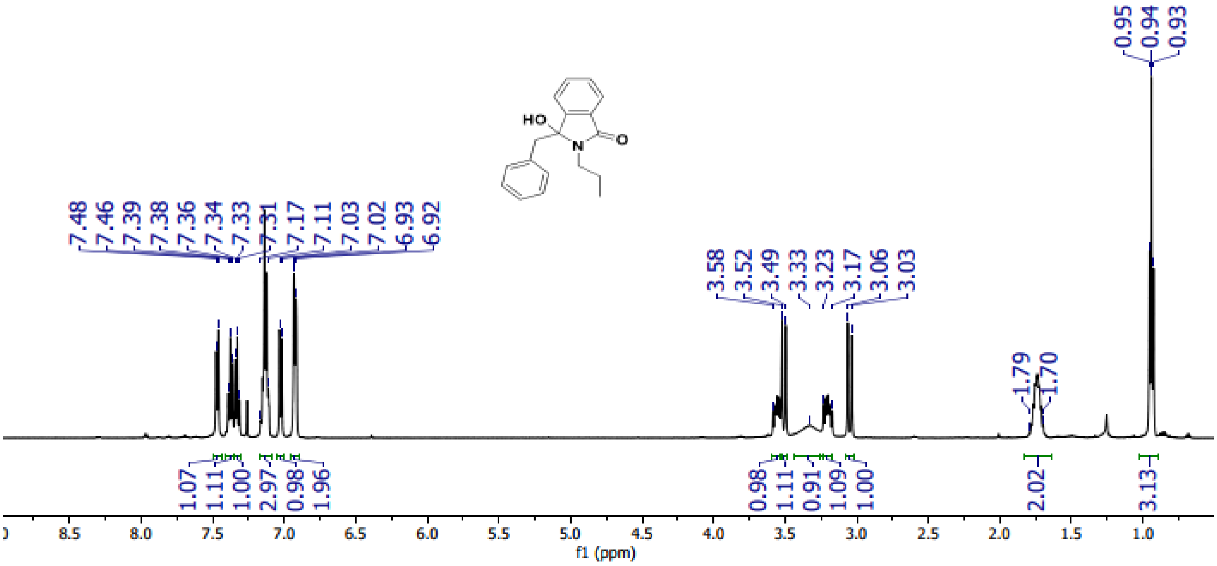
7.48
7.46
7.39
7.38
7.36
7.34
7.33
7.31
7.17
7.11
7.03
7.02
6.93
6.92
3.58
3.52
3.49
3.33
3.23
3.17
3.06
3.03
1.79
1.70
0.95
0.94
0.93
1.07
1.11
1.00
2.97
0.98
1.96
0.98
1.11
0.91
1.09
1.00
2.02
3.13
HO
N
O
f1 (ppm)

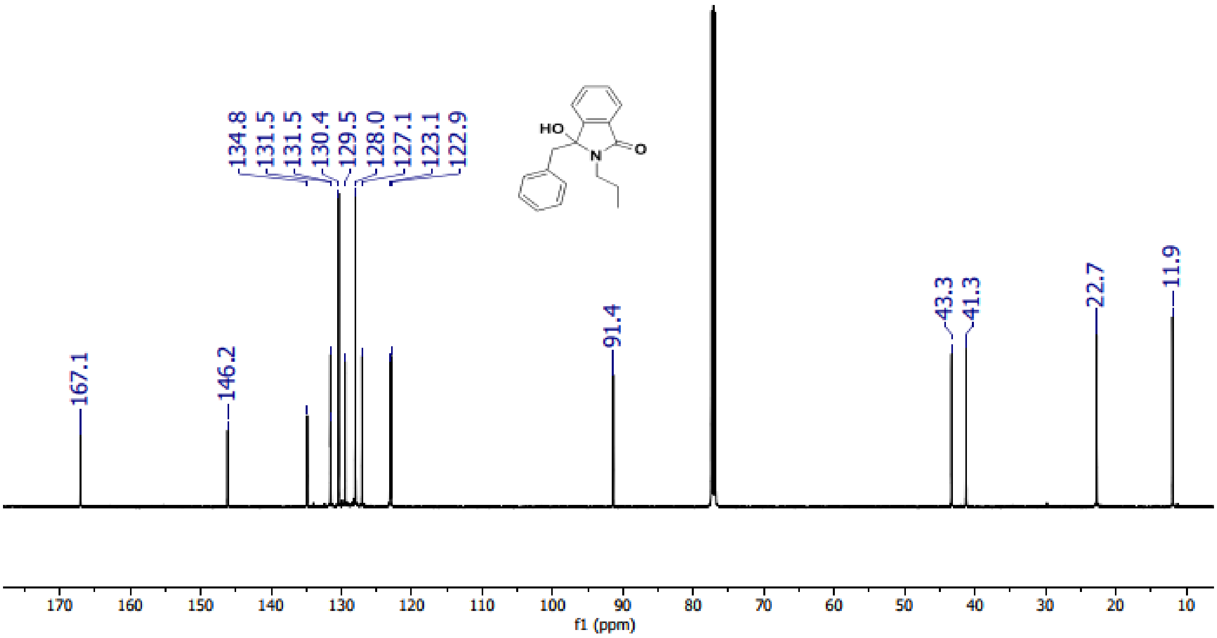
134.8
131.5
131.5
130.4
129.5
128.0
127.1
123.1
122.9
167.1
146.2
91.4
43.3
41.3
22.7
11.9
HO
N
O
f1 (ppm)

3-Benzyl-3-hydroxy-2-(3-hydroxypropyl)isoindolin-1-one (1c)

Following the procedure for the synthesis of 3-hydroxyisoindolin-1-ones **1** using (*Z*)-3-benzylideneisobenzofuran-1(3*H*)-one **2a** (111 mg, 0.5 mmol, 1 equiv.) and 3-amino-1-propanol (0.077 mL, 1 mmol, 2 equiv.) with purification using column chromatography (*n*-hexane/ethyl acetate, 30:70), afforded the desired compound **1c.**

White solid; yield: 123 mg (83%); mp: 115-119°C.

IR (KBr): 3495, 3425, 2931, 1658, 1427, 1056 cm^{-1}.

^{1}H-NMR ($CDCl_3$, 500 MHz): δ = 7.51 (d, *J* = 7.5 Hz, 1H, CH_{Ar}), 7.45 (td, *J* = 7.5 and 1 Hz, 1H, CH_{Ar}), 7.36 (t, *J* = 7.5 Hz, 1H, CH_{Ar}), 7.17 (d, *J* = 7.5 Hz, 1H, CH_{Ar}), 7.14-7.08 (m, 3H, CH_{Ar}), 6.86 (d, *J* = 7.5 Hz, 2H, CH_{Ar}), 4.52 (br s, 1H, OH), 3.83 (ddd, *J* = 14.5, 7.5 and 5 Hz, 1H), 3.67 (br s, 1H, OH), 3.58-3.48 (m, 3H, CH_2 and CH_2), 3.50 (d, *J* = 14 Hz, 1H, CH_2), 3.12 (d, *J* = 14 Hz, 1H, CH_2), 1.98-1.90 (m, 1H, CH_2), 1.87-1.79 (m, 1H, CH_2).

^{13}C-NMR ($CDCl_3$, 125 MHz): δ = 168.3 (C=O), 146.7 (C_{Ar}), 134.5 (C_{Ar}), 132.0 (CH_{Ar}), 131.0 (C_{Ar}), 130.2 (2CH_{Ar}), 129.6 (CH_{Ar}), 128.1 (2CH_{Ar}), 127.1 (CH_{Ar}), 123.1 (CH_{Ar}), 122.8 (CH_{Ar}), 91.9 (C), 59.9 (CH_2), 43.4 (CH_2), 36.2 (CH_2), 31.6 (CH_2).

HR-MS (ESI): m/z $[M+H]^+$ cald for $C_{18}H_{20}NO_3^+$: 298.1438, found: 298.1438.

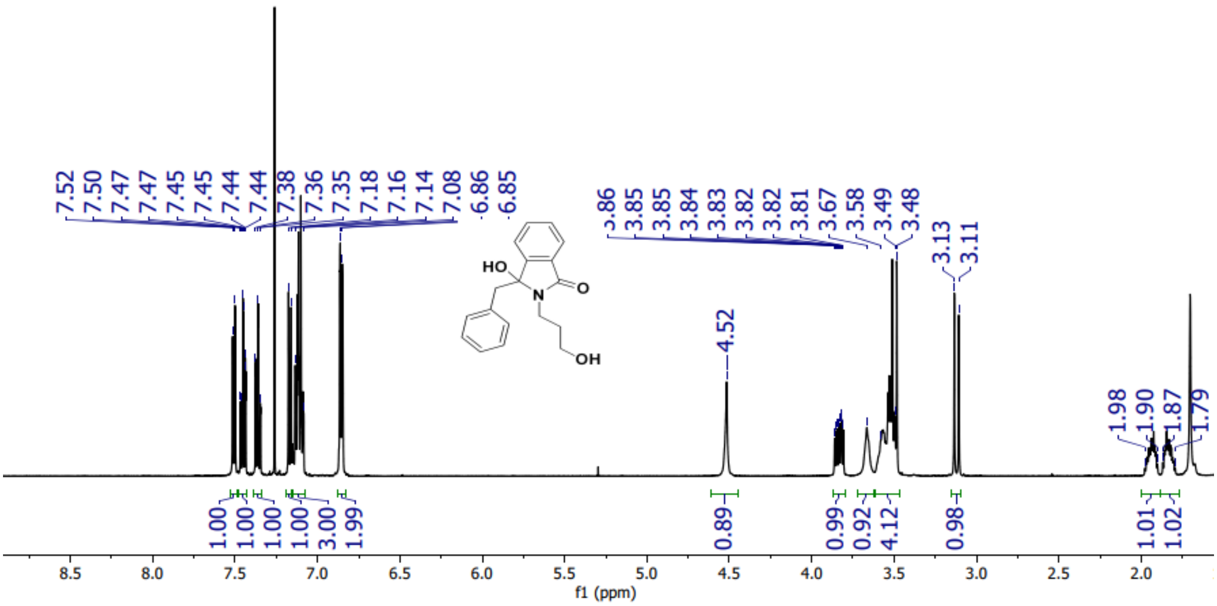
7.52
7.50
7.47
7.47
7.45
7.45
7.44
7.44
7.38
7.36
7.35
7.18
7.16
7.14
7.08
6.86
6.85
3.86
3.85
3.85
3.84
3.83
3.82
3.82
3.81
3.67
3.58
3.49
3.48
3.13
3.11
4.52
1.98
1.90
1.87
1.79
HO
N
O
OH
1.00
1.00
1.00
1.00
3.00
1.99
0.89
0.99
0.92
4.12
0.98
1.01
1.02
8.5
8.0
7.5
7.0
6.5
6.0
5.5
5.0
4.5
4.0
3.5
3.0
2.5
2.0
f1 (ppm)

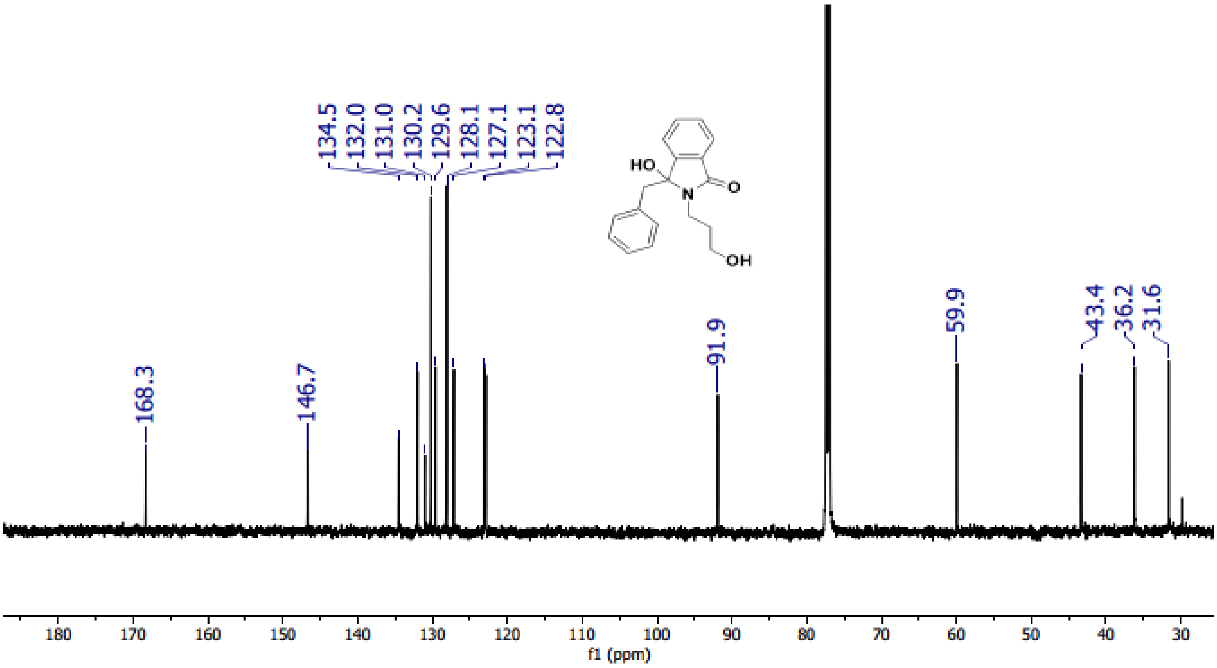
134.5
132.0
131.0
130.2
129.6
128.1
127.1
123.1
122.8
HO
N
O
OH
168.3
146.7
91.9
59.9
43.4
36.2
31.6
180
170
160
150
140
130
120
110
100
90
80
70
60
50
40
30
f1 (ppm)

3-Benzyl-3-hydroxy-2-(2-hydroxyethyl)isoindolin-1-one (1d)

Following the procedure for the synthesis of 3-hydroxyisoindolin-1-ones **1** using (*Z*)-3-benzylideneisobenzofuran-1(3*H*)-one **2a** (111 mg, 0.5 mmol, 1 equiv.) and ethanolamine (0.060 mL, 1 mmol, 2 equiv.) with purification using column chromatography (*n*-hexane/ethyl acetate, 30:70), afforded the desired compound **1d.**

White solid; yield: 116 mg (82%); mp: 114-120°C.

IR (KBr): 3471, 3286, 285, 1689, 1443, 1072, 941, 772, 702 cm^{-1}.

^{1}H-NMR ($CDCl_3$, 500 MHz): δ = 7.43 (td, *J* = 7.5 and 1 Hz, 1H, CH_{Ar}), 7.40 (d, *J* = 7.5 Hz, 1H, CH_{Ar}), 7.31 (t, *J* = 7.5, 1H, CH_{Ar}), 7.21 (d, *J* = 7.5 Hz, 1H, CH_{Ar}), 7.12-7.05 (m, 3H, CH_{Ar}), 6.79 (d, *J* = 7.5 Hz, 2H, CH_{Ar}), 3.82-3.77 (m, 1H, CH_2), 3.68-3.59 (m, 2H, CH_2), 3.42 (d, *J* = 14 Hz, 1H, CH_2), 3.35 (ddd, *J* = 14.5, 8 and 4 Hz, 1H, CH_2), 3.09 (d, *J* = 14 Hz, 1H, CH_2).

^{13}C-NMR ($CDCl_3$, 125 MHz): δ = 168.3 (C=O), 146.6 (C_{Ar}), 134.4 (C_{Ar}), 132.1 (CH_{Ar}), 130.6 (C_{Ar}), 130.0 (2CH_{Ar}), 129.4 (CH_{Ar}), 128.0 (2CH_{Ar}), 127.0 (CH_{Ar}), 123.0 (CH_{Ar}), 122.8 (CH_{Ar}), 91.0 (C), 61.0 (CH_2), 43.2 (CH_2), 42.1 (CH_2).

HR-MS (ESI): m/z $[M+H]^+$ cald for $C_{17}H_{18}NO_3^+$: 284.1281, found: 284.1281.

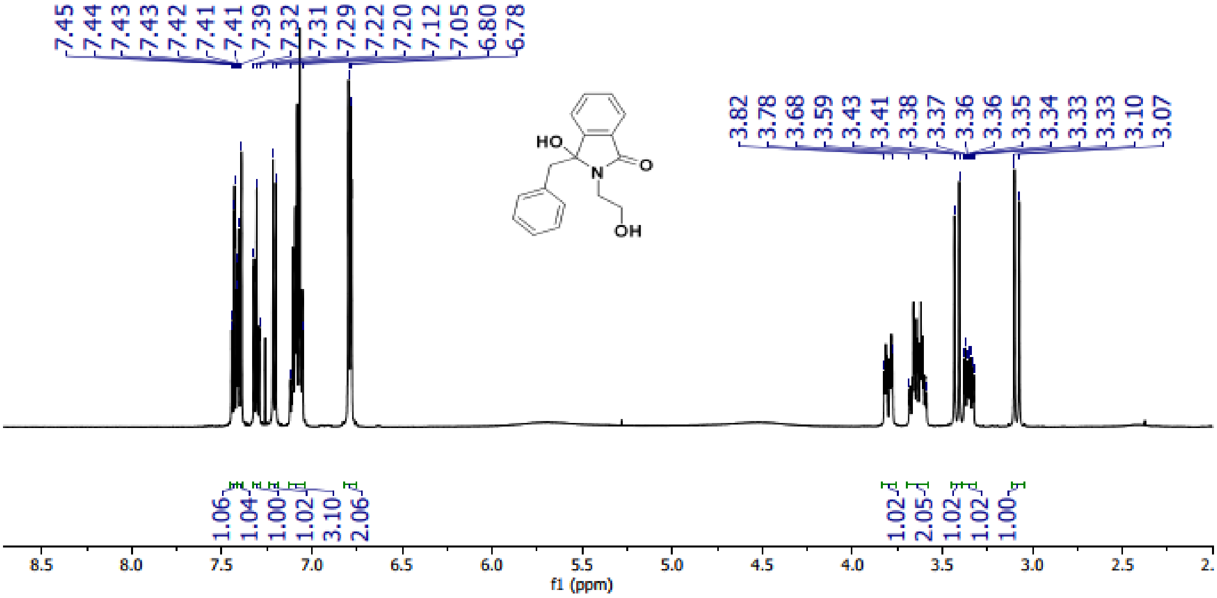
7.45 7.44 7.43 7.43 7.42 7.41 7.41 7.39 7.32 7.31 7.29 7.22 7.20 7.12 7.05 6.80 6.78
3.82 3.78 3.68 3.59 3.43 3.41 3.38 3.37 3.36 3.36 3.35 3.34 3.33 3.33 3.10 3.07
HO
N
O
OH
1.06 1.04 1.00 1.02 3.10 2.06
1.02 2.05 1.02 1.02 1.00
8.5 8.0 7.5 7.0 6.5 6.0 5.5 5.0 4.5 4.0 3.5 3.0 2.5 2.
f1 (ppm)

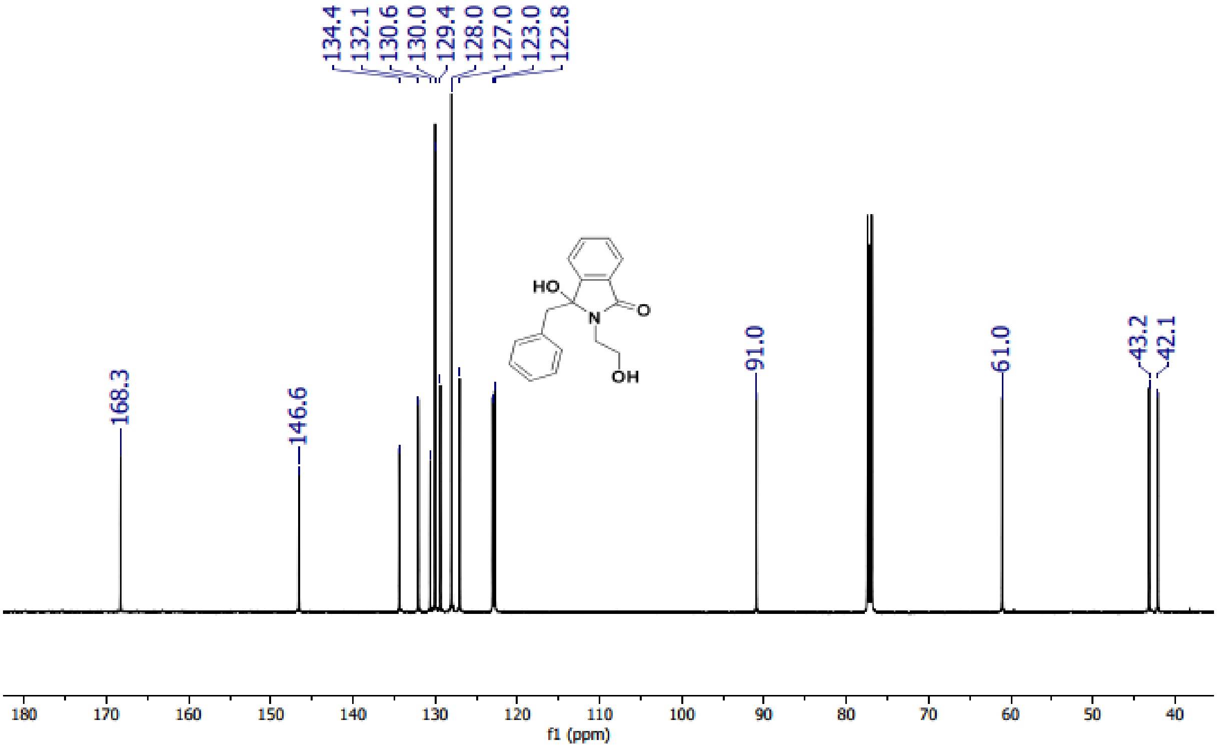
134.4 132.1 130.6 130.0 129.4 128.0 127.0 123.0 122.8
168.3
146.6
91.0
61.0
43.2 42.1
HO
N
O
OH
180 170 160 150 140 130 120 110 100 90 80 70 60 50 40
f1 (ppm)

2,3-Dibenzyl-3-hydroxyisoindolin-1-one (1g)

Following the procedure for the synthesis of 3-hydroxyisoindolin-1-ones **1** using (*Z*)-3-benzylideneisobenzofuran-1(3*H*)-one **2a** (111 mg, 0.5 mmol, 1 equiv.) and benzylamine (0.11 mL, 1 mmol, 2 equiv.) with purification using column chromatography (*n*-hexane/ethyl acetate, 50:50), afforded the desired compound **1g.**

White solid; yield: 138 mg (84%); mp: 160-165°C.

IR (KBr): 3425, 3032, 2924, 1690, 1435, 1072, 972, 694cm^{-1}.

^{1}H-NMR ($CDCl_3$, 500 MHz): δ = 7.57-7.55 (m, 1H, CH_{Ar}), 7.38 (d, *J* = 7.5 Hz, 2H, CH_{Ar}), 7.36-7.34 (m, 2H, CH_{Ar}), 7.24 (t, *J* = 7.5 Hz, 2H, CH_{Ar}), 7.20-7.09 (m, 4H, CH_{Ar}), 6.91-6.87 (m, 3H, CH_{Ar}), 4.67 (d, *J* = 15 Hz, 1H, CH_2), 4.50 (d, *J* = 15 Hz, 1H, CH_2), 3.57 (br s, 1H, OH), 3.40 (d, *J* = 14 Hz, 1H, CH_2), 2.83 (d, *J* = 14 Hz, 1H, CH_2).

^{13}C-NMR ($CDCl_3$, 125 MHz): δ = 167.3 (C=O), 146.2 (C_{Ar}), 138.5 (C_{Ar}), 134.7 (C_{Ar}), 131.7 (CH_{Ar}), 130.9 (C_{Ar}), 130.5 ($2CH_{Ar}$), 129.5 (CH_{Ar}), 128.5 ($2CH_{Ar}$), 128.3 ($2CH_{Ar}$), 127.8 ($2CH_{Ar}$), 127.2 (CH_{Ar}), 126.9 (CH_{Ar}), 123.2 (CH_{Ar}), 123.1 (CH_{Ar}), 91.4 (C), 43.9 (CH_2), 42.4 (CH_2).

HR-MS (ESI): m/z $[M+H]^+$ cald for $C_{22}H_{20}NO_2^+$: 330.1489, found: 330.1492.

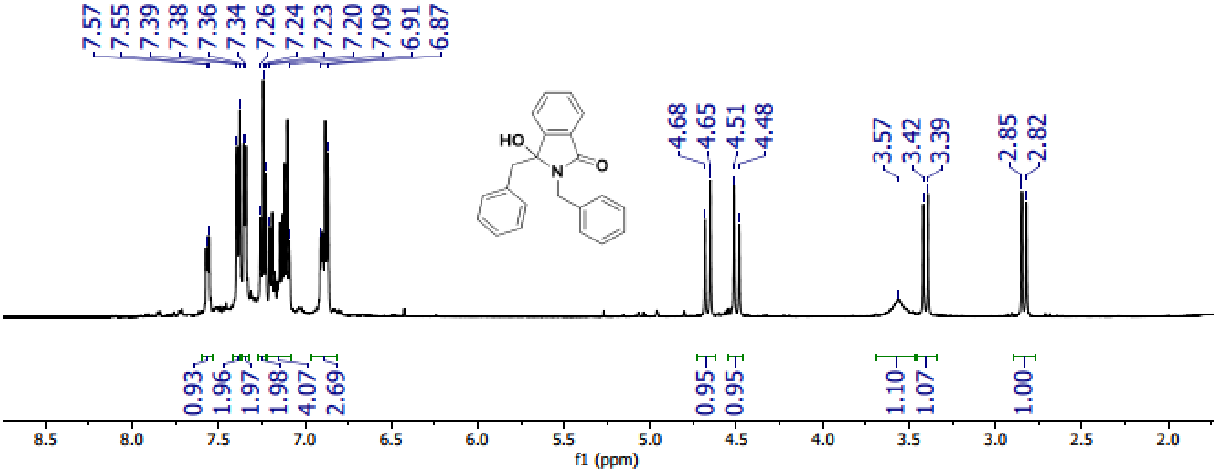
7.57
7.55
7.39
7.38
7.36
7.34
7.26
7.24
7.23
7.20
7.09
6.91
6.87
4.68
4.65
4.51
4.48
3.57
3.42
3.39
2.85
2.82
HO
N
O
0.93
1.96
1.97
1.98
4.07
2.69
0.95
0.95
1.10
1.07
1.00
8.5
8.0
7.5
7.0
6.5
6.0
5.5
5.0
4.5
4.0
3.5
3.0
2.5
2.0
f1 (ppm)

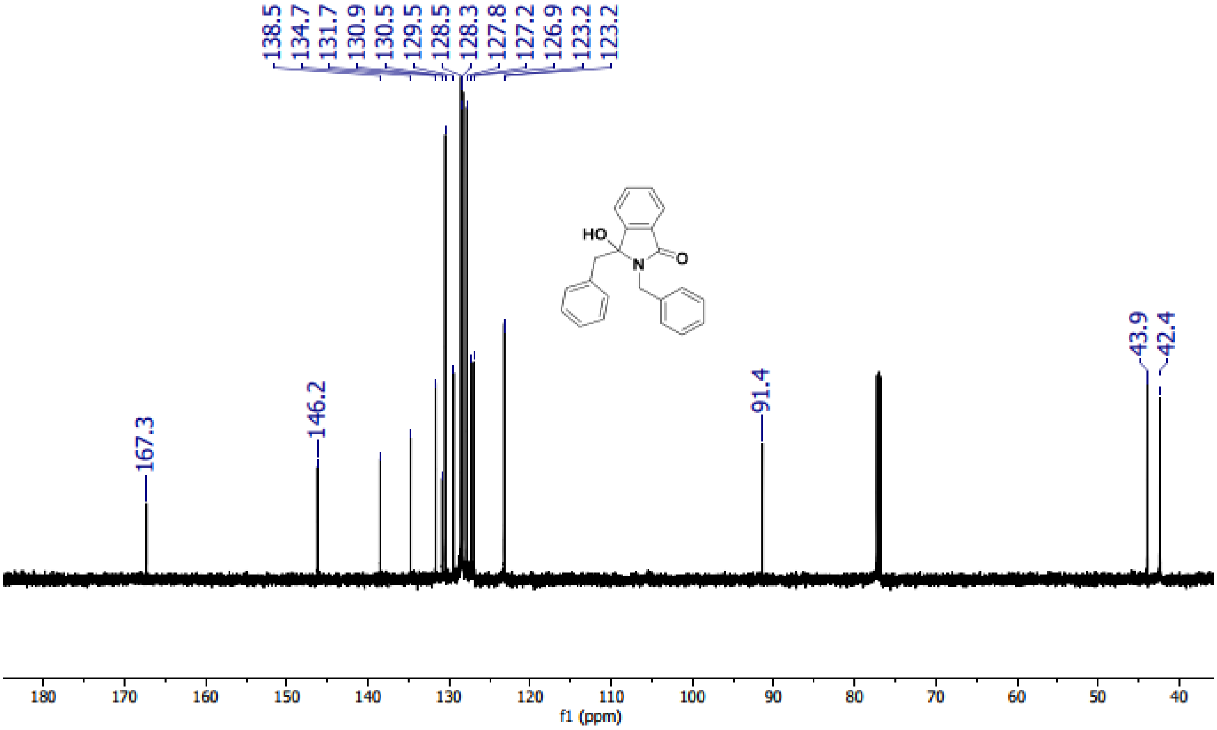
138.5
134.7
131.7
130.9
130.5
129.5
128.5
128.3
127.8
127.2
126.9
123.2
123.2
HO
N
O
167.3
146.2
91.4
43.9
42.4
180
170
160
150
140
130
120
110
100
90
80
70
60
50
40
f1 (ppm)

3-Benzyl-3-hydroxy-2-phenethylisoindolin-1-one (1h)

Following the procedure for the synthesis of 3-hydroxyisoindolin-1-ones **1** using (*Z*)-3-benzylideneisobenzofuran-1(3*H*)-one **2a** (111 mg, 0.5 mmol, 1 equiv.) and 2-phenethylamine (0.126 mL, 1 mmol, 2 equiv.) with purification using column chromatography (*n*-hexane/ethyl acetate, 50:50), afforded the desired compound **1h.**

Light brown solid; yield: 158 mg (92%); mp: 153-157°C.

IR (KBr): 3333, 3032, 2924, 1674, 1412, 1080, 756, 702 cm^{-1}.

^{1}H-NMR ($CDCl_3$, 500 MHz): δ = 7.61 (d, *J* = 7.5 Hz, 1H, CH_{Ar}), 7.41 (td, *J* = 7.5 and 1 Hz, 1H, CH_{Ar}), 7.39 (td, *J* = 7.5 and 1 Hz, 1H, CH_{Ar}), 7.31-7.25 (m, 4H, CH_{Ar}), 7.23-7.20 (m, 1H, CH_{Ar}), 7.15-7.08 (m, 4H, CH_{Ar}), 6.85 (d, *J* = 7.5 Hz, 2H, CH_{Ar}), 3.96 (ddd, *J* =14, 9 and 5 Hz, 1H, CH_2), 3.56-3.51 (m, 1H, CH_2), 3.42 (d, *J* = 14 Hz, 1H, CH_2), 3.22-3.16 (m, 1H, CH_2), 3.08 (d, *J* = 14 Hz, 1H, CH_2), 3.07-3.02 (m, 1H, CH_2).

^{13}C-NMR ($CDCl_3$, 125 MHz): δ = 167.3 (C=O), 146.5 (C_{Ar}), 139.9 (C_{Ar}), 134.8 (C_{Ar}), 132.1 (CH_{Ar}), 131.8 (C_{Ar}), 130.5 (2CH_{Ar}), 130.0 (CH_{Ar}), 129.5 (2CH_{Ar}),129.0 (2CH_{Ar}), 128.4 (2CH_{Ar}), 127.4 (CH_{Ar}), 126.9 (CH_{Ar}), 123.4 (CH_{Ar}), 123.0 (CH_{Ar}), 91.5 (C), 43.5 (CH_2), 42.0 (CH_2), 35.2 (CH_2).

HR-MS (ESI): m/z $[M+Na]^+$ cald for $C_{23}H_{21}NO_2Na^+$: 366.1465, found: 366.1464.

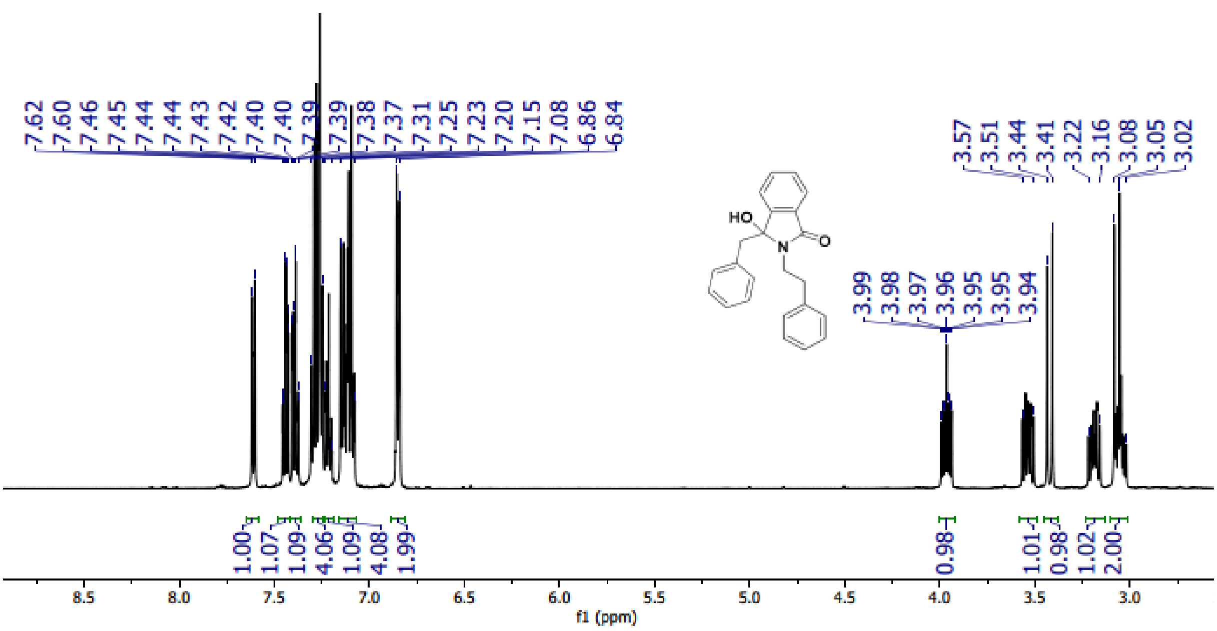

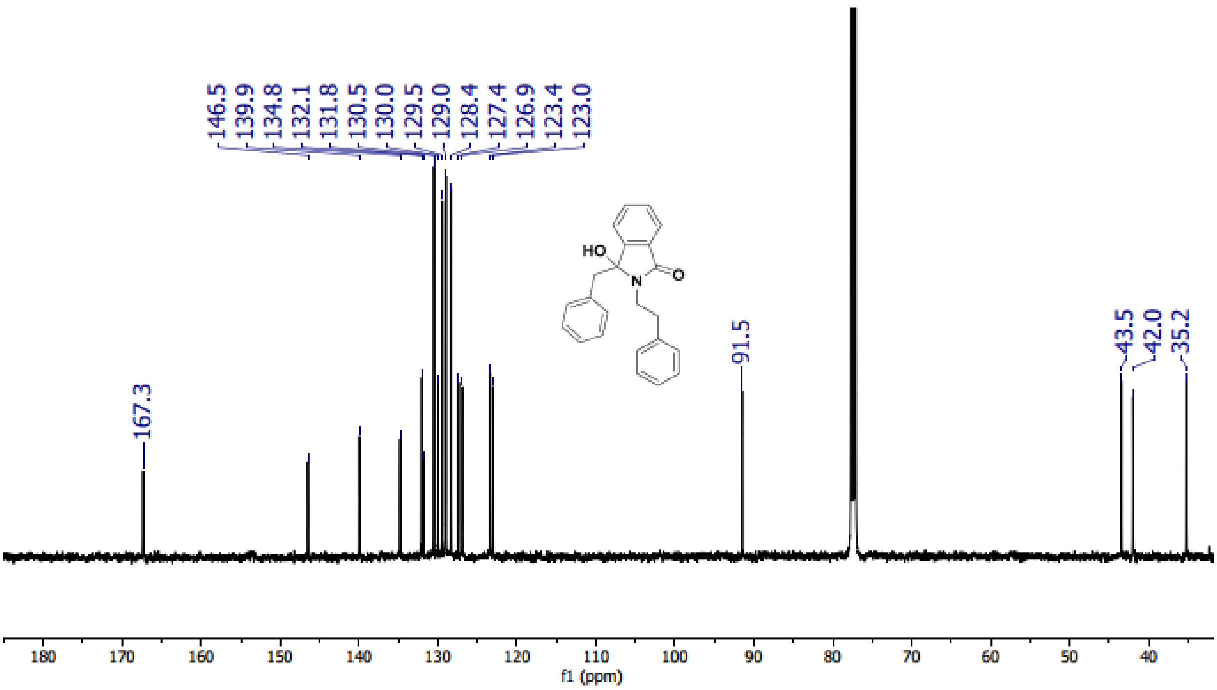

3-Benzyl-3-hydroxy-2-(2-methoxybenzyl)isoindolin-1-one (1i)

Following the procedure for the synthesis of 3-hydroxyisoindolin-1-ones **1** using (*Z*)-3-benzylideneisobenzofuran-1(3*H*)-one **2a** (111 mg, 0.5 mmol, 1 equiv.) and 2-methoxybenzylamine (0.130 mL, 1 mmol, 2 equiv.) with purification using column chromatography (*n*-hexane/ethyl acetate, 50:50), afforded the desired compound **1i.**

White solid; yield: 129 mg (72%); mp: 163-165°C.

IR (KBr): 3395, 3032, 2932, 2839, 1682, 1412, 1103, 756, 702 cm^{-1}.

^{1}H-NMR ($CDCl_3$, 400 MHz): δ = 7.64 (d, *J* = 7.5 Hz, 1H, CH_{Ar}), 7.46-7.38 (m, 3H, CH_{Ar}), 7.23 (t, *J* = 7.5 Hz, 1H, CH_{Ar}), 7.17-7.11 (m, 4H, CH_{Ar}), 6.93-6.88 (m, 4H, CH_{Ar}), 4.93 (d, *J* = 15 Hz, 1H, CH_2), 4.66 (d, *J* = 15 Hz, 1H, CH_2), 3.94 (s, 3H, CH_3), 3.52 (d, *J* = 14 Hz, 1H, CH_2), 3.16 (d, *J* = 14 Hz, 1H, CH_2).

^{13}C-NMR ($CDCl_3$, 100 MHz): δ = 167.5 (C=O), 156.7 (C_{Ar}), 146.4 (C_{Ar}), 134.9 (C_{Ar}), 131.8 (CH_{Ar}), 131.4 (C_{Ar}), 130.6 (CH_{Ar}), 130.4 (2CH_{Ar}), 129.6 (CH_{Ar}), 128.8 (CH_{Ar}), 128.1 (2CH_{Ar}), 127.1 (CH_{Ar}), 126.4 (C_{Ar}), 123.4 (CH_{Ar}), 122.9 (CH_{Ar}), 121.3 (CH_{Ar}), 110.8 (CH_{Ar}), 91.3 (C), 55.8 (CH_3), 43.9 (CH_2), 37.0 (CH_2).

HR-MS (ESI): m/z $[M+H]^+$ cald for $C_{23}H_{22}NO_3^+$: 360.1594, found: 360.1596.

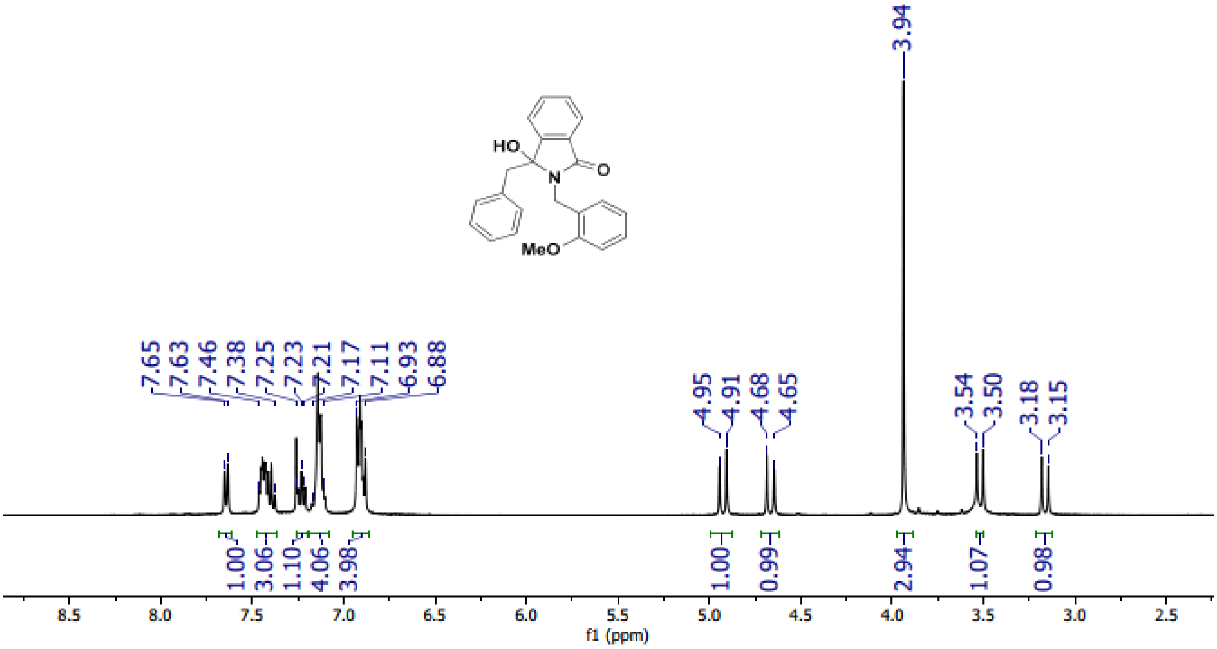
7.65
7.63
7.46
7.38
7.25
7.23
7.21
7.17
7.11
6.93
6.88
4.95
4.91
4.68
4.65
3.94
3.54
3.50
3.18
3.15
1.00
3.06
1.10
4.06
3.98
1.00
0.99
2.94
1.07
0.98
HO
N
O
MeO
f1 (ppm)

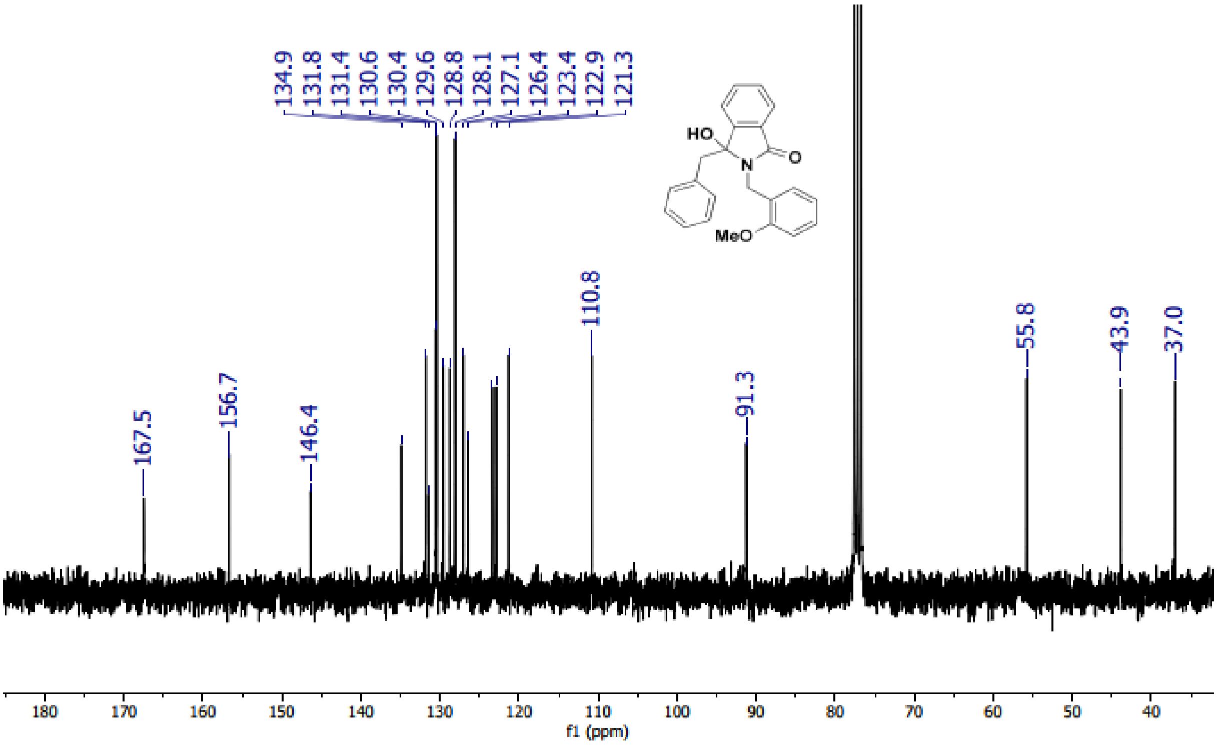
134.9
131.8
131.4
130.6
130.4
129.6
128.8
128.1
127.1
126.4
123.4
122.9
121.3
167.5
156.7
146.4
110.8
91.3
55.8
43.9
37.0
HO
N
O
MeO
f1 (ppm)

3-Benzyl-3-hydroxy-2-(3-methoxybenzyl)isoindolin-1-one (1j)

Following the procedure for the synthesis of 3-hydroxyisoindolin-1-ones **1** using (*Z*)-3-benzylideneisobenzofuran-1(3*H*)-one **2a** (111 mg, 0.5 mmol, 1 equiv.) and 3-methoxybenzylamine (0.128 mL, 1 mmol, 2 equiv.) with purification using column chromatography (*n*-hexane/ethyl acetate, 50:50), afforded the desired compound **1j.**

Yellow viscous oil; yield: 160 mg (89%).

IR (nujol): 3325, 3032, 2924, 1681, 1604, 1411, 1257, 1049, 702 cm^{-1}.

^{1}H-NMR ($CDCl_3$, 500 MHz): δ = 7.54 (d, *J* = 7.5 Hz, 1H, CH_{Ar}), 7.37-7.35 (m, 2H, CH_{Ar}), 7.18-7.10 (m, 4H, CH_{Ar}), 6.97 (d, *J* = 7.5 Hz, 1H, CH_{Ar}), 6.93-6.92 (m, 2H, CH_{Ar}), 6.88 (d, *J* = 7.5 Hz, 2H, CH_{Ar}), 6.72 (d, *J* = 7.5 Hz, 1H, CH_{Ar}), 4.62 (d, *J* = 15 Hz, 1H, CH_2), 4.47 (d, *J* = 15 Hz, 1H, CH_2), 3.94 (br s, 1H, OH), 3.70 (s, 3H, CH_3), 3.42 (d, *J* = 14 Hz, 1H, CH_2), 2.86 (d, *J* = 14 Hz, 1H, CH_2).

^{13}C-NMR ($CDCl_3$, 125 MHz): δ = 167.5 (C=O), 159.7 (C_{Ar}), 146.4 (C_{Ar}), 140.2 (C_{Ar}), 134.9 (C_{Ar}), 131.8 (CH_{Ar}), 130.9 (C_{Ar}), 130.6 ($2CH_{Ar}$), 129.6 (CH_{Ar}), 129.5 (CH_{Ar}), 127.9 ($2CH_{Ar}$), 127.0 (CH_{Ar}), 123.4 (CH_{Ar}),123.3 (CH_{Ar}), 120.7 (CH_{Ar}), 114.0 (CH_{Ar}), 112.8 (CH_{Ar}), 91.5 (C), 55.3 (CH_3), 44.0 (CH_2), 42.4 (CH_2).

HRMS (ESI): m/z $[M+Na]^+$ cald for $C_{23}H_{21}NO_3Na^+$: 382.1414, found: 382.1408.

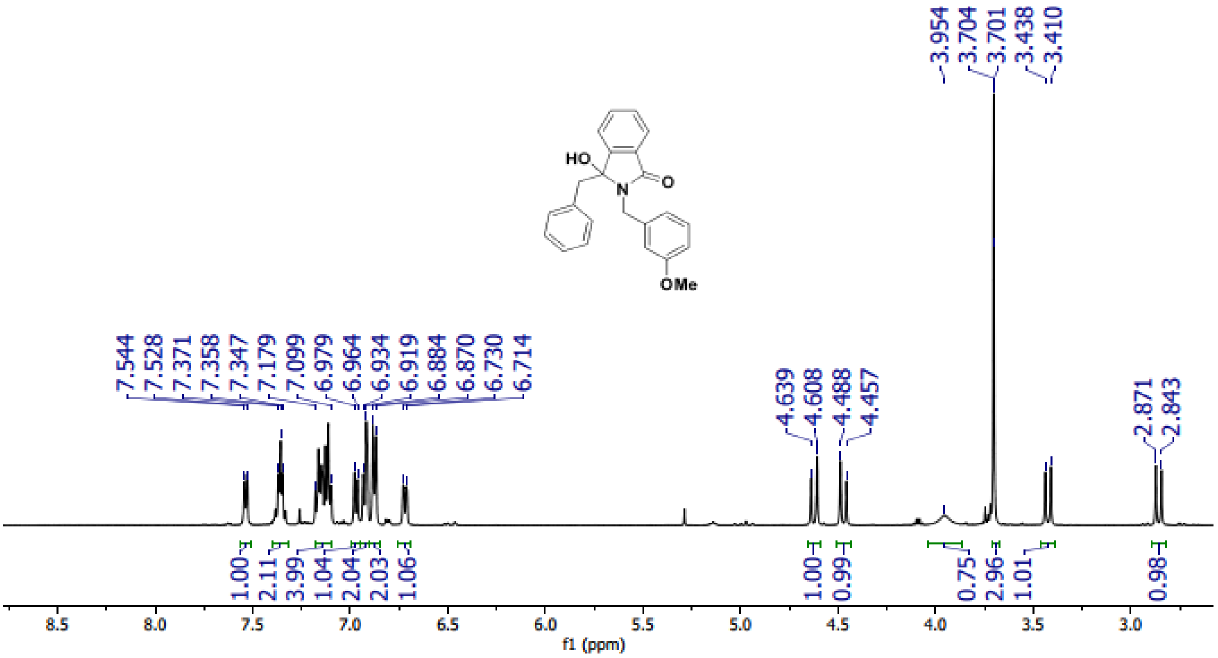
3.954
3.704
3.701
3.438
3.410
HO
N
O
OMe
7.544
7.528
7.371
7.358
7.347
7.179
7.099
6.979
6.964
6.934
6.919
6.884
6.870
6.730
6.714
4.639
4.608
4.488
4.457
2.871
2.843
1.00
2.11
3.99
1.04
2.04
2.03
1.06
1.00
0.99
0.75
2.96
1.01
0.98
8.5 8.0 7.5 7.0 6.5 6.0 5.5 5.0 4.5 4.0 3.5 3.0
f1 (ppm)

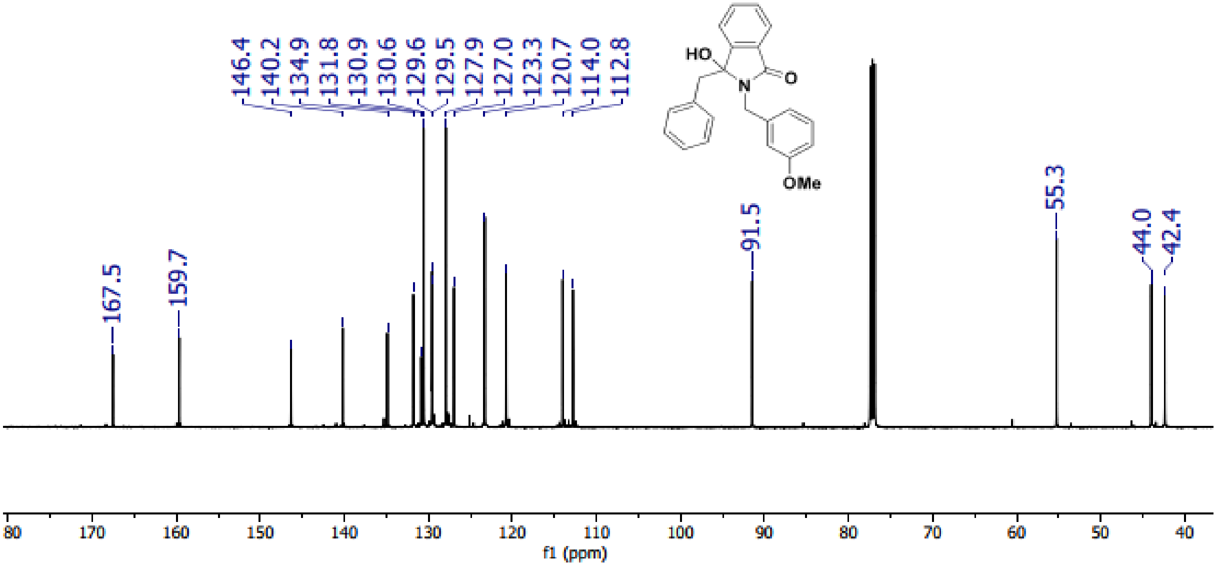
146.4
140.2
134.9
131.8
130.9
130.6
129.6
129.5
127.9
127.0
123.3
120.7
114.0
112.8
HO
N
O
OMe
167.5
159.7
91.5
55.3
44.0
42.4
80 170 160 150 140 130 120 110 100 90 80 70 60 50 40
f1 (ppm)

3-Benzyl-3-hydroxy-2-(4-methoxybenzyl)isoindolin-1-one (1k)

Following the procedure for the synthesis of 3-hydroxyisoindolin-1-ones **1** using (*Z*)-3-benzylideneisobenzofuran-1(3*H*)-one **2a** (111 mg, 0.5 mmol, 1 equiv.) and 4-methoxybenzylamine (0.130 mL, 1 mmol, 2 equiv.) with purification using column chromatography (*n*-hexane/ethyl acetate, 50:50), afforded the desired compound **1k.**

White solid; yield: 167 mg (93%); mp: 149-152°C.

IR (KBr): 3387, 2924, 2839, 1682, 1512, 1404, 1242, 1180, 1072, 756, 702cm^{-1}.

^{1}H-NMR ($CDCl_3$, 400 MHz): δ = 7.56 (d, *J* = 7 Hz, 1H, CH_{Ar}), 7.39-7.33 (m, 4H, CH_{Ar}), 7.17-7.09 (m, 3H, CH_{Ar}), 6.97-6.95 (m, 1H, CH_{Ar}), 6.88 (d, *J* = 7 Hz, 2H, CH_{Ar}), 6.75 (d, *J* = 7 Hz, 2H, CH_{Ar}), 4.66 (d, *J* = 14.8 Hz, 1H, CH_2), 4.46 (d, *J* = 14.8 Hz, 1H, CH_2), 3.72 (s, 3H, CH_3), 3.44 (d, *J* = 14 Hz, 1H, CH_2), 2.90 (d, *J* = 14 Hz, 1H, CH_2).

^{13}C-NMR ($CDCl_3$, 100 MHz): δ = 167.3 (C=O), 158.8 (C_{Ar}), 146.3 (C_{Ar}), 134.8 (C_{Ar}), 131.8 (CH_{Ar}), 131.2 (C_{Ar}), 130.9 (C_{Ar}), 130.5 ($2CH_{Ar}$), 130.0 ($2CH_{Ar}$), 129.6 (CH_{Ar}), 128.0 ($2CH_{Ar}$), 127.1 (CH_{Ar}), 123.3 (CH_{Ar}), 123.2 (CH_{Ar}), 113.9 ($2CH_{Ar}$), 91.6 (C), 55.3 (CH_3), 43.9 (CH_2), 42.0 (CH_2).

HR-MS (ESI): m/z $[M+H]^+$ cald for $C_{23}H_{22}NO_3^+$: 360.1594, found: 360.1595.

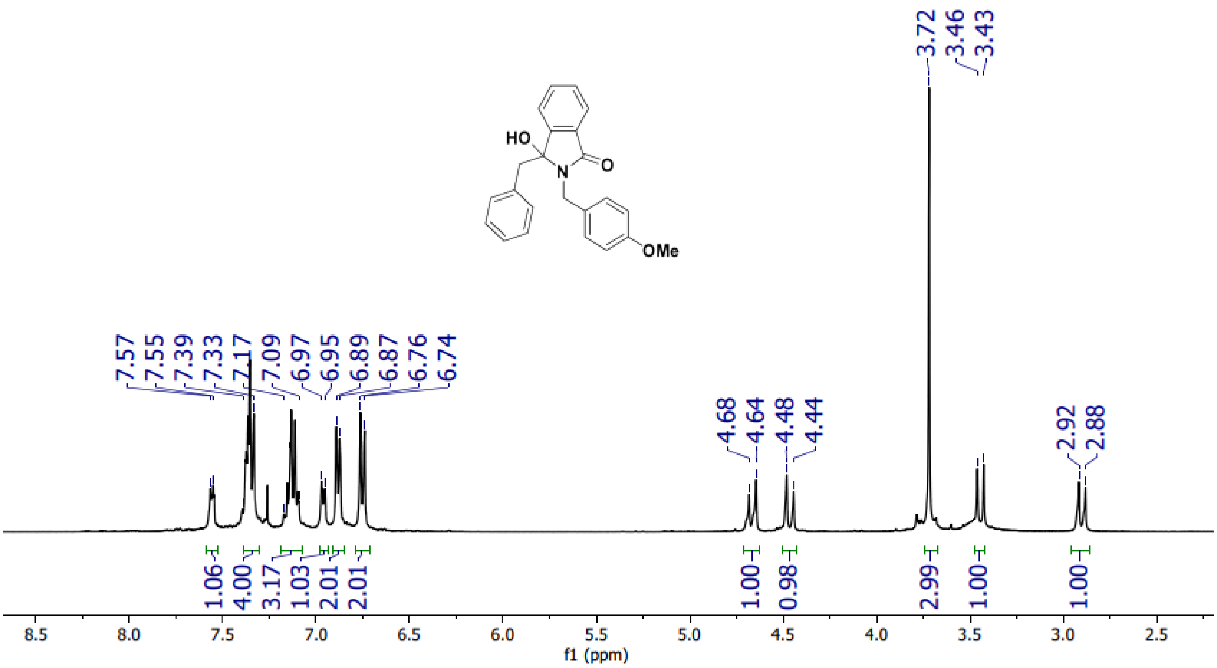

HO
N
O
OMe
3.72
3.46
3.43
7.57
7.55
7.39
7.33
7.17
7.09
6.97
6.95
6.89
6.87
6.76
6.74
4.68
4.64
4.48
4.44
2.92
2.88
1.06
4.00
3.17
1.03
2.01
2.01
1.00
0.98
2.99
1.00
1.00
8.5 8.0 7.5 7.0 6.5 6.0 5.5 5.0 4.5 4.0 3.5 3.0 2.5
f1 (ppm)

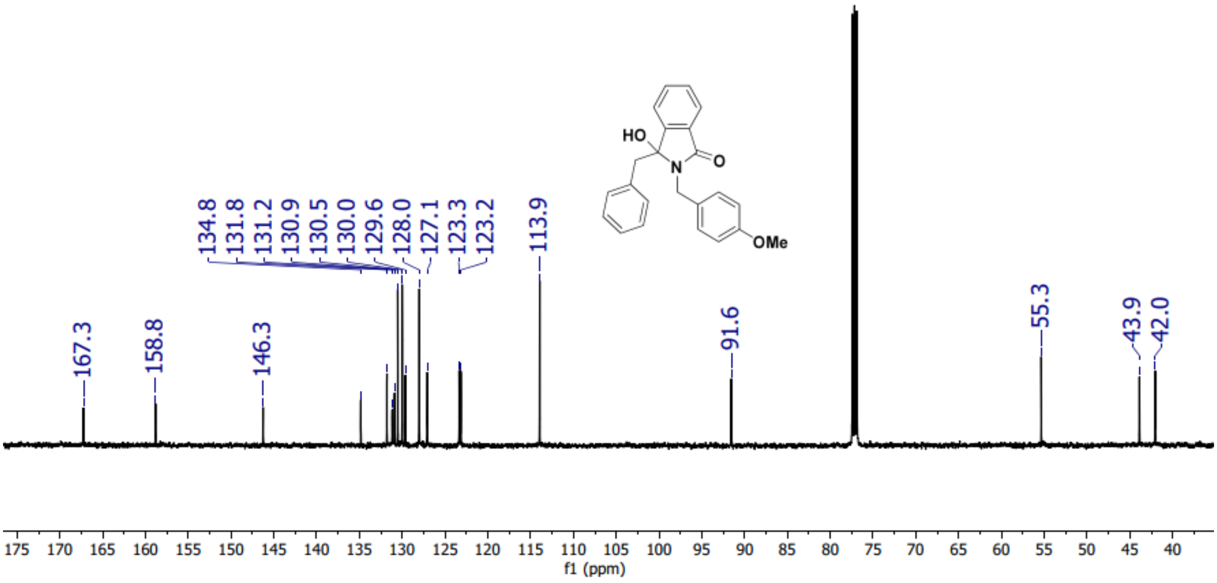

HO
N
O
OMe
134.8
131.8
131.2
130.9
130.5
130.0
129.6
128.0
127.1
123.3
123.2
113.9
167.3
158.8
146.3
91.6
55.3
43.9
42.0
175 170 165 160 155 150 145 140 135 130 125 120 115 110 105 100 95 90 85 80 75 70 65 60 55 50 45 40
f1 (ppm)

3-Benzyl-3-hydroxy-2-(4-methylbenzyl)isoindolin-1-one (1l)

Following the procedure for the synthesis of 3-hydroxyisoindolin-1-ones **1** using (*Z*)-3-benzylideneisobenzofuran-1(3*H*)-one **2a** (111 mg, 0.5 mmol, 1 equiv.) and 4-methylbenzylamine (0.127 mL, 1 mmol, 2 equiv.) with purification using column chromatography (*n*-hexane/ethyl acetate, 50:50), afforded the desired compound **1l.**

White solid; yield: 149 mg (87%); mp: 149-152°C.

IR (KBr): 3332, 2924, 2854, 1681, 1404, 1072, 702 cm^{-1}.

^{1}H-NMR ($CDCl_3$, 500 MHz): δ = 7.60 (m, 1H, CH_{Ar}), 7.37 (m, 2H, CH_{Ar}), 7.31 (d, *J* = 7.5 Hz, 2H, CH_{Ar}), 7.18-7.11 (m, 3H, CH_{Ar}), 7.07 (d, *J* = 7.5 Hz, 2H, CH_{Ar}), 6.95-6.93 (m, 1H, CH_{Ar}), 6.90 (d, *J* = 7.5 Hz, 2H, CH_{Ar}), 4.70 (d, *J* = 15 Hz, 1H, CH_2), 4.49 (d, *J* = 15 Hz, 1H, CH_2), 3.43 (d, *J* = 14 Hz, 1H, CH_2), 3.27 (br s, 1H, OH), 2.88 (d, *J* = 14 Hz, 1H, CH_2), 2.29 (s, 3H, CH_3).

^{13}C-NMR ($CDCl_3$, 125 MHz): δ = 167.4 (C=O), 146.3 (C_{Ar}), 137.0 (C_{Ar}), 135.6 (C_{Ar}), 134.9 (C_{Ar}), 131.8 (CH_{Ar}), 131.1 (C_{Ar}), 130.6 ($2CH_{Ar}$), 129.6 (CH_{Ar}), 129.3 ($2CH_{Ar}$), 128.5 ($2CH_{Ar}$), 128.0 ($2CH_{Ar}$), 127.0 (CH_{Ar}), 123.3 (CH_{Ar}), 123.2 (CH_{Ar}), 91.5 (C), 44.0 (CH_2), 42.3 (CH_2), 21.2 (CH_3).

HR-MS (ESI): m/z $[M+Na]^+$ cald for $C_{23}H_{21}NO_2Na^+$: 366.1465, found: 366.1465.

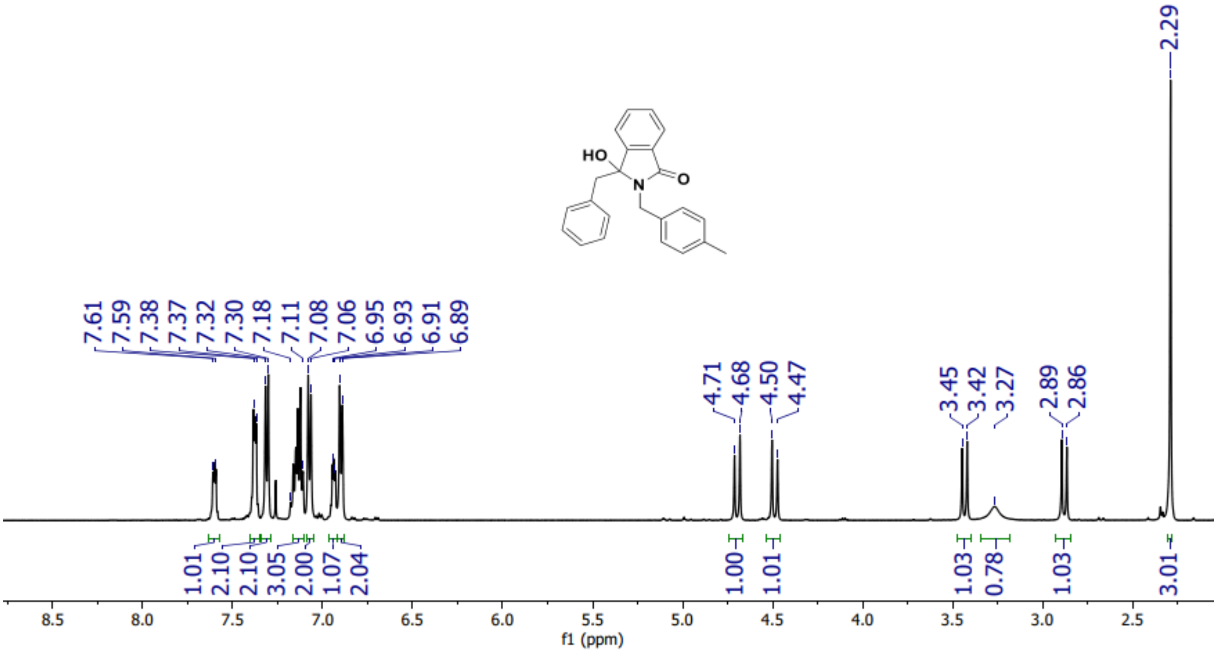
HO
N
O
7.61 7.59 7.38 7.37 7.32 7.30 7.18 7.11 7.08 7.06 6.95 6.93 6.91 6.89
4.71 4.68 4.50 4.47
3.45 3.42 3.27
2.89 2.86
2.29
1.01 2.10 2.10 3.05 2.00 1.07 2.04
1.00 1.01
1.03 0.78
1.03
3.01
f1 (ppm)

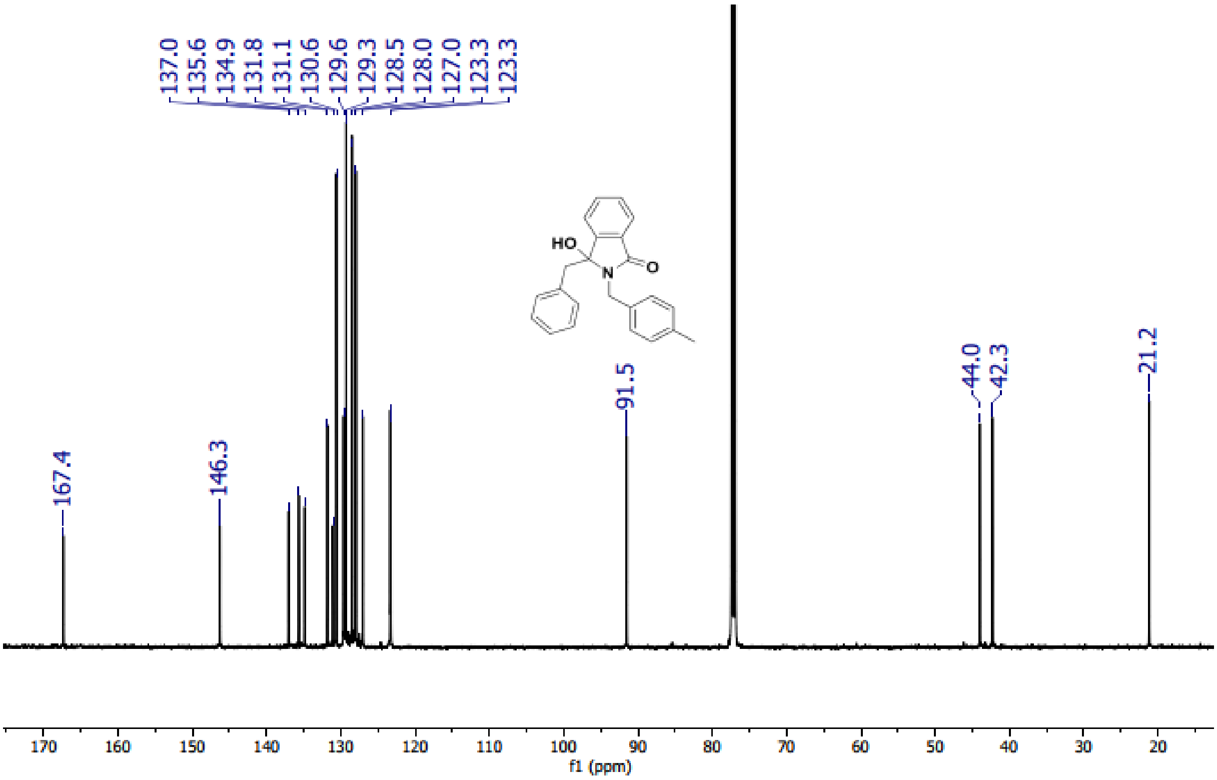
HO
N
O
137.0 135.6 134.9 131.8 131.1 130.6 129.6 129.3 128.5 128.0 127.0 123.3 123.3
167.4
146.3
91.5
44.0 42.3
21.2
f1 (ppm)

3-Benzyl-2-(4-chlorobenzyl)-3-hydroxyisoindolin-1-one (1m)

Following the procedure for the synthesis of 3-hydroxyisoindolin-1-ones **1** using (*Z*)-3-benzylideneisobenzofuran-1(3*H*)-one **2a** (111 mg, 0.5 mmol, 1 equiv.) and 4-chlorobenzylamine (0.122 mL, 1 mmol, 2 equiv.) with purification using column chromatography (*n*-hexane/ethyl acetate, 50:50), afforded the desired compound **1m.**

White solid; yield: 181 mg (78%); mp: 191-195°C.

IR (KBr): 3373, 2924, 1679, 1404, 1072, 879, 702 cm^{-1}.

^{1}H-NMR ($CDCl_3$, 500 MHz): δ = 7.64-7.62 (m, 1H, CH_{Ar}), 7.42-7.41 (m, 2H, CH_{Ar}), 7.36 (d, *J* = 8 Hz, 2H, CH_{Ar}), 7.23 (d, *J* = 8 Hz, 2H, CH_{Ar}), 7.19-7.12 (m, 3H, CH_{Ar}), 7.02-7.00 (m, 1H, CH_{Ar}), 6.88 (d, *J* = 8 Hz, 2H, CH_{Ar}), 4.75 (d, *J* = 15 Hz, 1H, CH_2), 4.52 (d, *J* = 15 Hz, 1H, CH_2), 3.45 (d, *J* = 14 Hz, 1H, CH_2), 2.95 (d, *J* = 14 Hz, 1H, CH_2).

^{13}C-NMR ($CDCl_3$, 125 MHz): δ = 167.3 (C=O), 146.2 (C_{Ar}), 137.2 (C_{Ar}), 134.6 (C_{Ar}), 133.2 (C_{Ar}), 132.1 (CH_{Ar}), 131.0 (C_{Ar}), 130.5 ($2CH_{Ar}$), 130.0 ($2CH_{Ar}$), 129.8 (CH_{Ar}), 128.8 ($2CH_{Ar}$), 128.1 ($2CH_{Ar}$), 127.2 (CH_{Ar}), 123.5 (CH_{Ar}), 123.2 (CH_{Ar}), 91.5 (CH_{Ar}), 43.9 (CH_2), 42.0 (CH_2).

HRMS (ESI): m/z $[M+Na]^+$ cald for $C_{22}H_{18}NO_2ClNa^+$: 386.0918, found: 386.0912.

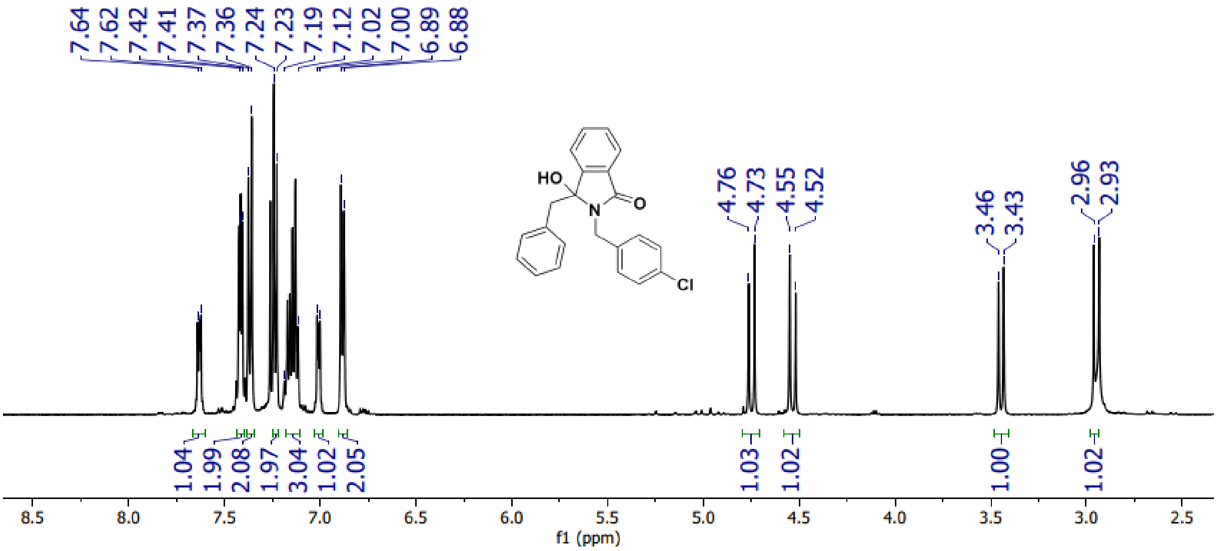
7.64 7.62 7.42 7.41 7.37 7.36 7.24 7.23 7.19 7.12 7.02 7.00 6.89 6.88
4.76 4.73 4.55 4.52
3.46 3.43
2.96 2.93
1.04 1.99 2.08 1.97 3.04 1.02 2.05 1.03 1.02 1.00 1.02
HO
N
O
Cl
8.5 8.0 7.5 7.0 6.5 6.0 5.5 5.0 4.5 4.0 3.5 3.0 2.5
f1 (ppm)

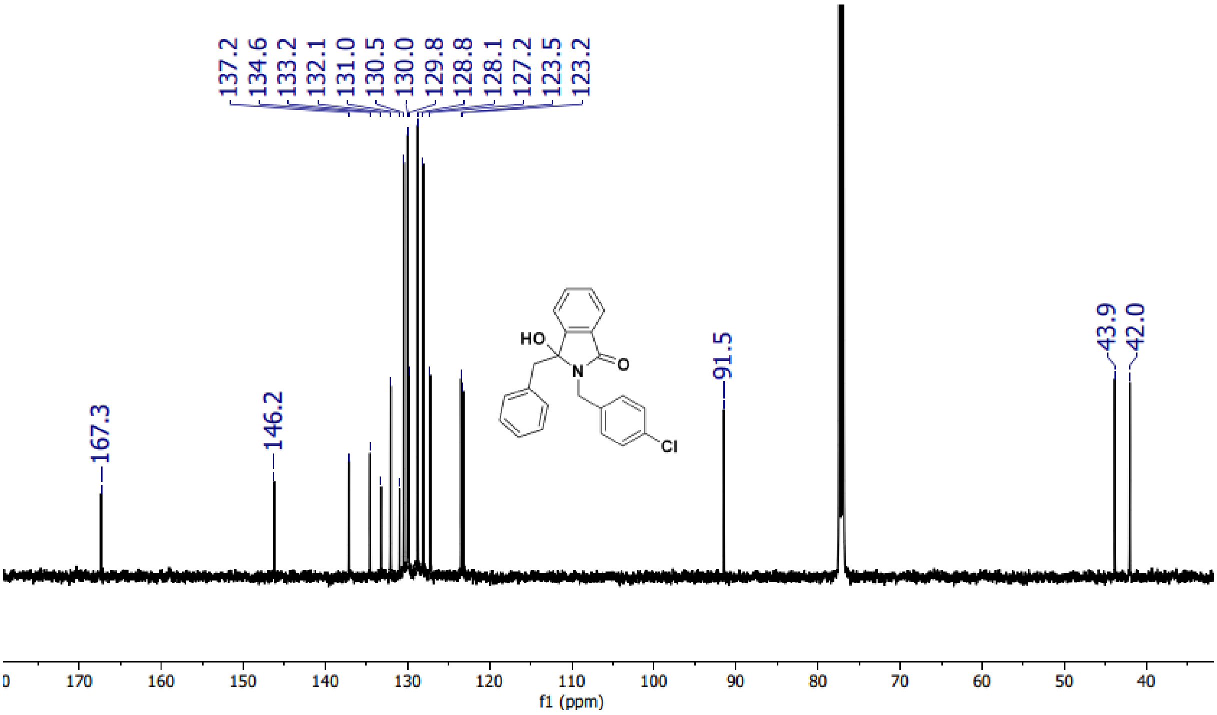
137.2 134.6 133.2 132.1 131.0 130.5 130.0 129.8 128.8 128.1 127.2 123.5 123.2
167.3
146.2
91.5
43.9 42.0
HO
N
O
Cl
170 160 150 140 130 120 110 100 90 80 70 60 50 40
f1 (ppm)

3-Benzyl-2-(4-fluorobenzyl)-3-hydroxyisoindolin-1-one (1n)

Following the procedure for the synthesis of 3-hydroxyisoindolin-1-ones **1** using (*Z*)-3-benzylideneisobenzofuran-1(3*H*)-one **2a** (111 mg, 0.5 mmol, 1 equiv.) and 4-fluorobenzylamine (0.114 mL, 1 mmol, 2 equiv.) with purification using column chromatography (*n*-hexane/ethyl acetate, 50:50), afforded the desired compound **1n.**

Light yellow solid; yield: 156 mg (90%); mp: 197-200°C.

IR (KBr): 3279, 3032, 2924, 1682, 1512, 1404, 1219, 1072, 764, 702 cm^{-1}.

^{1}H-NMR ($CDCl_3$, 500 MHz): δ = 7.63-7.62 (m, 1H, CH_{Ar}), 7.43-7.40 (m, 4H, CH_{Ar}), 7.18-7.11 (m, 3H, CH_{Ar}), 7.02-7.00 (m, 1H, CH_{Ar}), 6.95 (t, *J* = 8.5 Hz, 2H, CH_{Ar}), 6.88 (d, *J* = 8.5 Hz, 2H, CH_{Ar}), 4.75 (d, *J* = 15 Hz, 1H, CH_2), 4.54 (d, *J* = 15 Hz, 1H, CH_2), 3.45 (d, *J* = 14 Hz, 1H, CH_2), 2.95 (d, *J* = 14 Hz, 1H, CH_2), 2.94 (br s, 1H, OH).

^{13}C-NMR ($CDCl_3$, 125 MHz): δ = 167.3 (C=O), 162.7 (d, *J* = 245 Hz, C_{Ar}), 146.2 (C_{Ar}), 134.6 (C_{Ar}), 134.4 (d, *J* = 2.5 Hz, C_{Ar}), 132.0 (CH_{Ar}), 131.0 (C_{Ar}), 130.5 (2CH_{Ar}), 130.3 (2CH_{Ar}, d, *J* = 7.5 Hz, CH_{Ar}), 129.8 (CH_{Ar}), 128.1 (2CH_{Ar}), 127.2 (CH_{Ar}), 123.4 (CH_{Ar}), 123.2 (CH_{Ar}), 115.4 (2CH_{Ar}, d, *J* = 21.3 Hz, CH_{Ar}), 91.5 (C), 43.9 (CH_2), 42.0 (CH_2).

HR-MS (ESI): m/z $[M+Na]^+$ cald for $C_{22}H_{18}NO_2FNa^+$: 370.1214, found: 370.1213.

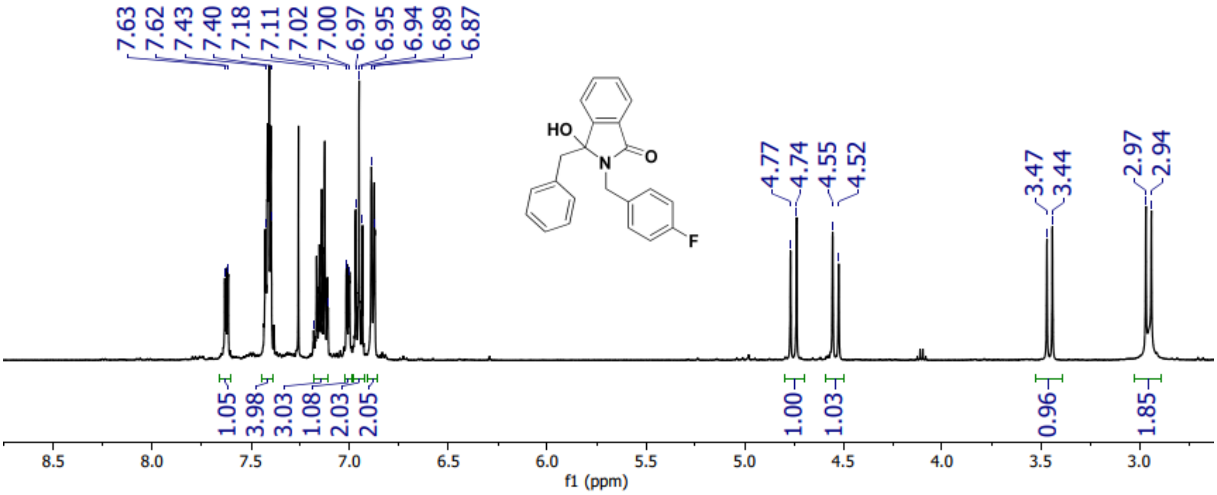
7.63
7.62
7.43
7.40
7.18
7.11
7.02
7.00
6.97
6.95
6.94
6.89
6.87
HO
N
O
F
4.77
4.74
4.55
4.52
3.47
3.44
2.97
2.94
1.05
3.98
3.03
1.08
2.03
2.05
1.00
1.03
0.96
1.85
8.5
8.0
7.5
7.0
6.5
6.0
5.5
5.0
4.5
4.0
3.5
3.0
f1 (ppm)

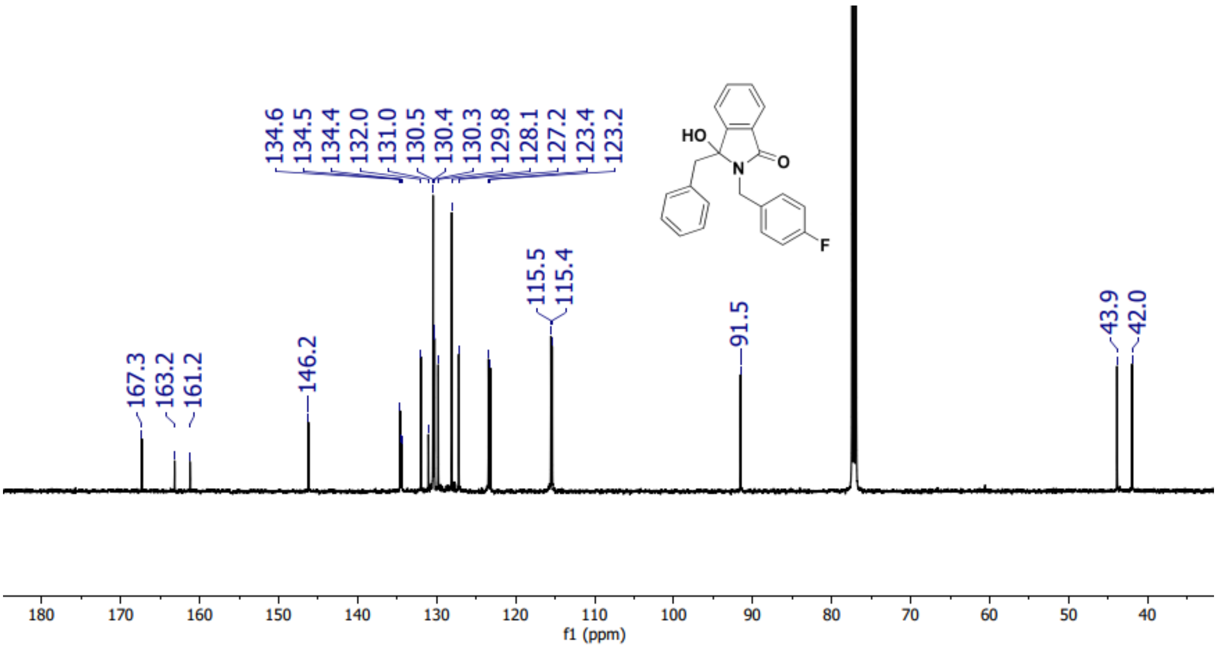
134.6
134.5
134.4
132.0
131.0
130.5
130.4
130.3
129.8
128.1
127.2
123.4
123.2
HO
N
O
F
115.5
115.4
91.5
43.9
42.0
167.3
163.2
161.2
146.2
180
170
160
150
140
130
120
110
100
90
80
70
60
50
40
f1 (ppm)

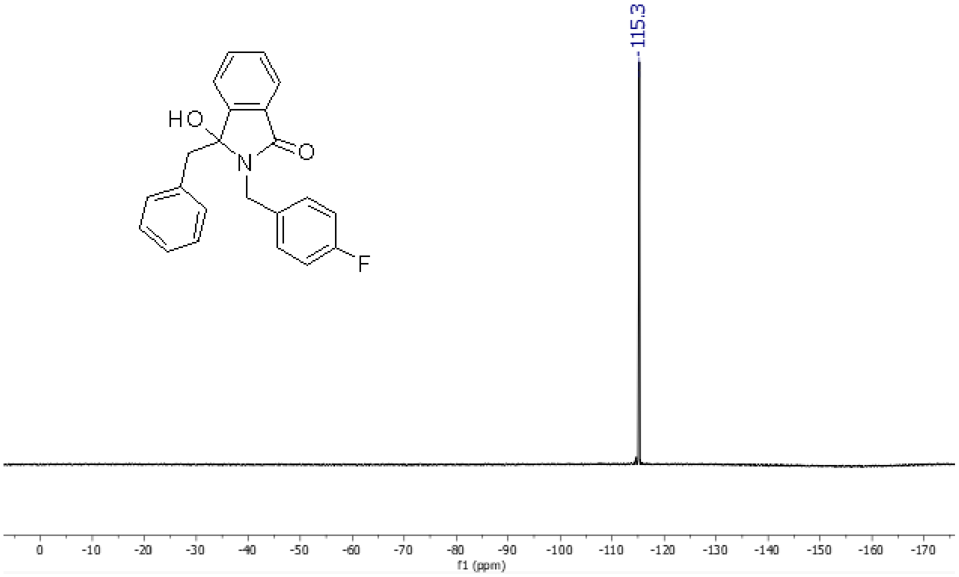

-115.3
HO
N
O
F
0 -10 -20 -30 -40 -50 -60 -70 -80 -90 -100 -110 -120 -130 -140 -150 -160 -170
f1 (ppm)

3-Benzyl-2-(3-fluorobenzyl)-3-hydroxyisoindolin-1-one (1o)

Following the procedure for the synthesis of 3-hydroxyisoindolin-1-ones **1** using (*Z*)-3-benzylideneisobenzofuran-1(3*H*)-one **2a** (111 mg, 0.5 mmol, 1 equiv.) and 3-fluorobenzylamine (0.114 mL, 1 mmol, 2 equiv.) with purification using column chromatography (*n*-hexane/ethyl acetate, 50:50), afforded the desired compound **1o.**

Light yellow solid; yield: 144 mg (83%); mp: 168-173°C.

IR (KBr): 3271, 3032, 2924, 1690, 1589, 1396, 1072, 702 cm^{-1}.

^{1}H-NMR ($CDCl_3$, 500 MHz): δ = 7.63-7.61 (m, 1H, CH_{Ar}), 7.43-7.39 (m, 2H, CH_{Ar}), 7.26-7.21 (m, 1H, CH_{Ar}), 7.18-7.09 (m, 5H, CH_{Ar}), 6.98-6.97 (m, 1H, CH_{Ar}), 6.93-6.89 (m, 3H, CH_{Ar}), 4.72 (d, *J* = 15 Hz, 1H, CH_2), 4.52 (d, *J* = 15 Hz, 1H, CH_2), 3.44 (d, *J* = 14 Hz, 1H, CH_2), 3.19 (br s, 1H, OH) 2.91 (d, *J* = 14 Hz, 1H, CH_2).

^{13}C-NMR ($CDCl_3$, 125 MHz): δ = 167.3 (C=O), 163.0 (d, *J* = 245 Hz, C_{Ar}), 146.2 (C_{Ar}), 141.3 (d, *J* = 7.5 Hz, C_{Ar}), 134.6 (C_{Ar}), 132.1 (CH_{Ar}), 130.9 (C_{Ar}), 130.5 (2CH_{Ar}), 130.1 (d, *J* = 8.8 Hz, CH_{Ar}), 129.8 (CH_{Ar}), 128.1 (2CH_{Ar}), 127.2 (CH_{Ar}), 123.9 (d, *J* = 2.5 Hz, CH_{Ar}), 123.5 (CH_{Ar}), 123.3 (CH_{Ar}), 115.3 (d, *J* = 22.5 Hz, CH_{Ar}), 114.4 (d, *J* = 21.3 Hz, CH_{Ar}), 91.5 (C), 43.9 (CH_2), 42.1 (CH_2).

HR-MS (ESI): m/z $[M+Na]^+$ cald for $C_{22}H_{18}NO_2FNa^+$: 370.1214, found: 370.1214.

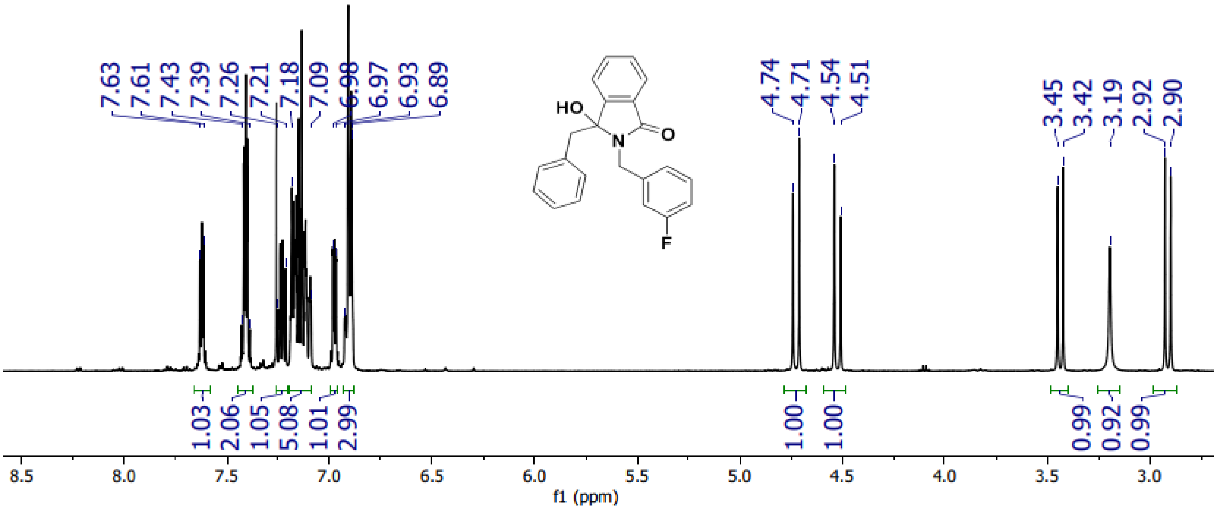
7.63
7.61
7.43
7.39
7.26
7.21
7.18
7.09
6.98
6.97
6.93
6.89
HO
N
O
F
4.74
4.71
4.54
4.51
3.45
3.42
3.19
2.92
2.90
1.03
2.06
1.05
5.08
1.01
2.99
1.00
1.00
0.99
0.92
0.99
8.5
8.0
7.5
7.0
6.5
6.0
5.5
5.0
4.5
4.0
3.5
3.0
f1 (ppm)

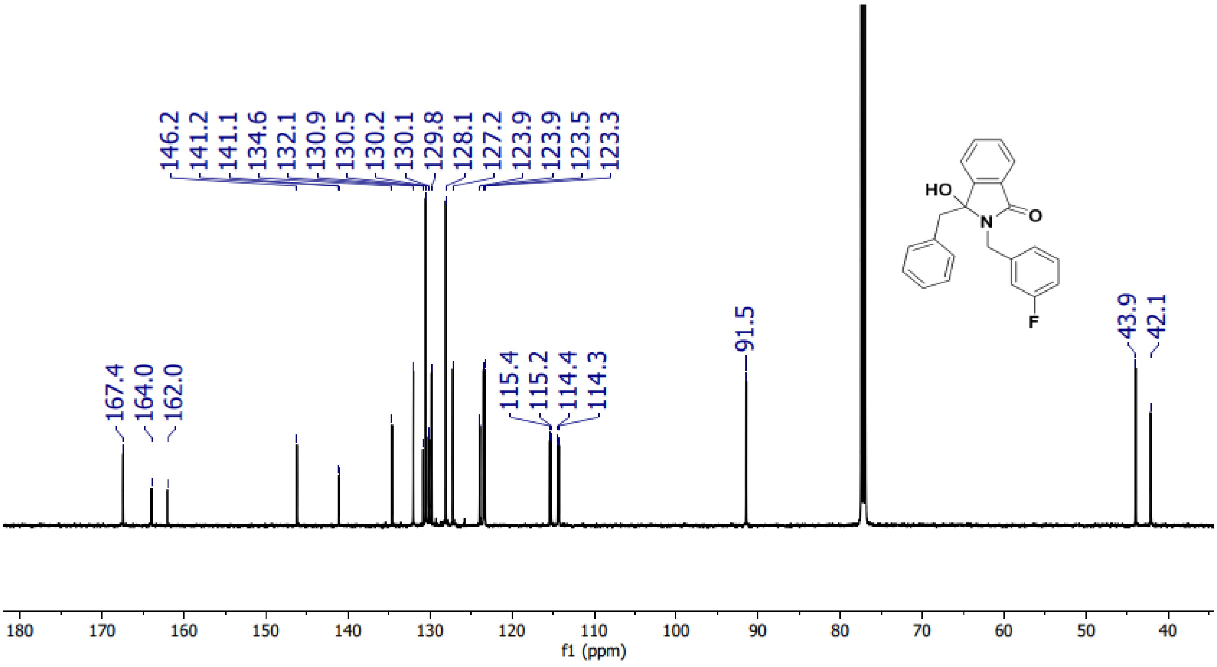
146.2
141.2
141.1
134.6
132.1
130.9
130.5
130.2
130.1
129.8
128.1
127.2
123.9
123.9
123.5
123.3
HO
N
O
F
167.4
164.0
162.0
115.4
115.2
114.4
114.3
91.5
43.9
42.1
180
170
160
150
140
130
120
110
100
90
80
70
60
50
40
f1 (ppm)

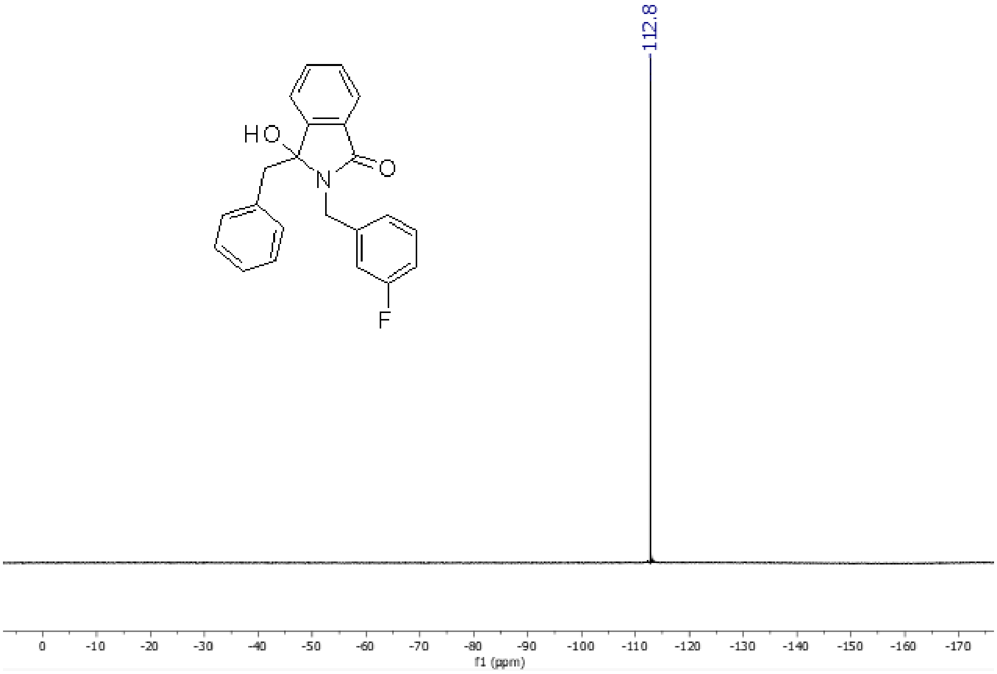
112.8
HO
N
O
F
0 -10 -20 -30 -40 -50 -60 -70 -80 -90 -100 -110 -120 -130 -140 -150 -160 -170
f1 (ppm)

3-Benzyl-3-hydroxy-2-(thiophen-2-ylmethyl)isoindolin-1-one (1p)

Following the procedure for the synthesis of 3-hydroxyisoindolin-1-ones **1** using (*Z*)-3-benzylideneisobenzofuran-1(3*H*)-one **2a** (111 mg, 0.5 mmol, 1 equiv.) and 2-thiophenemethylamine (0.103 mL, 1 mmol, 2 equiv.) with purification using column chromatography (*n*-hexane/ethyl acetate, 60:40), afforded the desired compound **1p.**

Light yellow solid; yield: 150 mg (90%); mp: 185-189°C.

IR (KBr): 3348, 3032, 2924, 1682, 1420, 1080, 694, 570 cm^{-1}.

^{1}H-NMR ($CDCl_3$, 500 MHz): δ = 7.60 (d, *J* = 7 Hz, 1H, CH_{Ar}), 7.42-7.37 (m, 2H, CH_{Ar}), 7.17-7.10 (m, 5H, CH_{Ar}), 7.01 (d, *J* = 7 Hz, 1H, CH_{Ar}), 6.90-6.89 (m, 3H, CH_{Ar}), 4.92 (d, *J* = 15.5 Hz, 1H, CH_2), 4.63 (d, *J* = 15.5 Hz, 1H, CH_2), 3.53 (d, *J* = 14 Hz, 1H, CH_2), 3.26 (br s, 1H, OH) 3.00 (d, *J* = 14 Hz, 1H, CH_2).

^{13}C-NMR ($CDCl_3$, 125 MHz): δ = 167.0 (C=O), 146.2 (C_{Ar}), 140.9 (C_{Ar}), 134.7 (C_{Ar}), 132.0 (CH_{Ar}), 130.9 (C_{Ar}), 130.5 ($2CH_{Ar}$), 129.7 (CH_{Ar}), 128.0 ($2CH_{Ar}$), 127.2 (CH_{Ar}), 127.1 (CH_{Ar}), 126.8 (CH_{Ar}), 125.5 (CH_{Ar}), 123.4 (CH_{Ar}), 123.3 (CH_{Ar}), 91.4 (C), 43.7 (CH_2), 37.1 (CH_2).

HR-MS (ESI): m/z $[M+Na]^+$ cald for $C_{20}H_{17}NO_2SNa^+$: 358.0872, found: 358.0871.

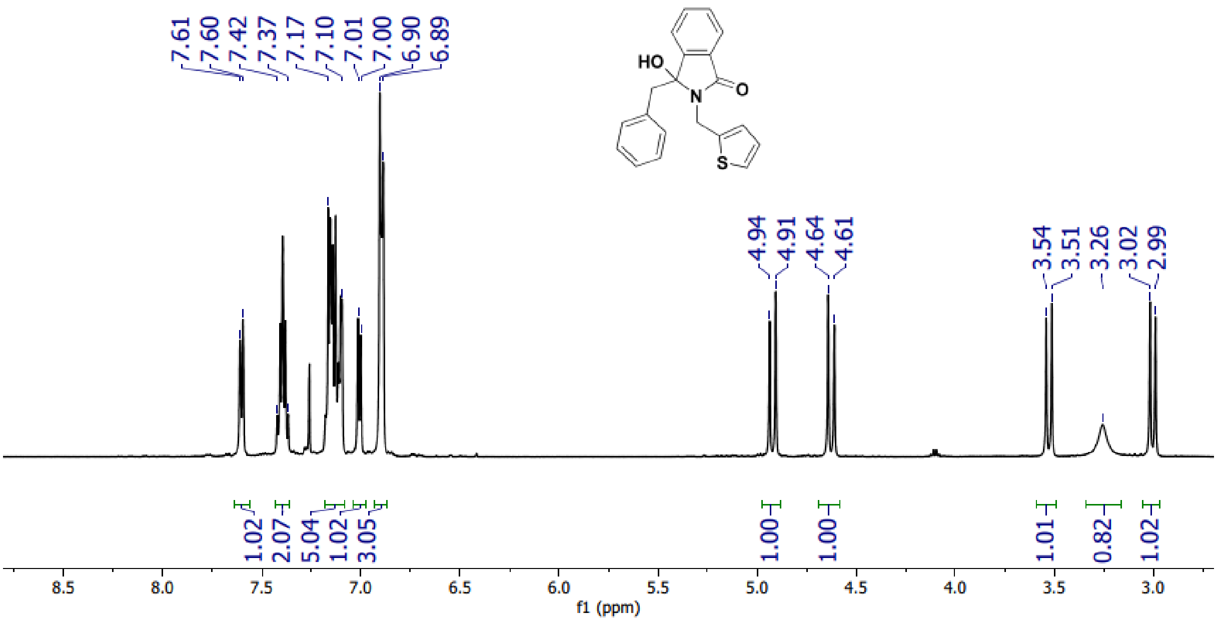
7.61
7.60
7.42
7.37
7.17
7.10
7.01
7.00
6.90
6.89
HO
N
O
S
4.94
4.91
4.64
4.61
3.54
3.51
3.26
3.02
2.99
1.02
2.07
5.04
1.02
3.05
1.00
1.00
1.01
0.82
1.02
f1 (ppm)

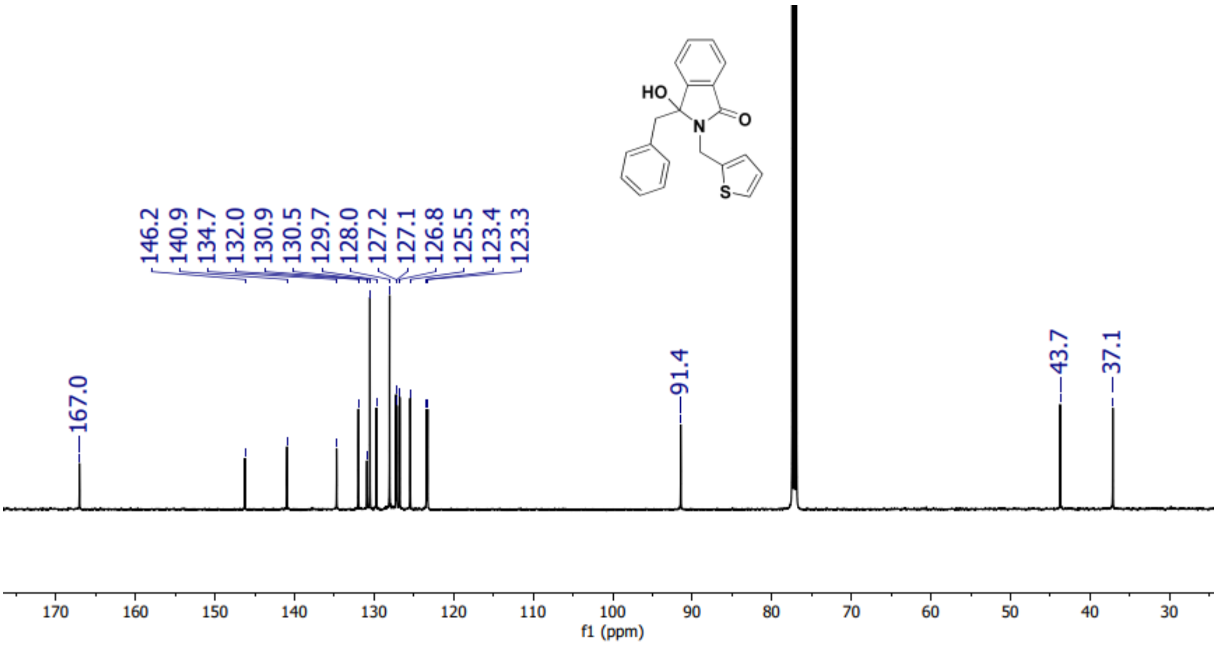
HO
N
O
S
146.2
140.9
134.7
132.0
130.9
130.5
129.7
128.0
127.2
127.1
126.8
125.5
123.4
123.3
167.0
91.4
43.7
37.1
f1 (ppm)

3-Benzyl-2-(furan-2-ylmethyl)-3-hydroxyisoindolin-1-one (1q)

Following the procedure for the synthesis of 3-hydroxyisoindolin-1-ones using (*Z*)-3-benzylideneisobenzofuran-1(3*H*)-one **2a** (111 mg, 0.5 mmol, 1 equiv.) and furfurylamine (0.088 mL, 1 mmol, 2 equiv.) with purification using column chromatography (*n*-hexane/ethyl acetate, 60:40), afforded the desired compound **1q.**

Light brown solid; yield: 100 mg (63%); mp: 174-177°C.

IR (KBr): 3333, 3279, 3032, 2932, 1682, 1466, 1420, 1211, 1103, 756, 710 cm^{-1}.

^{1}H-NMR ($CDCl_3$, 500 MHz): δ = 7.64 (d, *J* = 7 Hz, 1H, CH_{Ar}), 7.43 (td, *J* = 7 and 1 Hz, 1H, CH_{Ar}), 7.38 (td, *J* = 7 and 1 Hz, 1H, CH_{Ar}), 7.31 (dd, *J* = 2 and 1 Hz, 1 H, CH_{Ar}), 7.18-7.11 (m, 3H, CH_{Ar}), 7.08 (d, *J* = 7 Hz, 1H, CH_{Ar}), 6.91-6.89 (m, 2H, CH_{Ar}), 6.37-6.36 (m, 1H, CH_{Ar}), 6.31 (dd, *J* = 3.5 and 1 Hz, 1 H, CH_{Ar}), 4.81 (d, *J* = 16 Hz, 1H, CH_2), 4.54 (d, *J* = 16 Hz, 1H, CH_2), 3.55 (d, *J* = 14 Hz, 1H, CH_2), 3.06 (d, *J* = 14 Hz, 1H, CH_2) , 3.02 (br s, 1H, OH).

^{13}C-NMR ($CDCl_3$, 125 MHz): δ = 167.0 (C=O), 151.1 (C_{Ar}), 146.3 (C_{Ar}), 141.9 (CH_{Ar}), 134.8 (C_{Ar}), 132.0 (CH_{Ar}), 130.9 (C_{Ar}), 130.5 ($2CH_{Ar}$), 129.7 (CH_{Ar}), 128.1 ($2CH_{Ar}$), 127.1 (CH_{Ar}), 123.4 (CH_{Ar}), 123.2 (CH_{Ar}), 110.8 (CH_{Ar}), 108.6 (CH_{Ar}), 91.1 (C), 43.5 (CH_2), 35.3 (CH_2).

HR-MS (ESI): m/z $[M+Na]^+$ cald for $C_{20}H_{17}NO_3Na^+$: 342.1101, found: 342.1099.

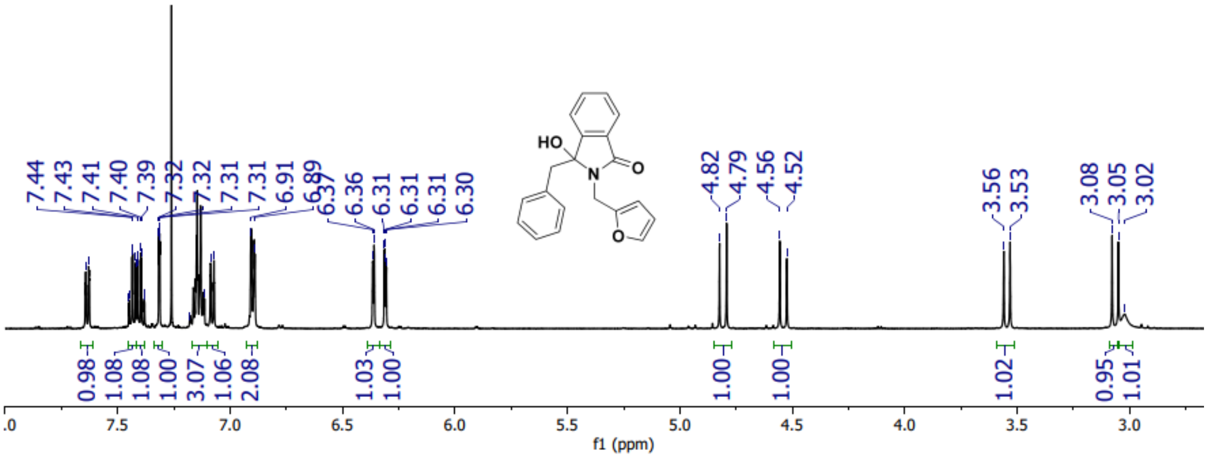
7.44
7.43
7.41
7.40
7.39
7.32
7.32
7.31
7.31
6.91
6.89
6.37
6.36
6.31
6.31
6.31
6.30
4.82
4.79
4.56
4.52
3.56
3.53
3.08
3.05
3.02
HO
N
O
O
0.98
1.08
1.08
1.00
3.07
1.06
2.08
1.03
1.00
1.00
1.00
1.02
0.95
1.01
f1 (ppm)

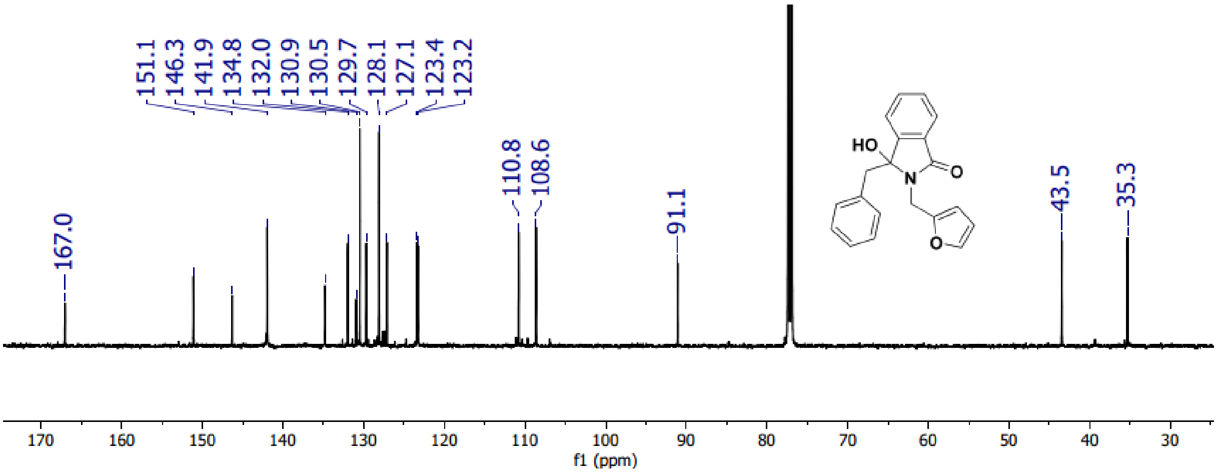
151.1
146.3
141.9
134.8
132.0
130.9
130.5
129.7
128.1
127.1
123.4
123.2
167.0
110.8
108.6
91.1
43.5
35.3
HO
N
O
O
f1 (ppm)

3-Benzyl-3-hydroxy-2-(pyridin-4-ylmethyl)isoindolin-1-one (1r)

Following the procedure for the synthesis of 3-hydroxyisoindolin-1-ones **1** using (*Z*)-3-benzylideneisobenzofuran-1(3*H*)-one **2a** (111 mg, 0.5 mmol, 1 equiv.) and 4-picolylamine (0.101 mL, 1 mmol, 2 equiv.) with purification using column chromatography (*n*-hexane/ethyl acetate, 30:70), afforded the desired compound **1r.**

Light brown solid; yield: 117 mg (71%); mp: 188-192°C.

IR (KBr): 3294, 3032, 2924, 1690, 1412, 1072, 772, 702 cm^{-1}.

^{1}H-NMR ($CDCl_3$, 500 MHz): δ = 7.71 (d, *J* = 6 Hz, 2H, CH_{Ar}), 7.56 (d, *J* = 7.5 Hz, 1H, CH_{Ar}), 7.46 (t, *J* = 7.5 Hz, 1H, CH_{Ar}), 7.38 (t, *J* = 7.5 Hz, 1H, CH_{Ar}), 7.23 (d, *J* = 7.5 Hz, 1H, CH_{Ar}), 7.16 (d, *J* = 6 Hz, 2H, CH_{Ar}), 7.13-7.08 (m, 3H, CH_{Ar}), 6.84 (d, *J* = 7.5 Hz, 2H, CH_{Ar}), 4.80 (d, *J* = 15.5 Hz, 1H, CH_2), 4.37 (d, *J* = 15.5 Hz, 1H, CH_2), 3.44 (d, *J* = 14 Hz, 1H, CH_2), 3.14 (d, *J* = 14 Hz, 1H, CH_2).

^{13}C-NMR ($CDCl_3$, 125 MHz): δ = 167.2 (C=O), 148.4 ($2CH_{Ar}$), 148.1 (C_{Ar}), 147.0 (C_{Ar}), 134.6 (C_{Ar}), 132.2 (CH_{Ar}), 131.0 (C_{Ar}), 130.2 ($2CH_{Ar}$), 129.6 (CH_{Ar}), 128.1 ($2CH_{Ar}$), 127.1 (CH_{Ar}), 123.9 ($2CH_{Ar}$), 123.3 (CH_{Ar}), 123.0 (CH_{Ar}), 91.6 (C), 44.1 (CH_2), 41.9 (CH_2).

HR-MS (ESI): m/z $[M+Na]^+$ cald for $C_{21}H_{18}N_2O_2Na^+$: 353.1260, found: 353.1258.

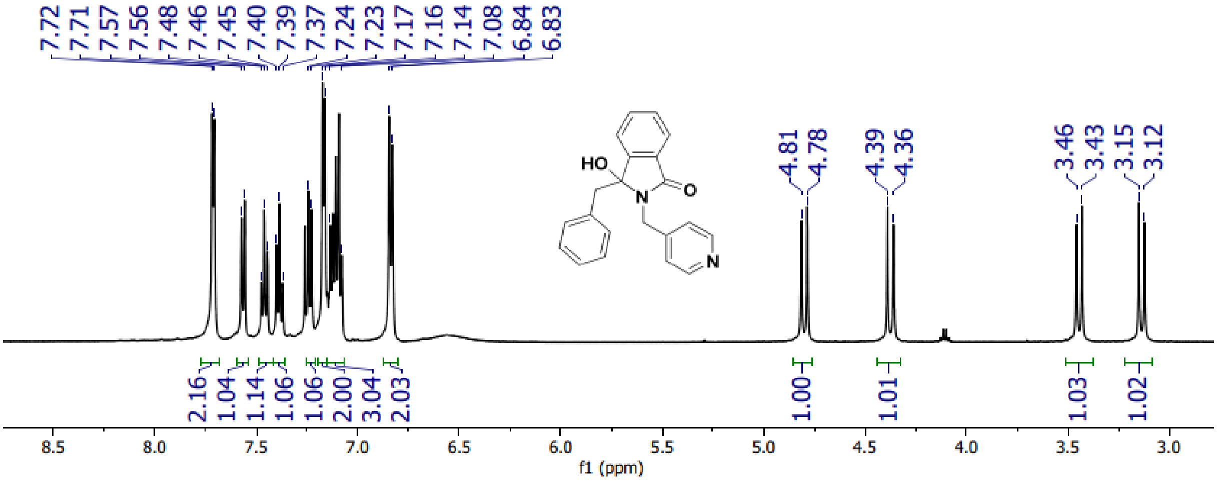
7.72
7.71
7.57
7.56
7.48
7.46
7.45
7.40
7.39
7.37
7.24
7.23
7.17
7.16
7.14
7.08
6.84
6.83
4.81
4.78
4.39
4.36
3.46
3.43
3.15
3.12
HO
N
O
N
2.16
1.04
1.14
1.06
1.06
2.00
3.04
2.03
1.00
1.01
1.03
1.02
f1 (ppm)

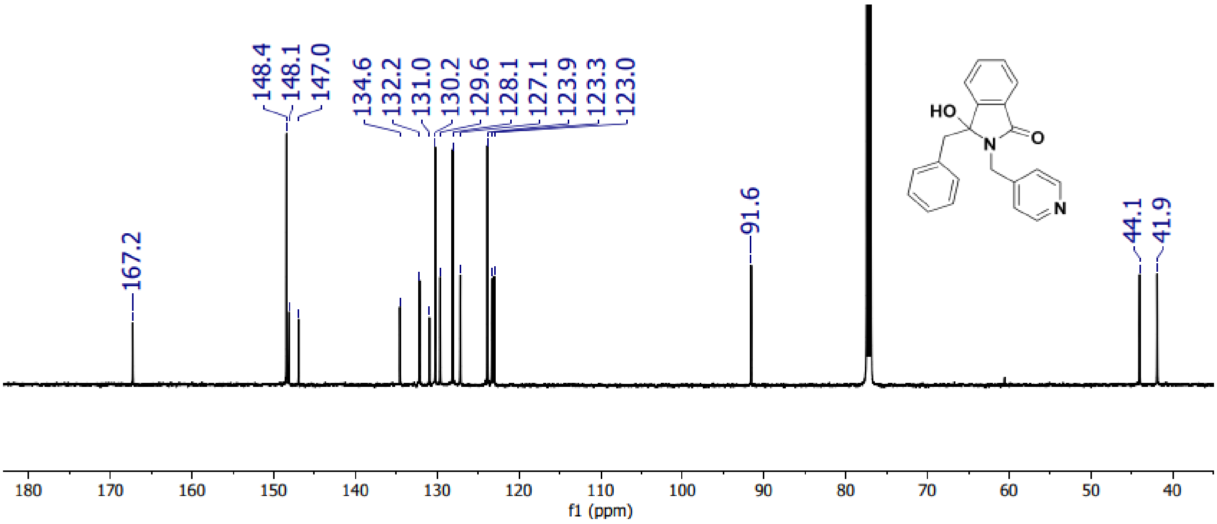
167.2
148.4
148.1
147.0
134.6
132.2
131.0
130.2
129.6
128.1
127.1
123.9
123.3
123.0
91.6
44.1
41.9
HO
N
O
N
f1 (ppm)

3-Benzyl-3-hydroxy-2-(pyridin-3-ylmethyl)isoindolin-1-one (1s)

Following the procedure for the synthesis of 3-hydroxyisoindolin-1-ones **1** using (*Z*)-3-benzylideneisobenzofuran-1(3*H*)-one **2a** (111 mg, 0.5 mmol, 1 equiv.) and 3-picolylamine (0.102 mL, 1 mmol, 2 equiv.) with purification using column chromatography (*n*-hexane/ethyl acetate, 30:70), afforded the desired compound **1s.**

Light brown solid; yield: 100 mg (61%); mp: 218-224°C.

IR (KBr): 3379, 3055, 2924, 2855, 1697, 1389, 1065, 772, 710 cm^{-1}.

^{1}H-NMR ($CDCl_3$, 500 MHz): δ = 8.40 (s, 1H, CH_{Ar}), 7.74 (d, *J* = 7.5 Hz, 1H, CH_{Ar}), 7.52 (d, *J* = 7.5 Hz, 1H, CH_{Ar}), 7.49 (td, *J* = 7.5 and 1 Hz, 1H, CH_{Ar}), 7.47-7.30 (m, 1H, CH_{Ar}), 7.37 (t, *J* = 7.5 Hz, 1H, CH_{Ar}), 7.30 (d, *J* = 7.5 Hz, 1H, CH_{Ar}), 7.14-7.09 (m, 3H), 6.95-6.93 (m, 1H, CH_{Ar}), 6.86 (d, *J* = 7.5 Hz, 2H, CH_{Ar}), 4.81 (d, *J* = 15 Hz, 1H , CH_2), 4.47 (d, *J* = 15 Hz, 1H, CH_2), 3.52 (d, *J* = 14 Hz, 1H, CH_2), 3.20 (d, *J* = 14 Hz, 1H, CH_2).

^{13}C-NMR ($CDCl_3$, 125 MHz): δ = 167.2 (C=O), 148.9 (CH_{Ar}), 147.2 (C_{Ar}), 146.9 (C_{Ar}), 137.7 (CH_{Ar}), 134.7 (CH_{Ar}), 134.3 (C_{Ar}), 132.1 (CH_{Ar}), 131.2 (C_{Ar}), 130.3 (2CH_{Ar}), 129.5 (CH_{Ar}), 128.1 (2CH_{Ar}), 127.1 (CH_{Ar}), 123.4 (CH_{Ar}), 123.2 (CH_{Ar}), 123.0 (CH_{Ar}), 91.6 (C), 44.0 (CH_2), 40.6 (CH_2).

HR-MS (ESI): m/z $[M+Na]^+$ cald for $C_{21}H_{18}N_2O_2Na^+$: 353.1260, found: 353.1260.

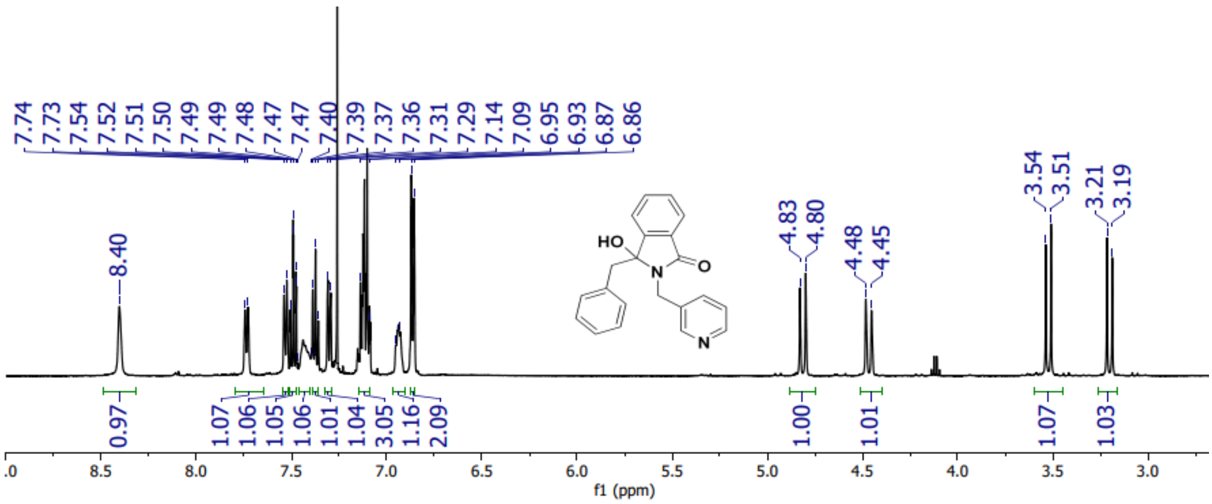
7.74
7.73
7.54
7.52
7.51
7.50
7.49
7.49
7.48
7.47
7.47
7.40
7.39
7.37
7.36
7.31
7.29
7.14
7.09
6.95
6.93
6.87
6.86
8.40
4.83
4.80
4.48
4.45
3.54
3.51
3.21
3.19
HO
N
O
N
0.97
1.07
1.06
1.05
1.06
1.01
1.04
3.05
1.16
2.09
1.00
1.01
1.07
1.03
f1 (ppm)

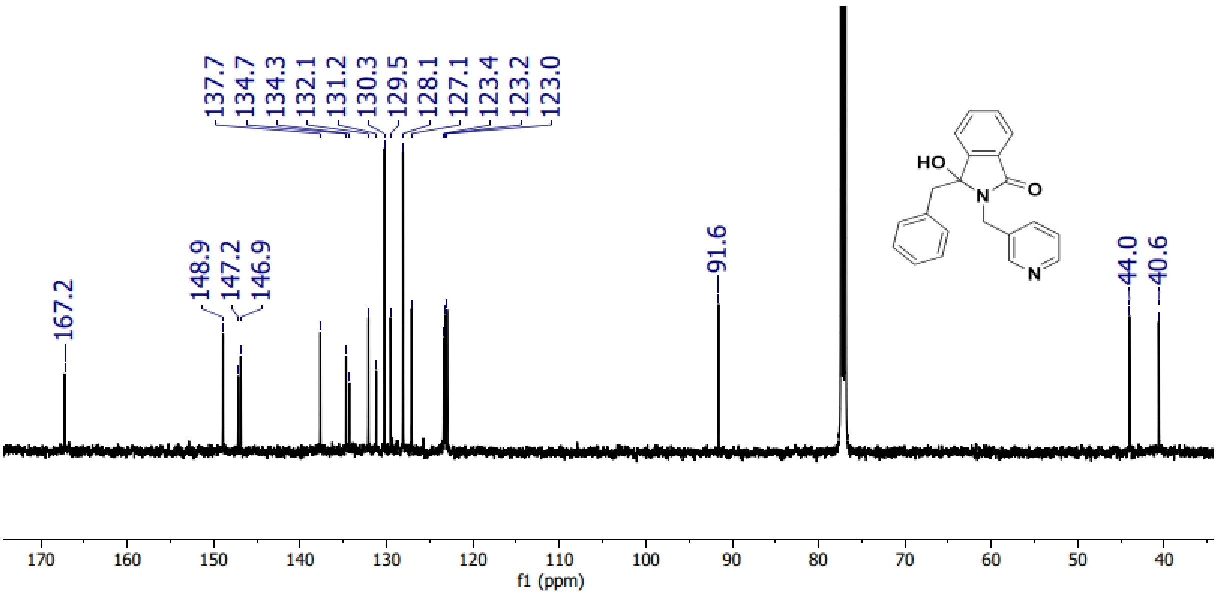
137.7
134.7
134.3
132.1
131.2
130.3
129.5
128.1
127.1
123.4
123.2
123.0
167.2
148.9
147.2
146.9
91.6
44.0
40.6
HO
N
O
N
f1 (ppm)

2-(2-(1H-indol-3-yl)ethyl)-3-benzyl-3-hydroxyisoindolin-1-one (1t)

Following the procedure for the synthesis of 3-hydroxyisoindolin-1-ones **1** using (*Z*)-3-benzylideneisobenzofuran-1(3*H*)-one **2a** (111 mg, 0.5 mmol, 1 equiv.) and tryptamine (160 mg, 1 mmol, 2 equiv.) with purification using column chromatography (*n*-hexane/ethyl acetate, 50:50), afforded the desired compound **1t.**

Light brown solid; yield: 141 mg (74%); mp: 170-173°C.

IR (KBr): 3418, 3341, 3055, 2924, 1674, 1412, 1080, 748 cm^{-1}.

^{1}H-NMR (DMSO-*d*6, 500 MHz): δ = 10.87 (br s, 1H, NH), 7.71 (d, *J* = 8 Hz, 1H, CH_{Ar}), 7.59 (t, *J* = 7.5 Hz, 1H, CH_{Ar}), 7.54 (d, *J* = 7.5 Hz, 1H, CH_{Ar}), 7.47-7.42 (m, 2H, CH_{Ar}), 7.38 (d, *J* = 8 Hz, 1H, CH_{Ar}), 7.28 (s, 1H, CH_{Ar}), 7.10 (t, *J* = 8 Hz, 1H, CH_{Ar}), 7.09-7.02 (m, 4H, CH_{Ar}), 6.80-6.79 (m, 2H, CH_{Ar}), 6.73 (br s, 1H, OH), 3.82 (td, *J* = 12 and 5.5 Hz, 1H, CH_2), 3.63 (td, *J* = 12 and 5.5 Hz, 1H, CH_2), 3.45 (d, *J* = 14 Hz, 1H, CH_2), 3.32 (d, *J* = 14 Hz, 1H, CH_2), 3.12-3.04 (m, 2H, CH_2).

^{13}C-NMR (DMSO-*d*6, 125 MHz): δ = 165.9 (C=O), 147.1 (C_{Ar}), 136.3 (CH_{Ar}), 135.2 (C_{Ar}), 131.6 (C_{Ar}), 131.4 (C_{Ar}), 129.8 (2CH_{Ar}), 129.0 (C_{Ar}), 127.5 (2CH_{Ar}), 127.2 (CH_{Ar}), 126.3 (CH_{Ar}), 122.9 (CH_{Ar}), 122.7 (CH_{Ar}), 121.8 (CH_{Ar}), 121.0 (CH_{Ar}), 118.4 (CH_{Ar}), 118.3 (CH_{Ar}), 111.9 (CH_{Ar}), 111.4 (C_{Ar}), 91.0 (C), 42.5 (CH_2), 39.8 (CH_2), 25.0 (CH_2).

HR-MS (ESI): m/z $[M+H]^+$ cald for $C_{25}H_{23}N_2O_2^+$: 383.1754, found: 383.1751.

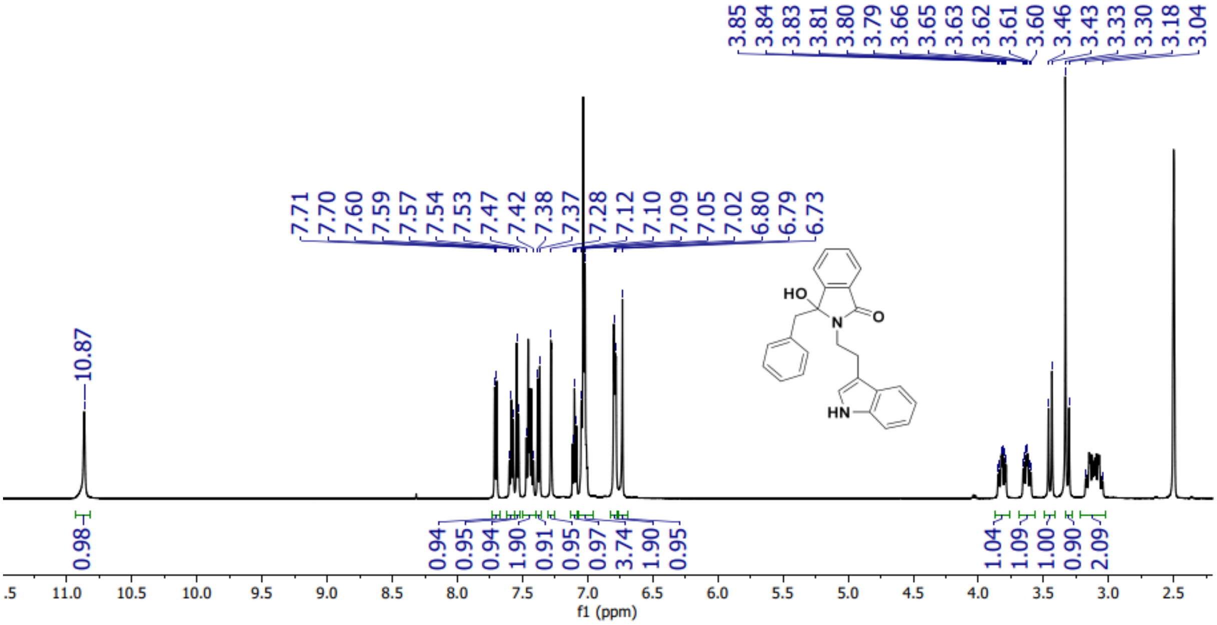
3.85 3.84 3.83 3.81 3.80 3.79 3.66 3.65 3.63 3.62 3.61 3.60 3.46 3.43 3.33 3.30 3.18 3.04
7.71 7.70 7.60 7.59 7.57 7.54 7.53 7.47 7.42 7.38 7.37 7.28 7.12 7.10 7.09 7.05 7.02 6.80 6.79 6.73
10.87
HO
N
O
HN
0.98
0.94 0.95 0.94 1.90 0.91 0.95 0.97 3.74 1.90 0.95
1.04 1.09 1.00 0.90 2.09
f1 (ppm)

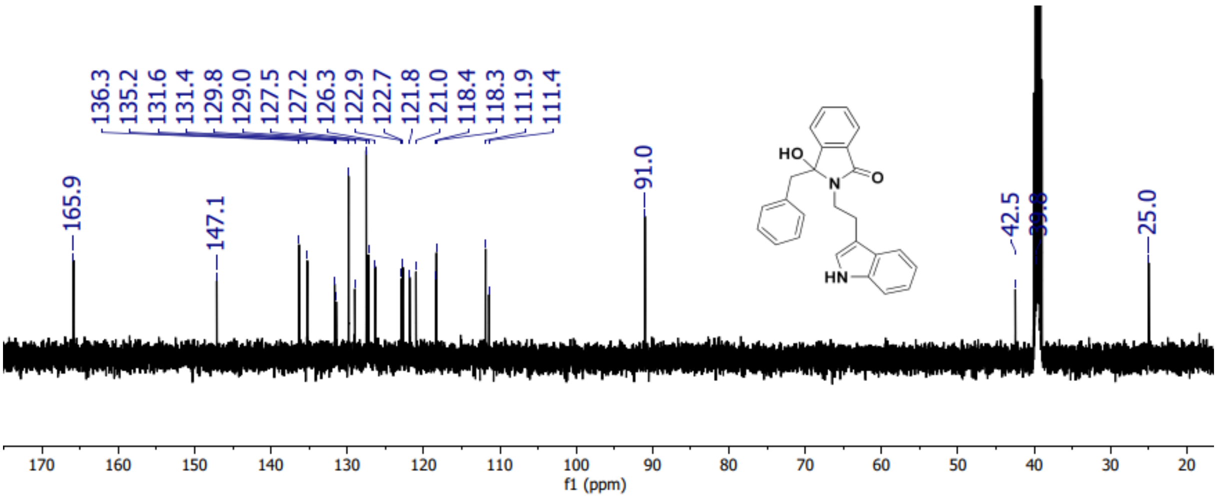
136.3 135.2 131.6 131.4 129.8 129.0 127.5 127.2 126.3 122.9 122.7 121.8 121.0 118.4 118.3 111.9 111.4
165.9
147.1
91.0
42.5
25.0
HO
N
O
HN
f1 (ppm)

3-Benzyl-3-hydroxyisoindolin-1-one (1u)

Following the procedure for the synthesis of 3-hydroxyisoindolin-1-ones **1** using (*Z*)-3-benzylideneisobenzofuran-1(3*H*)-one **2a** (111 mg, 0.5 mmol, 1 equiv.) and ammonium acetate (193 mg, 2.5 mmol, 5 equiv.) with purification using column chromatography (*n*-hexane/ethyl acetate, 50:50), afforded the desired compound **1u.**

White solid; yield: 85 mg (71%); mp: 169-173°C.

IR (KBr): 3410, 3341, 3032, 2924, 1690, 1072, 748 cm^{-1}.

^{1}H-NMR ($CDCl_3$, 500 MHz): δ = 7.45 (t, *J* = 7.5 Hz, 1H, CH_{Ar}), 7.47-7.44 (m, 2H, CH_{Ar}), 7.36 (t, *J* = 7.5 Hz, 1H, CH_{Ar}), 7.25-7.23 (m, 3H, CH_{Ar}), 7.18-7.17 (m, 2H, CH_{Ar}), 6.75 (br s, 1H, NH), 3.89 (br s, 1H, OH), 3.37 (d, *J* = 13.5 Hz, 1H, CH_2), 3.12 (d, *J* = 13.5 Hz, 1H, CH_2).

^{13}C-NMR ($CDCl_3$, 125 MHz): δ = 169.0 (C=O), 148.4 (CH_{Ar}), 134.9 (CH_{Ar}), 132.8 (C_{Ar}), 130.7 ($2CH_{Ar}$), 130.5 (CH_{Ar}), 129.8 (C_{Ar}), 128.4 ($2CH_{Ar}$), 127.3 (C_{Ar}), 123.6 (CH_{Ar}), 122.5 (CH_{Ar}), 87.7 (C), 45.1 (CH_2).

HR-MS (ESI): m/z $[M+H]^+$ cald for $C_{15}H_{14}NO_2^+$: 240.1019, found: 240.1016.

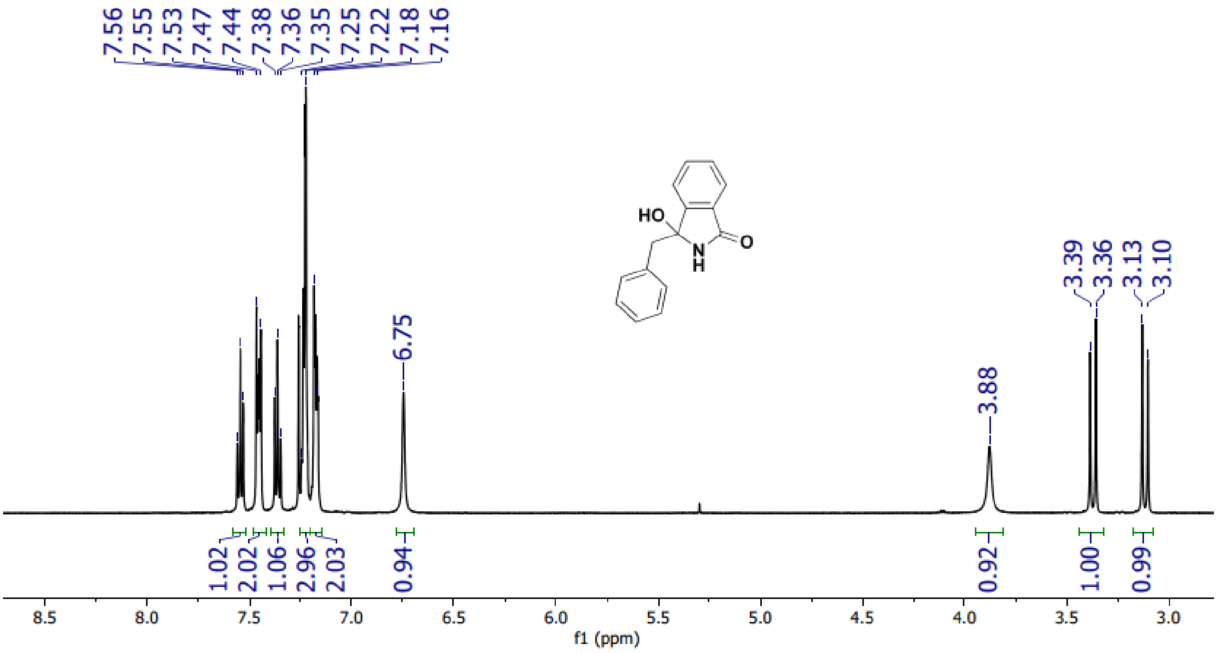
7.56
7.55
7.53
7.47
7.44
7.38
7.36
7.35
7.25
7.22
7.18
7.16
6.75
3.88
3.39
3.36
3.13
3.10
HO
N
H
O
1.02
2.02
1.06
2.96
2.03
0.94
0.92
1.00
0.99
8.5
8.0
7.5
7.0
6.5
6.0
5.5
5.0
4.5
4.0
3.5
3.0
f1 (ppm)

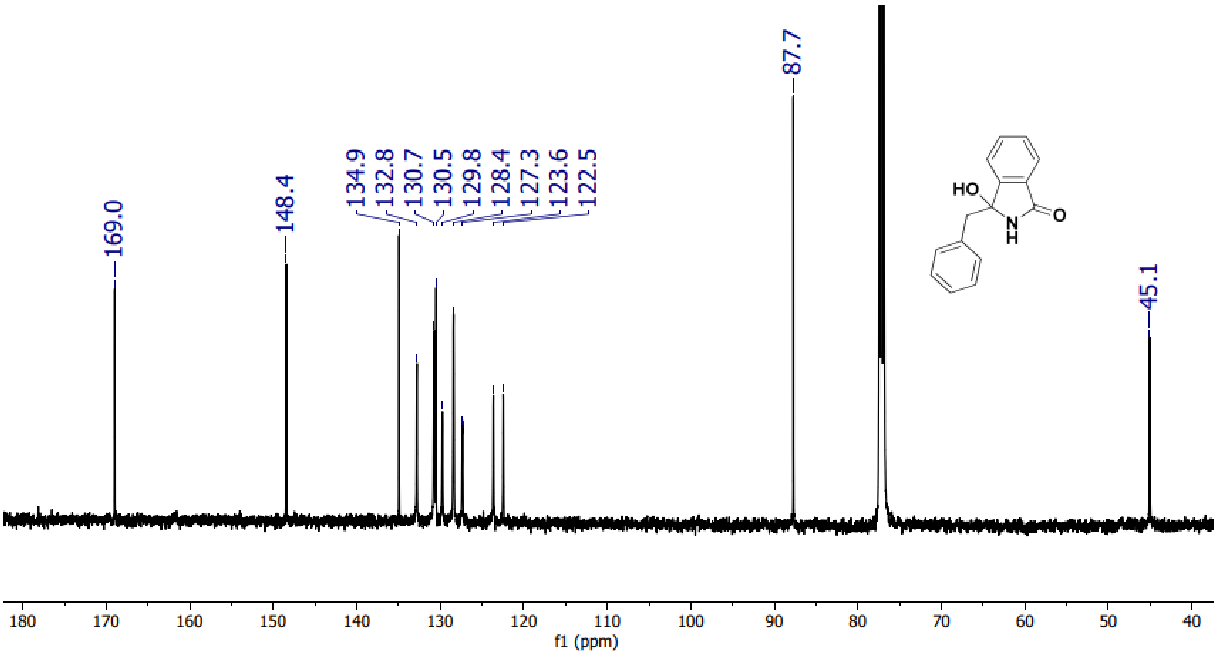
169.0
148.4
134.9
132.8
130.7
130.5
129.8
128.4
127.3
123.6
122.5
87.7
45.1
HO
N
H
O
180
170
160
150
140
130
120
110
100
90
80
70
60
50
40
f1 (ppm)

3-(2-(Benzyloxy)ethyl)-2-butyl-3-hydroxyisoindolin-1-one (1v)

Following the procedure for the synthesis of 3-hydroxyisoindolin-1-ones **1** using (*Z*)-3-(2-(benzyloxy)ethylidene)isobenzofuran-1(3*H*)-one **2b** (133 mg, 0.5 mmol, 1 equiv.) and *n*-butylamine (0.099 mL, 1 mmol, 2 equiv.) with purification using column chromatography (*n*-hexane/ethyl acetate, 70:30), afforded the desired compound **1v**.

Light yellow solid; yield: 141 mg (83%); mp: 90-94°C.

IR (KBr): 3333, 3032, 2963, 2878, 1682, 1412, 1327, 1111, 1026, 748, 702 cm^{-1}.

^{1}H-NMR ($CDCl_3$, 500 MHz): δ = 7.71 (d, *J* = 7.5 Hz, 1H, CH_{Ar}), 7.51 (t, *J* = 7.5 Hz, 1H, CH_{Ar}), 7.44 (t, *J* = 7.5 Hz, 2H, CH_{Ar}), 7.33-7.27 (m, 3H, CH_{Ar}), 7.19 (d, *J* = 7.5 Hz, 2H, CH_{Ar}), 4.37 (d, *J* = 12 Hz, 1H, CH_2), 4.34 (d, *J* = 12 Hz, 1H, CH_2), 3.57-3.51 (m, 1H, CH_2), 3.53 (br s, 1H, OH), 3.38-3.29 (m, 2H, CH_2), 3.21 (ddd, *J* = 14, 10 and 5.5 Hz, 1H, CH_2), 2.61-2.55 (m, 1H, CH_2), 2.28-2.22 (m, 1H, CH_2), 1.76-1.69 (m, 1H, CH_2), 1.66-1.59 (m, 1H, CH_2), 1.40-1.33 (m, 2H, CH_2), 0.93 (t, *J* = 7.5 Hz, 3H, CH_3).

^{13}C-NMR ($CDCl_3$, 125 MHz): δ = 167.3 (C=O), 146.8 (C_{Ar}), 137.5 (C_{Ar}), 132.2 (CH_{Ar}), 131.2 (C_{Ar}), 129.6 (CH_{Ar}), 128.5 ($2CH_{Ar}$), 128.0 (CH_{Ar}), 127.9 ($2CH_{Ar}$), 123.3 (CH_{Ar}), 122.2 (CH_{Ar}), 90.3 (C), 73.5 (CH_2), 65.8 (CH_2), 39.0 (CH_2), 36.5 (CH_2), 31.4 (CH_2), 20.8 (CH_2), 13.9 (CH_3).

HRMS (ESI): m/z $[M+H]^+$ cald for $C_{21}H_{26}NO_3^+$: 340.1907, found: 340.1907.

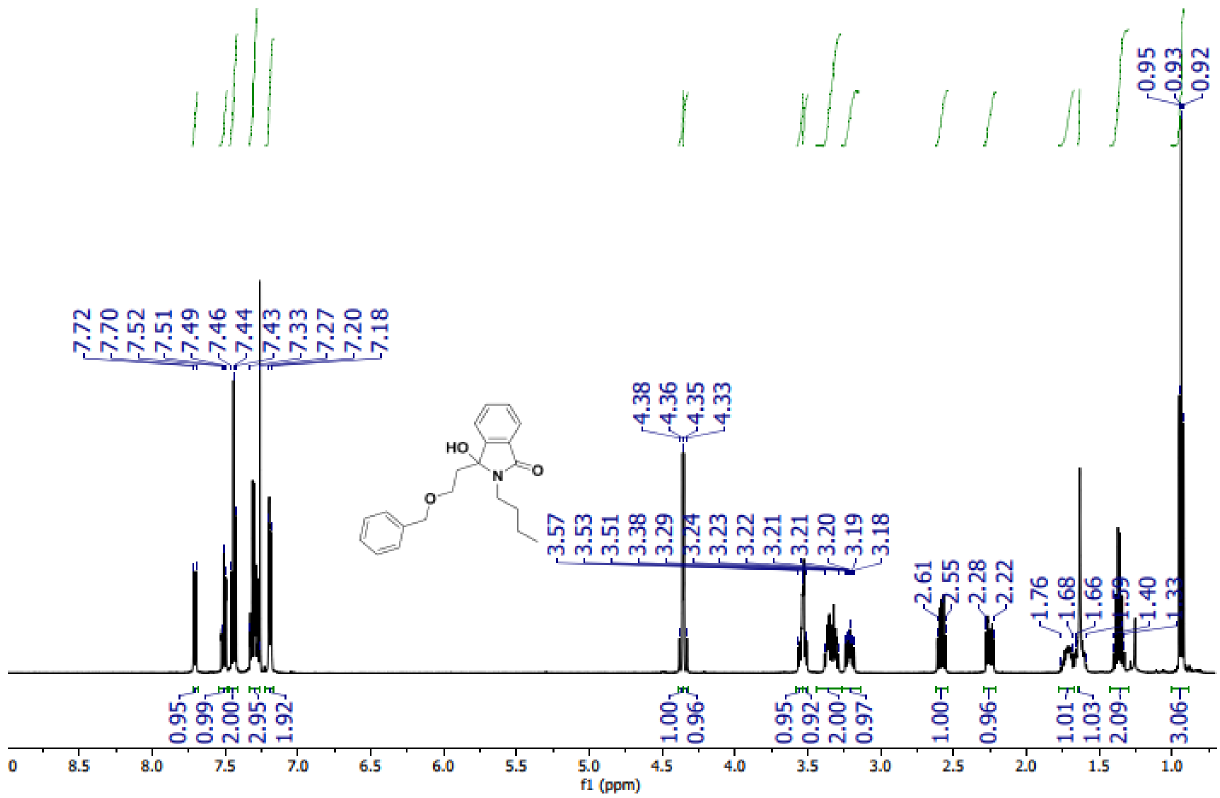

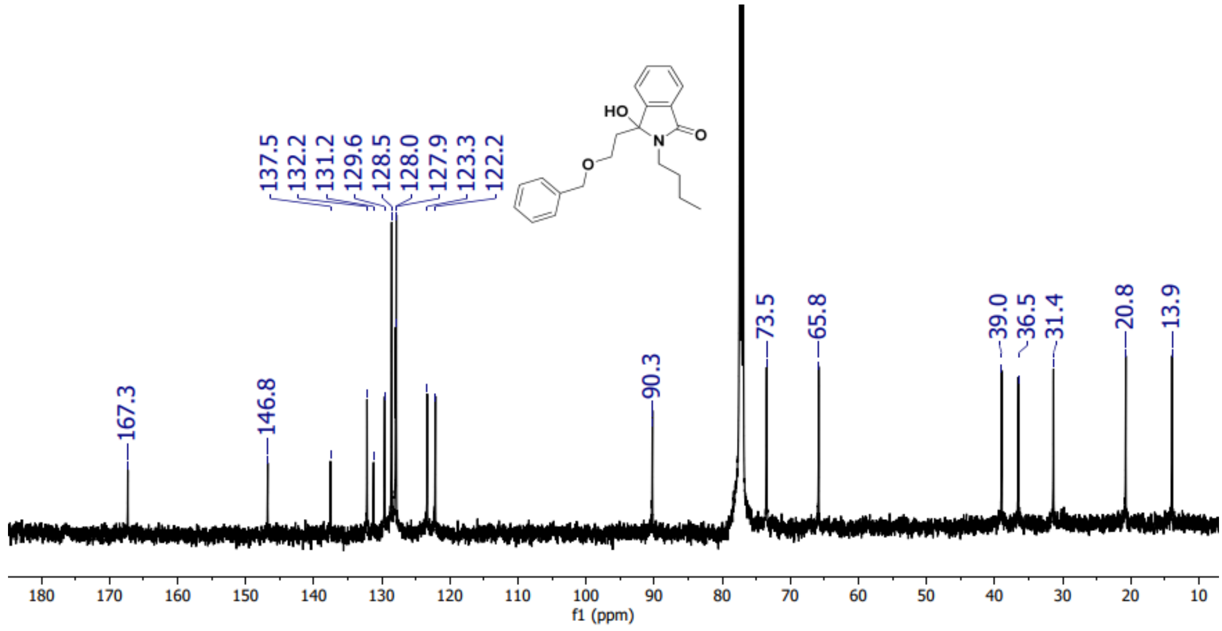

3-(2-(Benzyloxy)ethyl)-3-hydroxy-2-propylisoindolin-1-one (1w)

Following the procedure for the synthesis of 3-hydroxyisoindolin-1-ones using (*Z*)-3-(2-(benzyloxy)ethylidene)isobenzofuran-1(3*H*)-one **2b** (133 mg, 0.5 mmol, 1 equiv.) and propylamine (0.083 mL, 1 mmol, 2 equiv.) with purification using column chromatography (*n*-hexane/ethyl acetate, 70:30), afforded the desired compound **1w.**

Light brown solid; yield: 146 mg (90%); mp: 90-95°C.

IR (KBr): 3364, 3040, 2963, 2878, 1690, 1412, 1080, 1026, 748, 702 cm^{-1}.

^{1}H-NMR ($CDCl_3$, 500 MHz): δ = 7.62 (d, *J* = 7.5 Hz, 1H, CH_{Ar}), 7.50 (td, *J* = 7.5 and 1 Hz, 1H, CH_{Ar}), 7.44-7.39 (m, 2H, CH_{Ar}), 7.32-7.25 (m, 3H, CH_{Ar}), 7.17 (d, *J* = 7.5 Hz, 2H, CH_{Ar}), 4.33 (d, *J* = 11 Hz, 1H, CH_2), 4.31 (d, *J* = 11 Hz, 1H, CH_2), 3.95 (br s, 1H, OH), 3.42 (ddd, *J* = 14, 10.5 and 6 Hz, 1H, CH_2), 3.34-3.29 (m, 1H, CH_2), 3.27-3.22 (m, 1H, CH_2), 3.09 (ddd, *J* = 14, 10.5 and 6 Hz, 1H, CH_2), 2.59-2.53 (m, 1H, CH_2), 2.28-2.23 (m, 1H, CH_2), 1.75-1.67 (m, 1H, CH_2), 1.66-1.58 (m, 1H, CH_2), 0.91 (t, *J* = 7.5 Hz, 3H, CH_3).

^{13}C-NMR ($CDCl_3$, 125 MHz): δ = 167.3 (C=O), 146.6 (C_{Ar}), 137.5 (C_{Ar}), 132.1 (CH_{Ar}), 131.2 (C_{Ar}), 129.5 (CH_{Ar}), 128.5 ($2CH_{Ar}$), 128.0 (CH_{Ar}), 127.9 ($2CH_{Ar}$), 123.3 (CH_{Ar}), 122.1 (CH_{Ar}), 90.2 (C), 73.4 (CH_2), 65.8 (CH_2), 40.8 (CH_2), 36.5 (CH_2), 22.5 (CH_2), 11.9 (CH_3).

HR-MS (ESI): m/z $[M+Na]^+$ cald for $C_{20}H_{23}NO_3Na^+$: 348.1570, found: 348.1570.

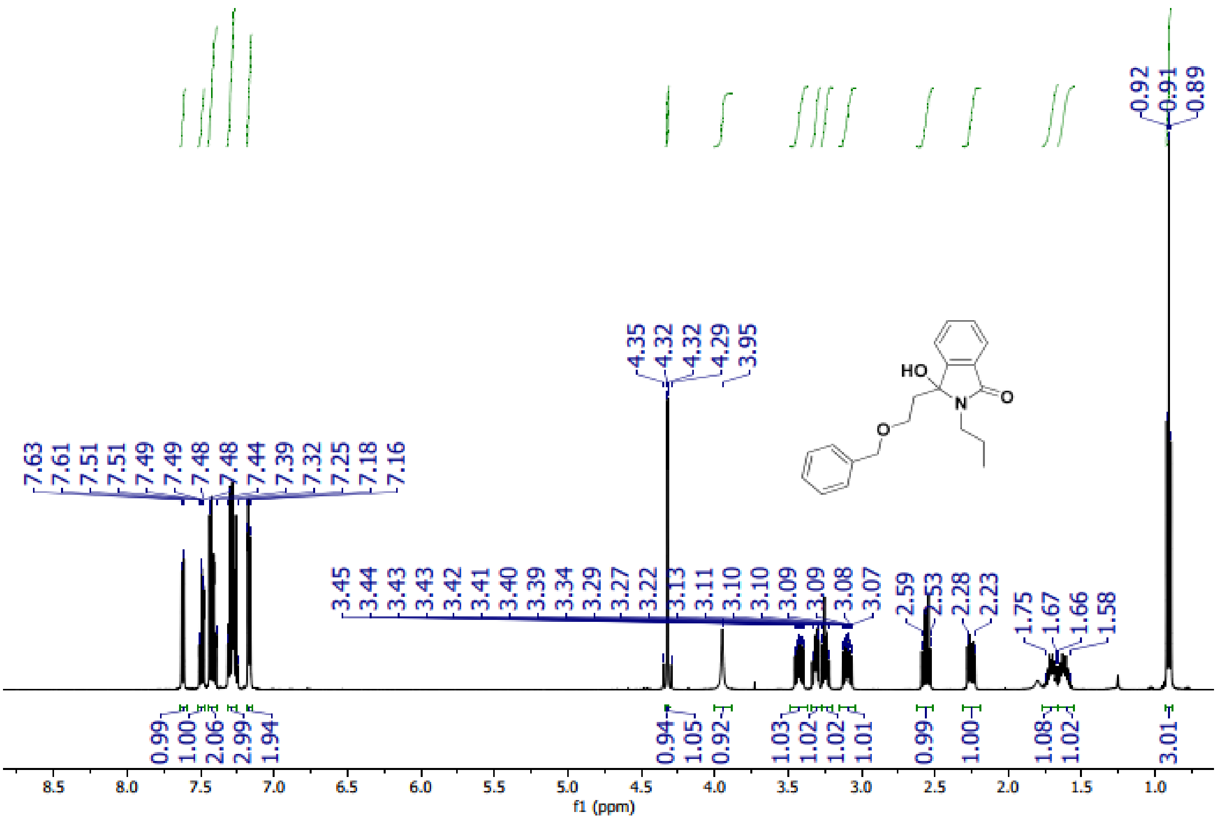

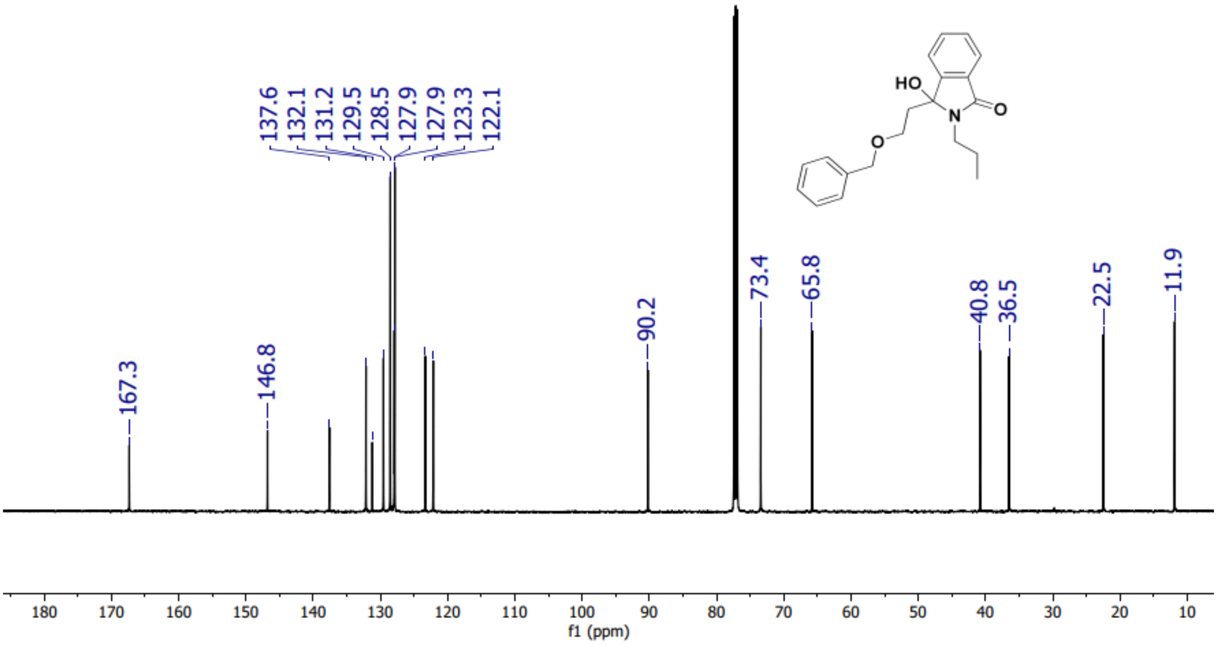

3-(2-(Benzyloxy)ethyl)-3-hydroxy-2-(3-hydroxypropyl)isoindolin-1-one (1x)

Following the procedure for the synthesis of 3-hydroxyisoindolin-1-ones **1** using (*Z*)-3-(2-(benzyloxy)ethylidene)isobenzofuran-1(3*H*)-one **2b** (133 mg, 0.5 mmol, 1 equiv.) and 3-amino-1-propanol (0.077 mL, 1 mmol, 2 equiv.) with purification using column chromatography (*n*-hexane/ethyl acetate, 30:70), afforded the desired compound **1x.**

Light yellow solid; yield: 121 mg (71%); mp: 92-97°C.

IR (KBr): 3372, 3048, 2878, 1682, 1412, 1327, 1219, 1080, 1026, 918, 748, 702 cm^{-1}.

^{1}H-NMR ($CDCl_3$, 500 MHz): δ = 7.61 (d, *J* = 7.5 Hz, 1H, CH_{Ar}), 7.49 (td, *J* = 7.5 and 1 Hz, 1H, CH_{Ar}), 7.42-7.39 (m, 2H, CH_{Ar}), 7.29-7.24 (m, 3H, CH_{Ar}), 7.13 (d, *J* = 7.5 Hz, 2H, CH_{Ar}), 5.05 (br s, 1H, OH), 4.28 (s, 2H, CH_2), 3.90 (br s, 1H, OH), 3.65 (dt, *J* = 14.5 and 6 Hz, 1H, CH_2), 3.44 (br t, *J* = 5 Hz, 2H, CH_2), 3.34 (dt, *J* = 15 and 6 Hz, 1H, CH_2), 3.20 (t, *J* = 6.5 Hz, 2H, CH_2), 2.52 (dt, *J* = 14.5 and 7 Hz, 1H, CH_2), 2.26 (dt, *J* = 14.5 and 6.5 Hz, 1H, CH_2), 1.77-1.73 (m, 2H, CH_2).

^{13}C-NMR ($CDCl_3$, 125 MHz): δ = 168.6 (C=O), 147.1 (C_{Ar}), 137.6 (C_{Ar}), 132.5 (CH_{Ar}), 130.6 (C_{Ar}), 129.5 (CH_{Ar}), 128.5 (2CH_{Ar}), 127.9 (CH_{Ar}), 127.8 (2CH_{Ar}), 123.2 (CH_{Ar}), 122.2 (CH_{Ar}), 90.5 (C), 73.3 (CH_2), 65.5 (CH_2), 59.4 (CH_2), 36.4 (CH_2), 35.3 (CH_2), 31.5 (CH_2).

HR-MS (ESI): m/z $[M+H]^+$ cald for $C_{20}H_{24}NO_4^+$: 342.1700, found: 342.1699.

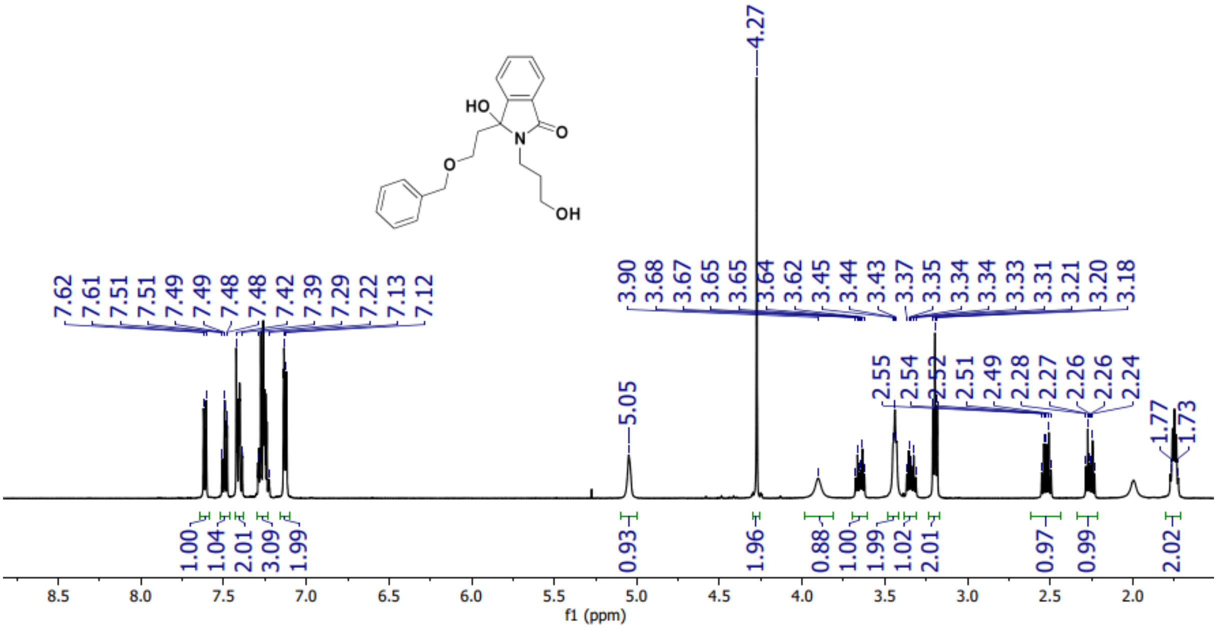
HO
N
O
O
OH
7.62
7.61
7.51
7.51
7.49
7.49
7.48
7.48
7.42
7.39
7.29
7.22
7.13
7.12
5.05
4.27
3.90
3.68
3.67
3.65
3.65
3.64
3.62
3.45
3.44
3.43
3.37
3.35
3.34
3.34
3.33
3.31
3.21
3.20
3.18
2.55
2.54
2.52
2.51
2.49
2.28
2.27
2.26
2.26
2.24
1.77
1.73
1.00
1.04
2.01
3.09
1.99
0.93
1.96
0.88
1.00
1.99
1.02
2.01
0.97
0.99
2.02
f1 (ppm)

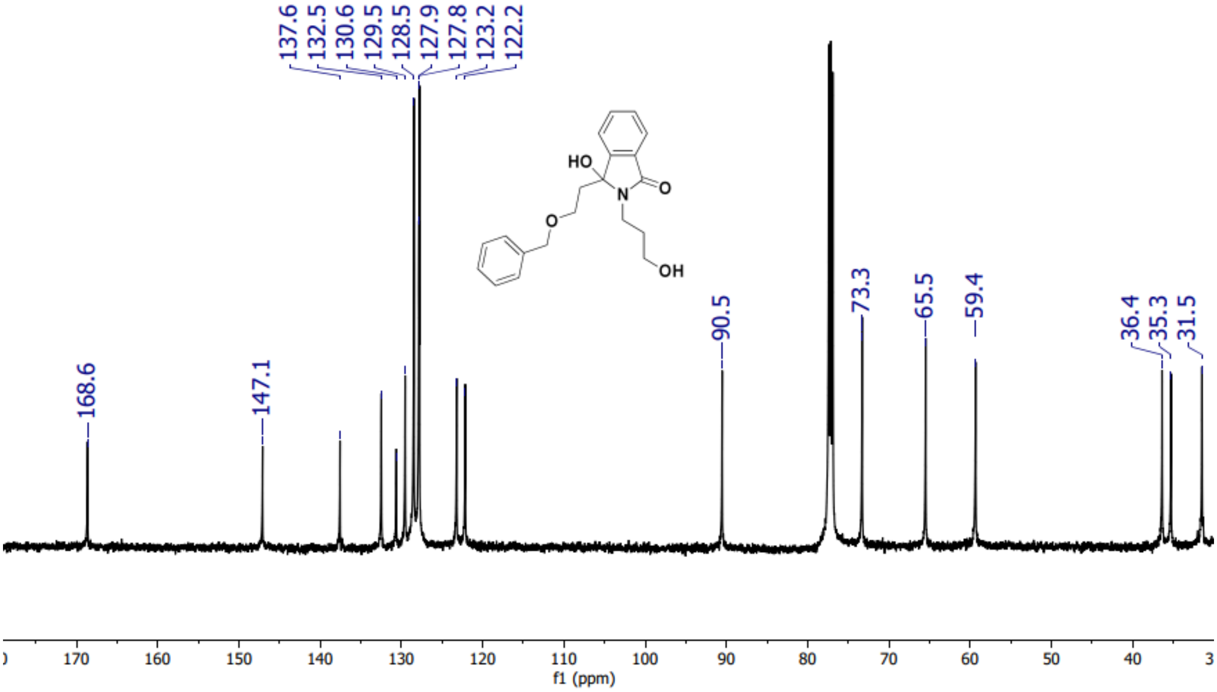
137.6
132.5
130.6
129.5
128.5
127.9
127.8
123.2
122.2
HO
N
O
O
OH
168.6
147.1
90.5
73.3
65.5
59.4
36.4
35.3
31.5
f1 (ppm)

3-(2-(Benzyloxy)ethyl)-3-hydroxy-2-(2-hydroxyethyl)isoindolin-1-one (**1y**)

Following the procedure for the synthesis of 3-hydroxyisoindolin-1-ones **1** using (*Z*)-3-(2-(benzyloxy)ethylidene)isobenzofuran-1(3*H*)-one **2b** (133 mg, 0.5 mmol, 1 equiv.) and ethanolamine (0.060 mL, 1 mmol, 2 equiv.) with purification using column chromatography (*n*-hexane/ethyl acetate, 30:70), afforded the desired compound **1y.**

White solid; yield: 117 mg (72%); mp: 87-90°C.

IR (KBr): 3472, 3287, 3040, 2886, 1690, 1404, 1057, 748, 702 cm^{-1}.

^{1}H-NMR ($CDCl_3$, 500 MHz): δ = 7.67 (d, *J* = 7.5 Hz, 1H, CH_{Ar}), 7.51 (td, *J* = 7.5 and 1 Hz, 1H, CH_{Ar}), 7.44-7.41 (m, 2H, CH_{Ar}), 7.30-7.25 (m, 3H, CH_{Ar}), 7.13 (d, *J* = 7.5 Hz, 2H, CH_{Ar}), 5.05 (br s, 1H, OH), 4.28 (s, 2H, CH_2), 3.94 (br s, 1H, OH), 3.88-3.84 (m, 1H, CH_2), 3.74-3.73 (m, 2H, CH_2), 3.36-3.27 (m, 2H, CH_2), 3.21-3.16 (m, 1H, CH_2), 2.50 (dt, *J* = 14.5 and 7 Hz, 1H, CH_2), 2.32 (dt, *J* = 14.5 and 6 Hz, 1H, CH_2).

^{13}C-NMR ($CDCl_3$, 125 MHz): δ = 168.4 (C=O), 146.8 (C_{Ar}), 137.5 (C_{Ar}), 132.5 (CH_{Ar}), 130.7 (C_{Ar}), 129.6 (CH_{Ar}), 128.5 ($2CH_{Ar}$), 127.9 (CH_{Ar}), 127.8 ($2CH_{Ar}$), 123.3 (CH_{Ar}), 122.1 (CH_{Ar}), 89.8 (C), 73.4 (CH_2), 65.6 (CH_2), 61.4 (CH_2), 42.1 (CH_2), 36.2 (CH_2).

HR-MS (ESI): m/z $[M+H]^+$ cald for $C_{19}H_{22}NO_4^+$: 328.1543, found: 328.1540.

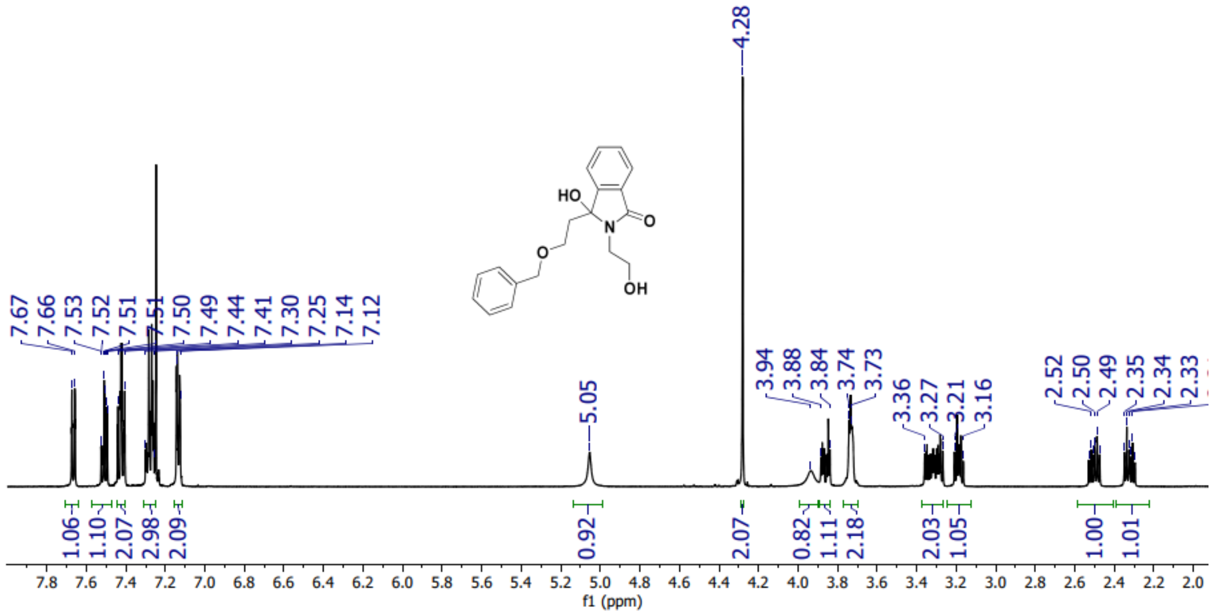

7.67
7.66
7.53
7.52
7.51
7.51
7.50
7.49
7.44
7.41
7.30
7.25
7.14
7.12
5.05
4.28
3.94
3.88
3.84
3.74
3.73
3.36
3.27
3.21
3.16
2.52
2.50
2.49
2.35
2.34
2.33
HO
O
N
O
OH
1.06
1.10
2.07
2.98
2.09
0.92
2.07
0.82
1.11
2.18
2.03
1.05
1.00
1.01
f1 (ppm)

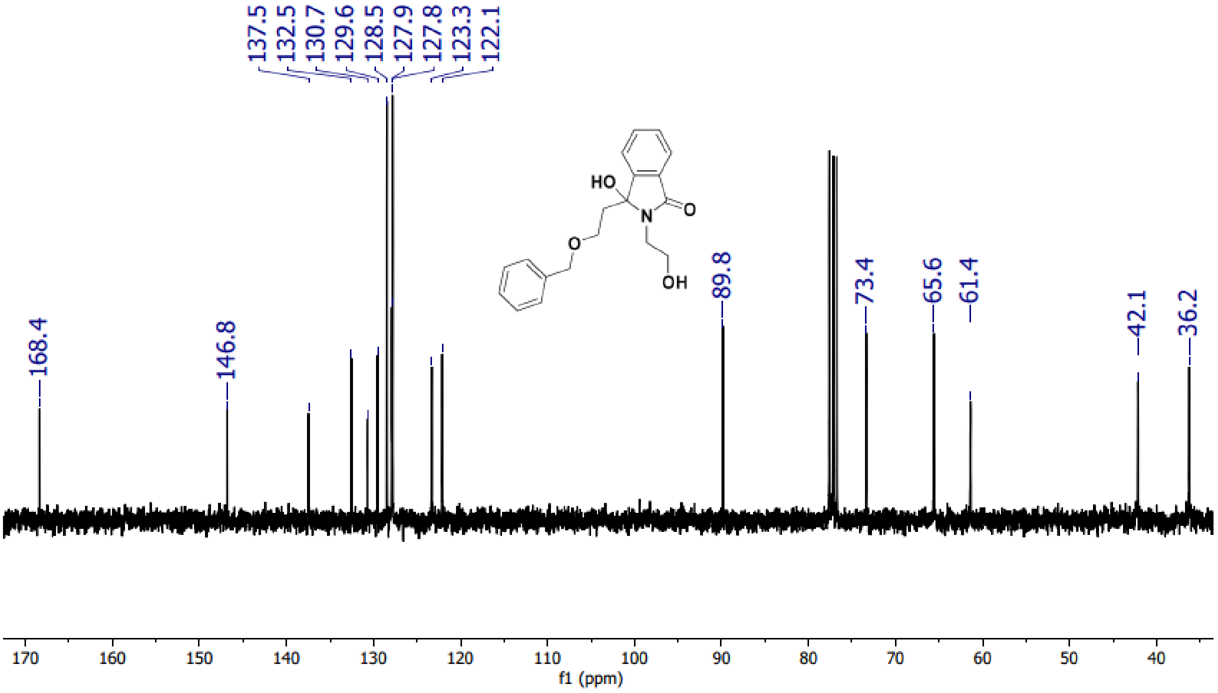

137.5
132.5
130.7
129.6
128.5
127.9
127.8
123.3
122.1
168.4
146.8
89.8
73.4
65.6
61.4
42.1
36.2
HO
O
N
O
OH
f1 (ppm)

2-Benzyl-3-(2-(benzyloxy)ethyl)-3-hydroxyisoindolin-1-one (1z)

Following the procedure for the synthesis of 3-hydroxyisoindolin-1-ones **1** using (*Z*)-3-(2-(benzyloxy)ethylidene)isobenzofuran-1(3*H*)-one **2b** (133 mg, 0.5 mmol, 1 equiv.) and benzylamine (0.11 mL, 1 mmol, 2 equiv.) with purification using column chromatography (*n*-hexane/ethyl acetate, 50:50), afforded the desired compound **1z.**

Yellowish brown solid; yield: 141 mg (76%); mp: 118-122°C.

IR (KBr): 3287, 3032, 2924, 1674, 1420, 1227, 1072, 972, 702 cm^{-1}.

^{1}H-NMR ($CDCl_3$, 500 MHz): δ = 7.72 (d, J = 8 Hz, 1H, CH_{Ar}), 7.51 (td, J = 8 and 1 Hz, 1H, CH_{Ar}), 7.45-7.42 (m, 2H, CH_{Ar}), 7.39 (d, J = 7 Hz, 2H, CH_{Ar}), 7.30-7.24 (m, 5H, CH_{Ar}), 7.22-7.19 (m, 1H, CH_{Ar}), 7.10-7.09 (d, J = 8 Hz, 2H, CH_{Ar}), 4.63 (d, J = 15.5 Hz, 1H, CH_2), 4.52 (d, J = 15.5 Hz, 1H, CH_2), 4.17 (d, J = 11.5 Hz, 1H, CH_2), 4.11 (d, J = 11.5 Hz, 1H, CH_2), 3.89 (br s, 1H, OH), 3.13-3.09 (m, 1H, CH_2), 3.03-2.99 (m, 1H, CH_2), 2.45-2.40 (m, 1H, CH_2), 2.18 (dt, J = 14.5 and 6 Hz, 1H, CH_2).

^{13}C-NMR ($CDCl_3$, 125 MHz): δ = 167.8 (C=O), 147.0 (C_{Ar}), 138.5 (C_{Ar}), 137.6 (C_{Ar}), 132.5 (CH_{Ar}), 130.7 (C_{Ar}), 129.6 (CH_{Ar}), 128.6 ($2CH_{Ar}$), 128.5 ($2CH_{Ar}$), 128.4 ($2CH_{Ar}$), 128.3 (CH_{Ar}), 127.8 ($2CH_{Ar}$), 127.4 (CH_{Ar}), 123.5 (CH_{Ar}), 122.4 (CH_{Ar}), 90.5 (C), 73.2 (CH_2), 65.7 (CH_2), 42.2 (CH_2), 36.8 (CH_2).

HR-MS (ESI): m/z $[M+H]^+$ cald for $C_{24}H_{24}NO_3^+$: 374.1751, found: 374.1748.

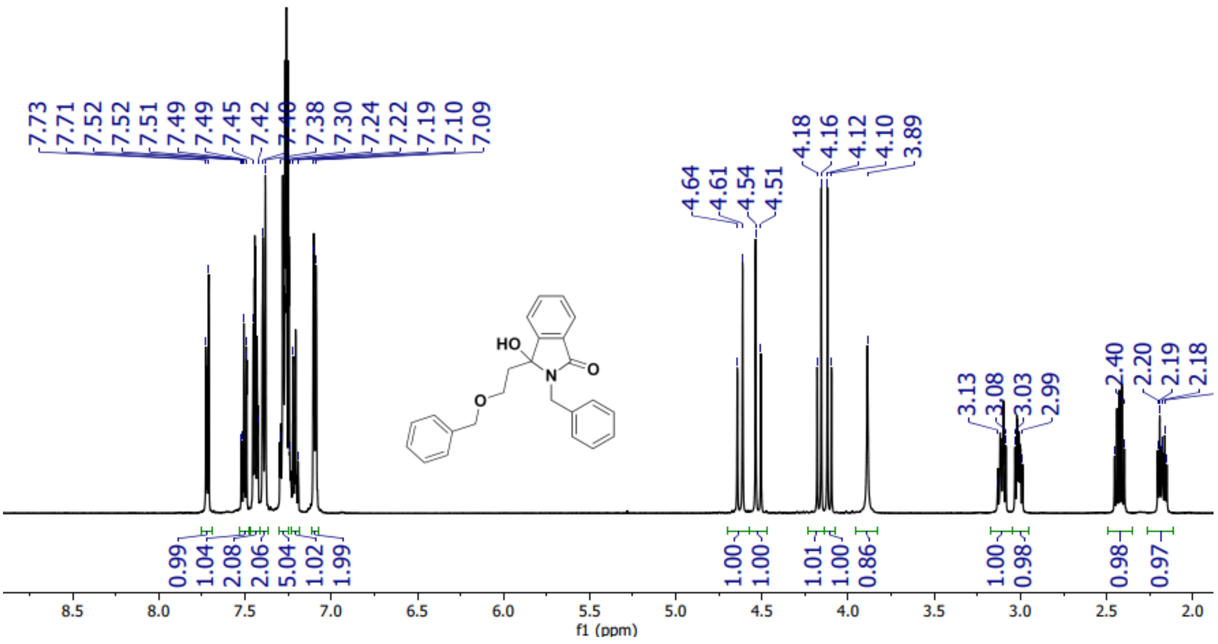
7.73 7.71 7.52 7.52 7.51 7.49 7.49 7.45 7.42 7.40 7.38 7.30 7.24 7.22 7.19 7.10 7.09
4.64 4.61 4.54 4.51
4.18 4.16 4.12 4.10 3.89
3.13 3.08 3.03 2.99
2.40 2.20 2.19 2.18
HO
N
O
O
0.99 1.04 2.08 2.06 5.04 1.02 1.99
1.00 1.00
1.01 1.00 0.86
1.00 0.98
0.98 0.97
8.5 8.0 7.5 7.0 6.5 6.0 5.5 5.0 4.5 4.0 3.5 3.0 2.5 2.0
f1 (ppm)

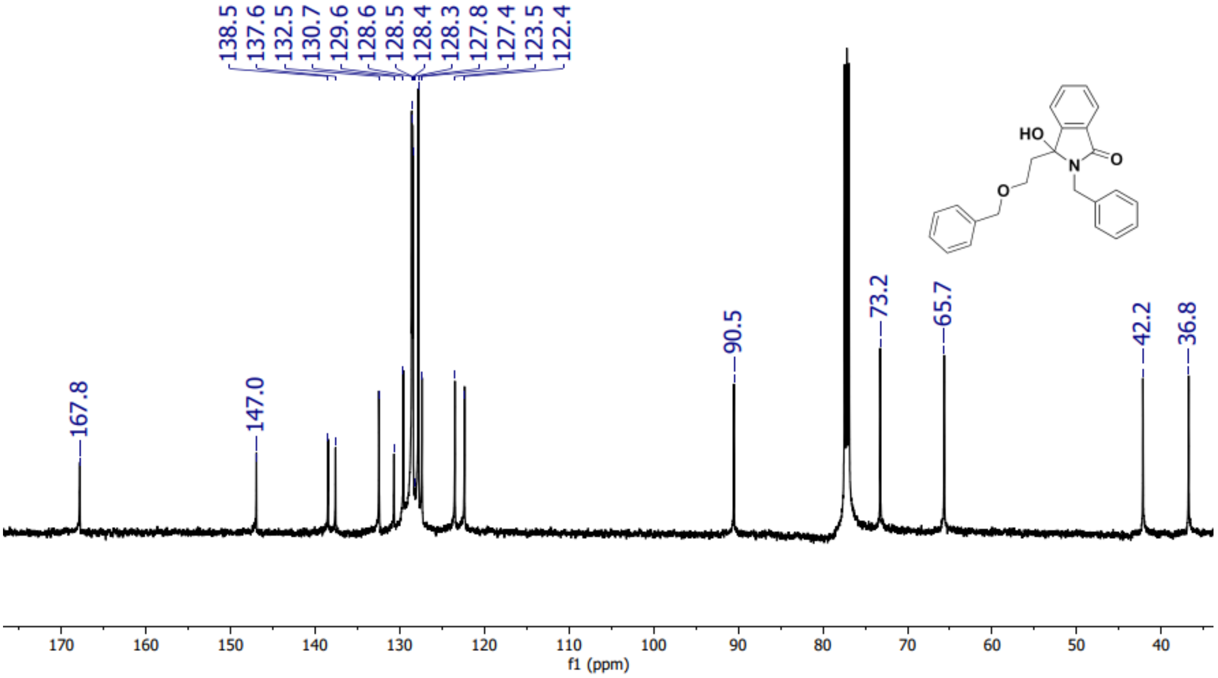
138.5 137.6 132.5 130.7 129.6 128.6 128.5 128.4 128.3 127.8 127.4 123.5 122.4
167.8
147.0
90.5
73.2
65.7
42.2
36.8
HO
N
O
O
170 160 150 140 130 120 110 100 90 80 70 60 50 40
f1 (ppm)

3-(2-(Benzyloxy)ethyl)-3-hydroxy-2-(2-methoxybenzyl)isoindolin-1-one (1aa)

Following the procedure for the synthesis of 3-hydroxyisoindolin-1-ones **1** using (*Z*)-3-(2-(benzyloxy)ethylidene)isobenzofuran-1(3*H*)-one **2b** (133 mg, 0.5 mmol, 1 equiv.) and 2-methoxybenzylamine (0.130 mL, 1 mmol, 2 equiv.) with purification using column chromatography (*n*-hexane/ethyl acetate, 50:50), afforded the desired compound **1aa.**

Light yellow solid; yield: 169 mg (84%); mp: 118-123°C.

IR (KBr): 3356, 3063, 2893, 1682, 1412, 1242, 1111, 1026, 702 cm^{-1}.

^{1}H-NMR ($CDCl_3$, 500 MHz): δ = 7.79 (d, *J* = 7.5 Hz, 1H, CH_{Ar}), 7.53 (td, *J* = 7.5 and 1 Hz, 1H, CH_{Ar}), 7.49-7.45 (m, 2H, CH_{Ar}), 7.43 (dd, *J* = 7.5 and 1.5 Hz, 1H, CH_{Ar}), 7.32-7.25 (m, 3H, CH_{Ar}), 7.21 (td, *J* = 8.5 and 1.5 Hz, 1H, CH_{Ar}), 7.16-7.15 (m, 2H, CH_{Ar}), 6.90 (td, *J* = 8.5 and 1 Hz, 1H, CH_{Ar}), 6.87 (d, *J* = 8.5 Hz, 1 H, CH_{Ar}), 4.85 (d, *J* = 15.5 Hz, 1H, CH_2), 4.60 (d, *J* = 15.5 Hz, 1H, CH_2), 4.27 (d, *J* = 11.5 Hz, 1H, CH_2), 4.23 (d, *J* = 11.5 Hz, 1H, CH_2), 4.02 (br s, 1H, OH), 3.89 (s, 3H, CH_3), 3.25-3.20 (m, 1H, CH_2), 3.19-3.15 (m, 1H, CH_2), 2.53-2.47 (m, 1H, CH_2), 2.37 (dt, *J* = 14.5 and 6 Hz, 1H, CH_2).

^{13}C-NMR ($CDCl_3$, 125 MHz): δ = 167.8 (C=O), 156.6 (C_{Ar}), 146.9 (C_{Ar}), 137.7 (C_{Ar}), 132.3 (CH_{Ar}), 131.0 (C_{Ar}), 130.3 (CH_{Ar}), 129.5 (CH_{Ar}), 128.6 (CH_{Ar}), 128.4 (2CH_{Ar}), 127.8 (3CH_{Ar}), 126.3 (C_{Ar}), 123.5 (CH_{Ar}), 122.2 (CH_{Ar}), 121.1 (CH_{Ar}), 110.6 (CH_{Ar}), 90.3 (C), 73.3 (CH_2), 66.0 (CH_2), 55.6 (CH_3), 36.8 (CH_2), 36.4 (CH_2).

HR-MS (ESI): m/z $[M+H]^+$ cald for $C_{25}H_{26}NO_4^+$: 404.1856, found: 404.1850.

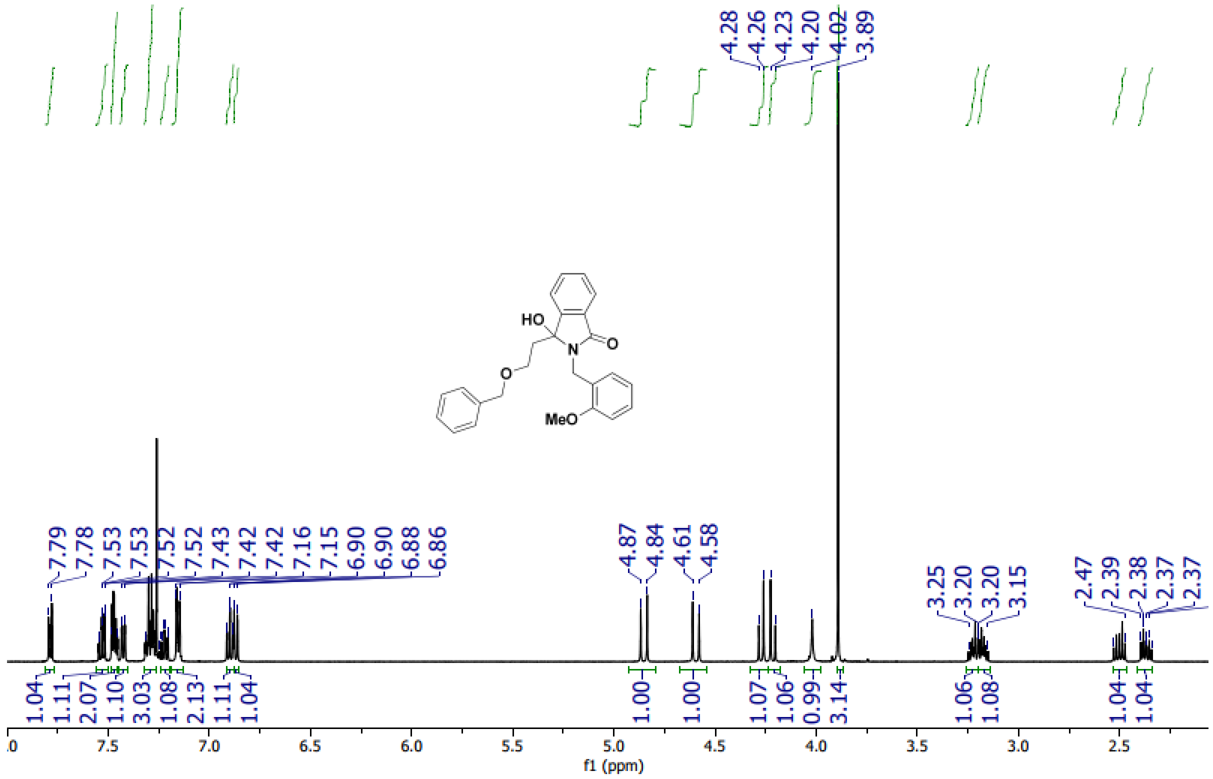
7.79 7.78 7.53 7.53 7.52 7.52 7.43 7.42 7.42 7.16 7.15 6.90 6.90 6.88 6.86
4.87 4.84 4.61 4.58
4.28 4.26 4.23 4.20 4.02 3.89
3.25 3.20 3.20 3.15
2.47 2.39 2.38 2.37 2.37
1.04 1.11 2.07 1.10 3.03 1.08 2.13 1.11 1.04 1.00 1.00 1.07 1.06 0.99 3.14 1.06 1.08 1.04 1.04
HO
N
O
MeO
f1 (ppm)

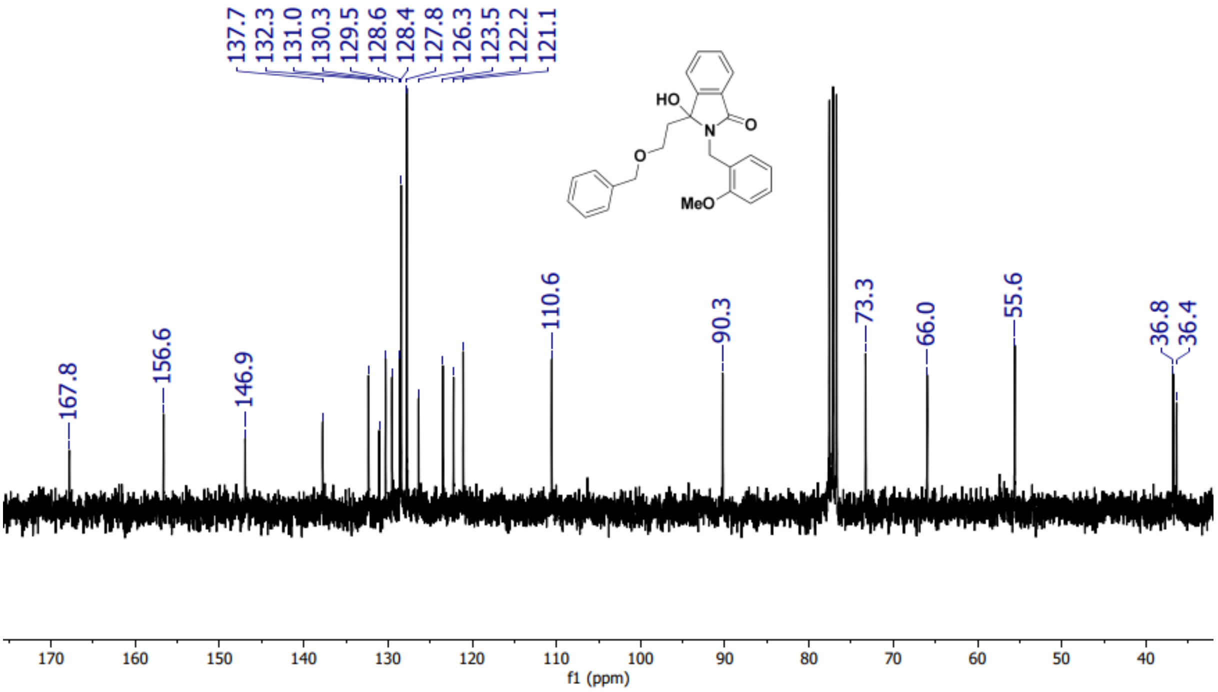
137.7 132.3 131.0 130.3 129.5 128.6 128.4 127.8 126.3 123.5 122.2 121.1
167.8 156.6 146.9 110.6 90.3 73.3 66.0 55.6 36.8 36.4
HO
N
O
MeO
f1 (ppm)

3-(2-(Benzyloxy)ethyl)-3-hydroxy-2-(4-methoxybenzyl)isoindolin-1-one (1ab)

Following the procedure for the synthesis of 3-hydroxyisoindolin-1-ones **1** using (*Z*)-3-(2-(benzyloxy)ethylidene)isobenzofuran-1(3*H*)-one **2b** (133 mg, 0.5 mmol, 1 equiv.) and 4-methoxybenzylamine (0.130 mL, 1 mmol, 2 equiv.) with purification using column chromatography (*n*-hexane/ethyl acetate, 50:50), afforded the desired compound **1ab.**

Light yellow solid; yield: 179 mg (89%); mp: 117-121 °C.

IR (KBr): 3318, 2931, 1674, 1412, 1242, 1180, 1103, 817, 756, 702 cm^{-1}.

^{1}H-NMR ($CDCl_3$, 500 MHz): δ = 7.73 (d, *J* = 7.5 Hz, 1H, CH_{Ar}), 7.51 (td, *J* = 7.5 and 1 Hz, 1H, CH_{Ar}), 7.46-7.42 (m, 2H, CH_{Ar}), 7.34 (d, *J* = 8.5 Hz, 2H, CH_{Ar}), 7.30-7.23 (m, 3H, CH_{Ar}), 7.11-7.10 (m, 2H, CH_{Ar}), 6.78 (d, *J* = 8.5 Hz, 2H, CH_{Ar}), 4.60 (d, *J* = 11 Hz, 1H, CH_2), 4.51 (d, *J* = 11 Hz, 1H, CH_2), 4.18 (d, *J* = 11.5 Hz, 1H, CH_2), 4.14 (d, *J* = 11.5 Hz, 1H, CH_2), 3.73 (s, 3H, CH_3), 3.69 (br s, 1H, OH), 3.14-3.10 (m, 1H, CH_2), 3.06-3.01 (m, 1H, CH_2), 2.48-2.42 (m, 1H, CH_2), 2.19 (dt, *J* = 14.5 and 6 Hz, 1H, CH_2).

^{13}C-NMR ($CDCl_3$, 125 MHz): δ = 167.6 (C=O), 158.9 (C_{Ar}), 147.0 (C_{Ar}), 137.7 (C_{Ar}), 132.4 (CH_{Ar}), 130.9 (C_{Ar}), 130.8 (CH_{Ar}), 130.7 (CH_{Ar}), 130.0 ($2CH_{Ar}$), 129.6 (CH_{Ar}), 128.5 ($2CH_{Ar}$), 127.8 ($2CH_{Ar}$), 123.5 (C_{Ar}), 122.3 (CH_{Ar}), 113.9 ($2CH_{Ar}$), 90.5 (C), 73.3 (CH_2), 65.8 (CH_2), 55.3 (CH_3), 41.6 (CH_2), 36.8 (CH_2).

HR-MS (ESI): m/z $[M+H]^+$ cald for $C_{25}H_{26}NO_4^+$: 404.1856, found: 404.1851.

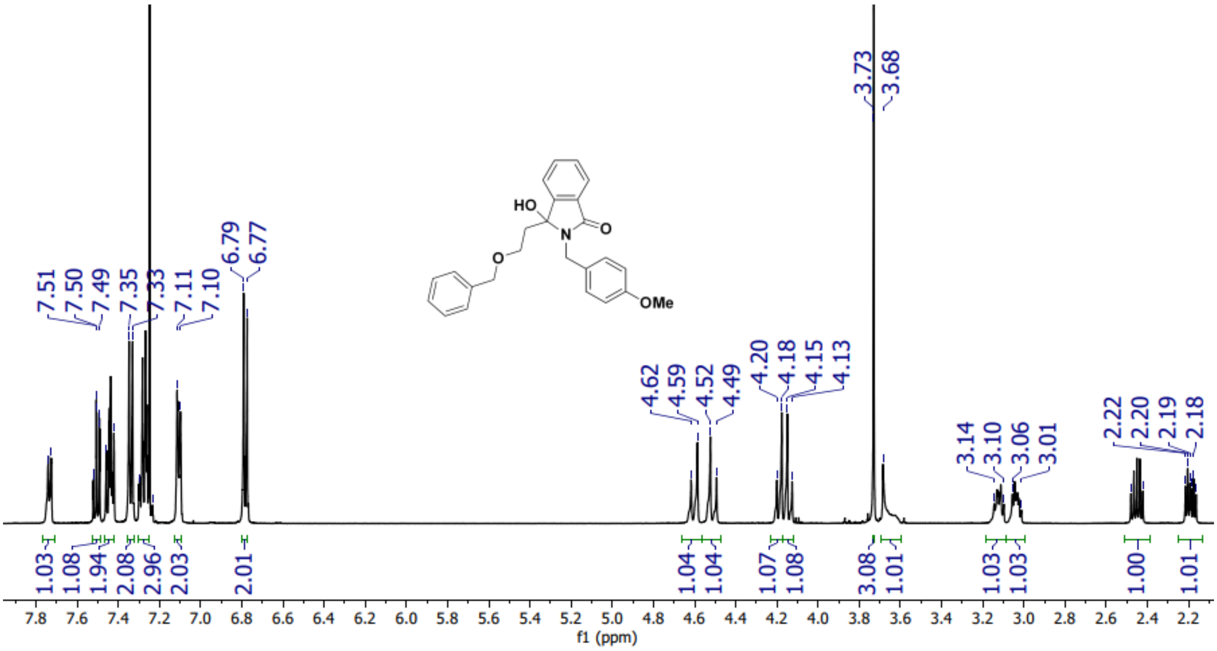
HO
N
O
O
OMe
7.51
7.50
7.49
7.35
7.33
7.11
7.10
6.79
6.77
4.62
4.59
4.52
4.49
4.20
4.18
4.15
4.13
3.73
3.68
3.14
3.10
3.06
3.01
2.22
2.20
2.19
2.18
1.03
1.08
1.94
2.08
2.96
2.03
2.01
1.04
1.04
1.07
1.08
3.08
1.01
1.03
1.03
1.00
1.01
7.8 7.6 7.4 7.2 7.0 6.8 6.6 6.4 6.2 6.0 5.8 5.6 5.4 5.2 5.0 4.8 4.6 4.4 4.2 4.0 3.8 3.6 3.4 3.2 3.0 2.8 2.6 2.4 2.2
f1 (ppm)

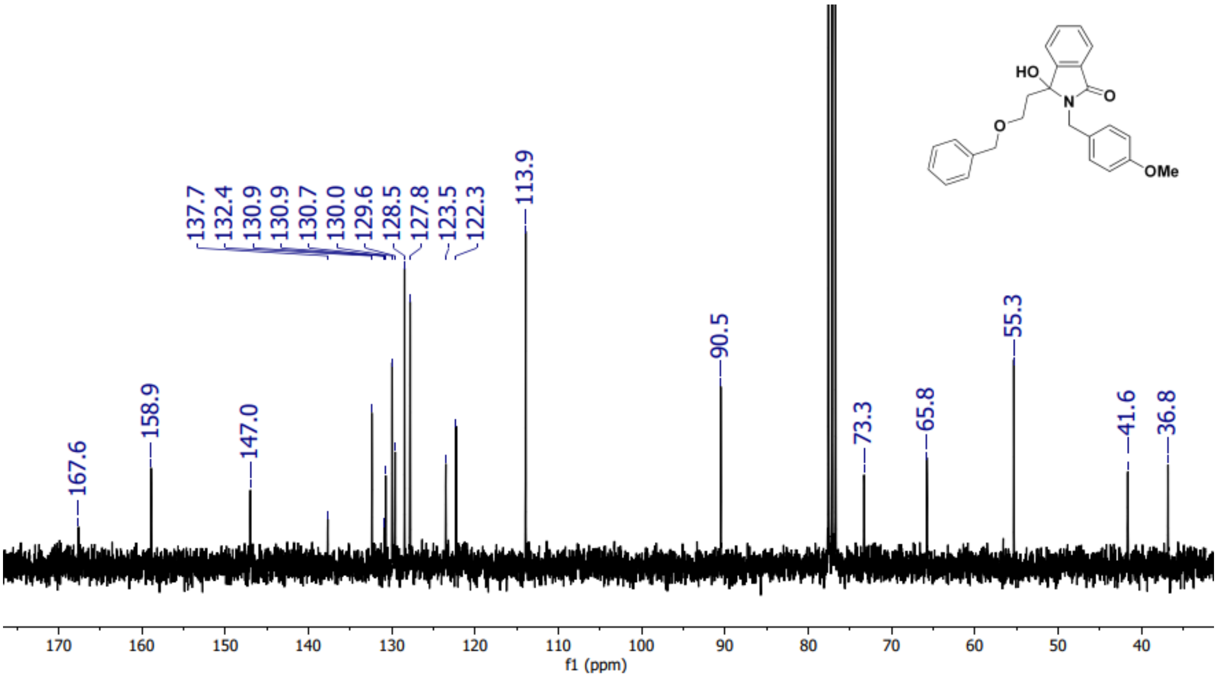
HO
N
O
O
OMe
167.6
158.9
147.0
137.7
132.4
130.9
130.9
130.7
130.0
129.6
128.5
127.8
123.5
122.3
113.9
90.5
73.3
65.8
55.3
41.6
36.8
170 160 150 140 130 120 110 100 90 80 70 60 50 40
f1 (ppm)

3-(2-(Benzyloxy)ethyl)-3-hydroxy-2-phenethylisoindolin-1-one (1ac)

Following the procedure for the synthesis of 3-hydroxyisoindolin-1-ones **1** using (*Z*)-3-(2-(benzyloxy)ethylidene)isobenzofuran-1(3*H*)-one **2b** (133 mg, 0.5 mmol, 1 equiv.) and 2-phenethylamine (0.126 mL, 1 mmol, 2 equiv.) with purification using column chromatography (*n*-hexane/ethyl acetate, 50:50), afforded the desired compound **1ac.**

White solid; yield: 168 mg (87%); mp: 157-160 °C.

IR (KBr): 3364, 3032, 2878, 1682, 1420, 1080, 1026, 756, 702 cm^{-1}.

^{1}H-NMR ($CDCl_3$, 500 MHz): δ = 7.75 (d, *J* = 7.5 Hz, 1H, CH_{Ar}), 7.50 (td, *J* = 7.5 and 1 Hz, 1H, CH_{Ar}), 7.45 (td, *J* = 7.5 and 1 Hz, 1H, CH_{Ar}), 7.41 (d, *J* = 7.5 Hz, 1H, CH_{Ar}), 7.32-7.25 (m, 7H, CH_{Ar}), 7.22-7.20 (m, 1H, CH_{Ar}), 7.19-7.17 (m, 2H, CH_{Ar}), 4.35 (d, *J* = 12 Hz, 1H, CH_2), 4.31 (d, *J* = 12 Hz, 1H, CH_2), 3.84 (ddd, *J* = 14.5, 10 and 5.5 Hz, 1H, CH_2), 3.44 (ddd, *J* = 14.5, 10 and 6 Hz, 1H, CH_2), 3.29 (t, *J* = 6.5 Hz, 2H, CH_2), 3.16-3.10 (m, 1H, CH_2), 3.05 (br s, 1H, OH), 3.04-2.98 (m, 1H, CH_2), 2.50 (dt, *J* = 14.5 and 7 Hz, 1H, CH_2), 2.22 (dt, *J* = 14.5 and 6 Hz, 1H, CH_2).

^{13}C-NMR ($CDCl_3$, 125 MHz): δ = 167.3 (C=O), 146.8 (C_{Ar}), 139.6 (C_{Ar}), 137.6 (C_{Ar}), 132.3 (CH_{Ar}), 131.3 (C_{Ar}), 129.7 (CH_{Ar}), 129.1 (2CH_{Ar}), 128.7 (2CH_{Ar}), 128.6 (2CH_{Ar}), 128.0 (CH_{Ar}), 127.9 (2CH_{Ar}), 126.6 (CH_{Ar}), 123.4 (CH_{Ar}), 122.2 (CH_{Ar}), 90.2 (C), 73.5 (CH_2), 65.8 (CH_2), 41.2 (CH_2), 36.6 (CH_2), 35.5 (CH_2).

HR-MS (ESI): m/z $[M+Na]^+$ cald for $C_{25}H_{25}NO_3Na^+$: 410.1727, found: 410.1726.

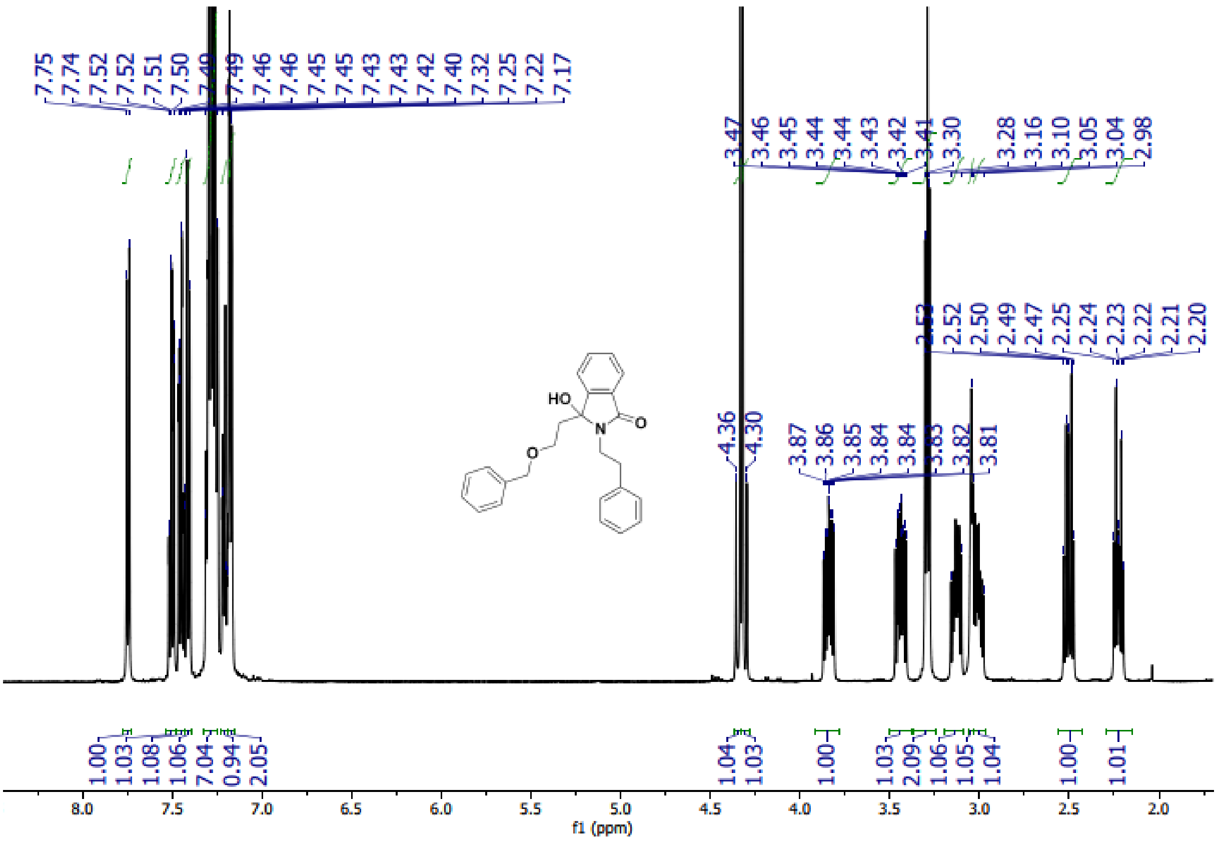
7.75 7.74 7.52 7.52 7.51 7.50 7.49 7.49 7.46 7.46 7.45 7.45 7.43 7.43 7.42 7.40 7.32 7.25 7.22 7.17
3.47 3.46 3.45 3.44 3.44 3.43 3.42 3.41 3.30 3.28 3.16 3.10 3.05 3.04 2.98
2.52 2.50 2.49 2.47 2.25 2.24 2.23 2.22 2.21 2.20
4.36 4.30
3.87 3.86 3.85 3.84 3.84 3.83 3.82 3.81
HO
O
N
1.00 1.03 1.08 1.06 7.04 0.94 2.05 1.04 1.03 1.00 1.03 2.09 1.06 1.05 1.04 1.00 1.01
8.0 7.5 7.0 6.5 6.0 5.5 5.0 4.5 4.0 3.5 3.0 2.5 2.0
f1 (ppm)

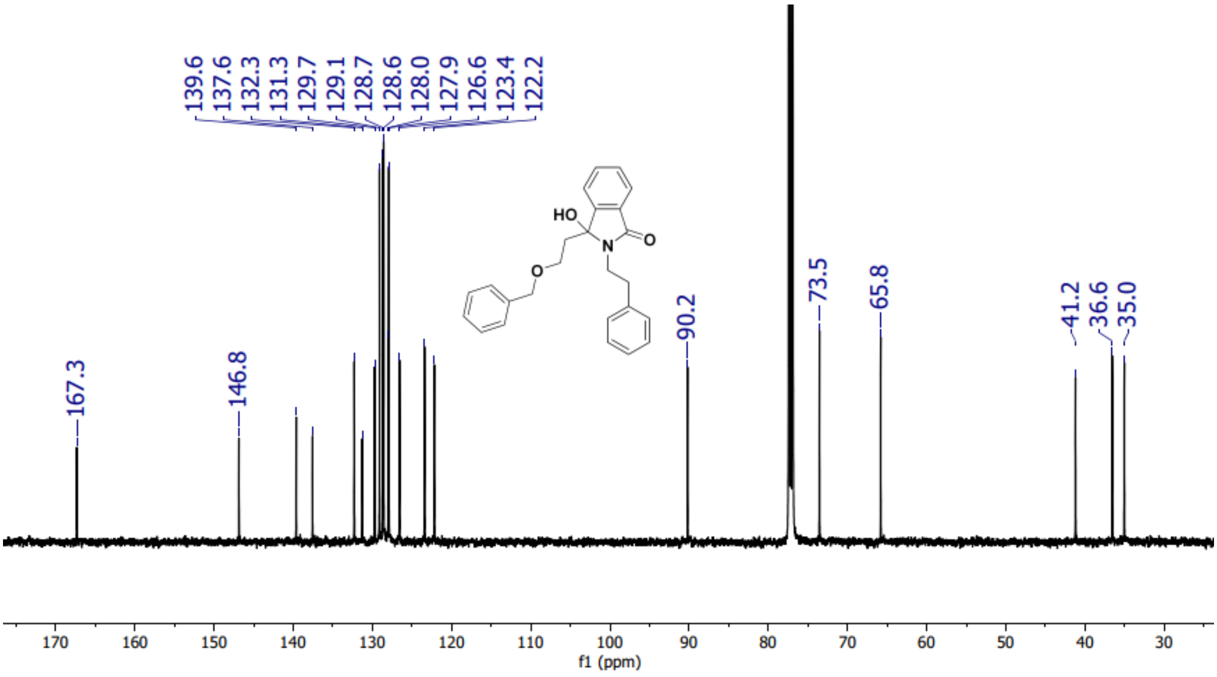
139.6 137.6 132.3 131.3 129.7 129.1 128.7 128.6 128.0 127.9 126.6 123.4 122.2
HO
O
N
167.3
146.8
90.2
73.5
65.8
41.2 36.6 35.0
170 160 150 140 130 120 110 100 90 80 70 60 50 40 30
f1 (ppm)

2-(2-(1H-indol-3-yl)ethyl)-3-(2-(benzyloxy)ethyl)-3-hydroxyisoindolin-1-one (1ad)

Following the procedure for the synthesis of 3-hydroxyisoindolin-1-ones **1** using (*Z*)-3-(2-(benzyloxy)ethylidene)isobenzofuran-1(3*H*)-one **2b** (133 mg, 0.5 mmol, 1 equiv.) and tryptamine (160 mg, 1 mmol, 2 equiv.) with purification using column chromatography (*n*-hexane/ethyl acetate, 50:50), afforded the desired compound **1ad.**

Light brown viscous oil; yield: 162 mg (76%).

IR (nujol): 3418, 3055, 2932, 1682, 1420, 1227, 1096, 741, 702 cm^{-1}.

^{1}H-NMR ($CDCl_3$, 500 MHz): δ = 8.05 (br s, 1H, NH), 7.43 (t, *J* = 7.5 Hz, 2H, CH_{Ar}), 7.50 (td, *J* = 7.5 and 1.5 Hz, 1H, CH_{Ar}), 7.46-7.41 (m, 2H, CH_{Ar}), 7.34 (d, *J* = 7.5 Hz, 1H, CH_{Ar}), 7.30-7.24 (m, 3H, CH_{Ar}), 7.20-7.15 (m, 3H, CH_{Ar}), 7.11 (t, *J* = 7.5 Hz, 1H, CH_{Ar}), 7.04 (d, *J* = 1.5 Hz, 1H, CH_{Ar}), 4.33 (d, *J* = 11.5 Hz, 1H, CH_2), 4.28 (d, *J* = 11.5 Hz, 1H, CH_2), 3.93 (ddd, *J* = 14, 9.5 and 6 Hz, 1H, CH_2), 3.56 (ddd, *J* = 14, 10 and 6.5 Hz, 1H, CH_2), 3.34 (br s, 1H, OH), 3.31-3.26 (m, 2H, CH_2), 3.25-3.16 (m, 2H, CH_2), 2.55 (dt, *J* = 14.5 and 7 Hz, 1H, CH_2), 2.29 (dt, *J* = 14.5 and 6.5 Hz, 1H, CH_2).

^{13}C-NMR ($CDCl_3$, 125 MHz): δ = 167.6 (C=O), 146.8 (C_{Ar}), 137.5 (C_{Ar}), 136.3 (C_{Ar}), 132.3 (CH_{Ar}), 131.2 (C_{Ar}), 129.6 (CH_{Ar}), 128.5 (2CH_{Ar}), 127.9 (CH_{Ar}), 127.8 (2CH_{Ar}), 127.4 (C_{Ar}), 123.3 (CH_{Ar}), 122.3 (CH_{Ar}), 122.2 (CH_{Ar}), 122.1 (CH_{Ar}), 119.5 (CH_{Ar}), 119.1 (CH_{Ar}), 113.4 (C_{Ar}), 111.3 (CH_{Ar}), 90.3 (C), 73.4 (CH_2), 65.8 (CH_2), 40.0 (CH_2), 36.4 (CH_2), 24.8 (CH_2).

HR-MS (ESI): m/z $[M+Na]^+$ cald for $C_{27}H_{26}N_2O_3Na^+$: 449.1836, found: 449.1833.

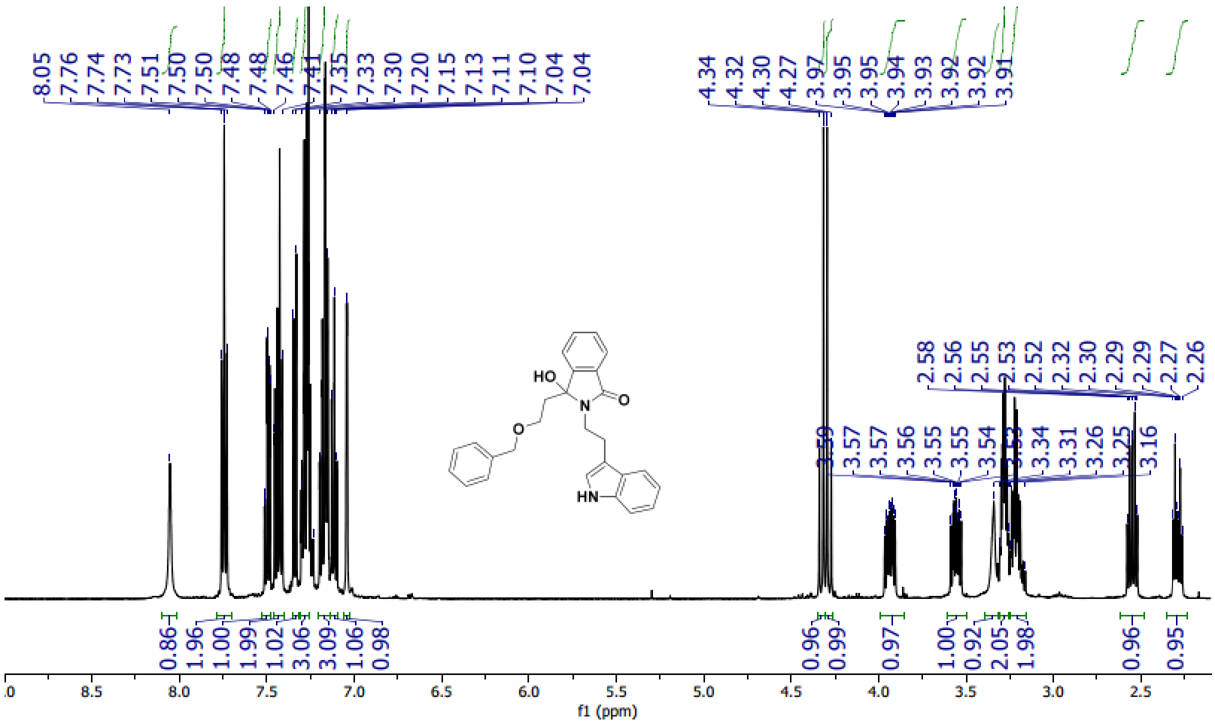
8.05 7.76 7.74 7.73 7.51 7.50 7.50 7.48 7.48 7.46 7.41 7.35 7.33 7.30 7.20 7.15 7.13 7.11 7.10 7.04 7.04
4.34 4.32 4.30 4.27 3.97 3.95 3.95 3.94 3.93 3.92 3.92 3.91
2.58 2.56 2.55 2.53 2.52 2.32 2.30 2.29 2.29 2.27 2.26
3.59 3.57 3.57 3.56 3.55 3.55 3.54 3.53 3.34 3.31 3.26 3.25 3.16
HO
N
O
O
HN
0.86 1.96 1.00 1.99 1.02 3.06 3.09 1.06 0.98
0.96 0.99 0.97 1.00 0.92 2.05 1.98 0.96 0.95
f1 (ppm)

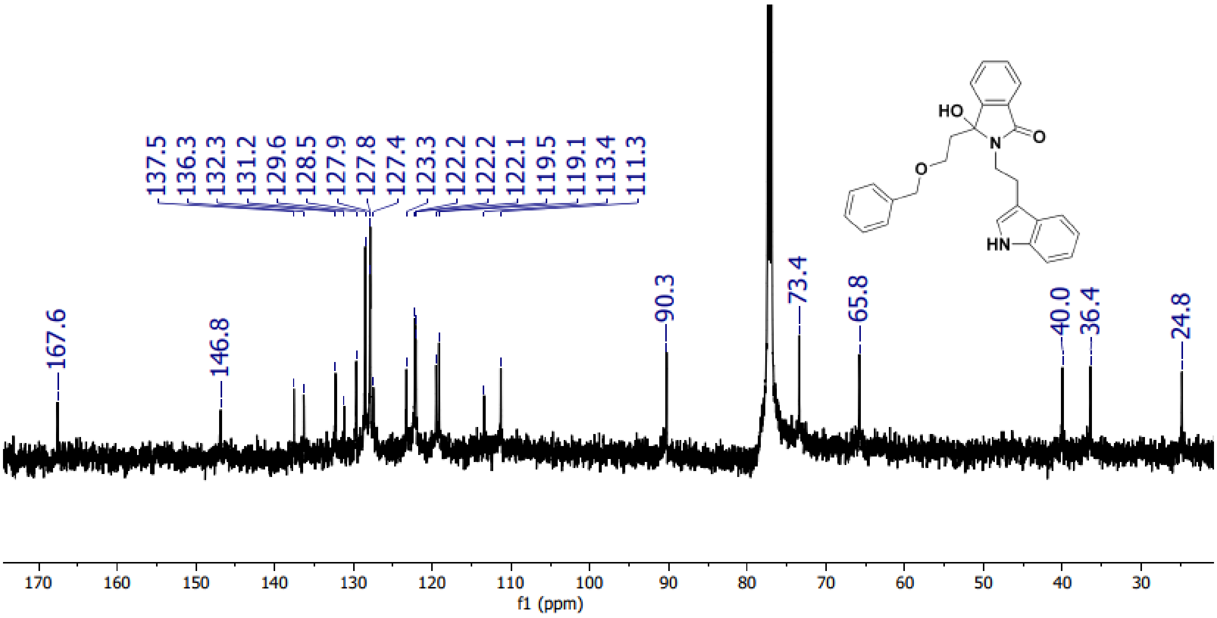
137.5 136.3 132.3 131.2 129.6 128.5 127.9 127.8 127.4 123.3 122.2 122.2 122.1 119.5 119.1 113.4 111.3
167.6
146.8
90.3
73.4
65.8
40.0
36.4
24.8
HO
N
O
O
HN
f1 (ppm)

3-(2-(Benzyloxy)ethyl)-3-hydroxyisoindolin-1-one (1ae)

Following the procedure for the synthesis of 3-hydroxyisoindolin-1-ones **1** using (*Z*)-3-(2-(benzyloxy)ethylidene)isobenzofuran-1(3*H*)-one **2b** (133 mg, 0.5 mmol, 1 equiv.) and ammonium acetate (193 mg, 2.5 mmol, 5 equiv.) with purification using column chromatography (*n*-hexane/ethyl acetate, 40:60), afforded the desired compound **1ae.**

White solid; yield: 90 mg (64%), mp: 139-146 °C.

IR (KBr): 3410, 3048, 2893, 1705, 1420, 1358, 1111, 1026, 702 cm^{-1}.

^{1}H-NMR ($CDCl_3$, 500 MHz): δ = 7.74 (d, *J* = 7.5 Hz, 1H, CH_{Ar}), 7.58 (td, *J* = 7.5 and 1 Hz, 1H, CH_{Ar}), 7.51-7.47 (m, 2H, CH_{Ar}), 7.39-7.36 (m, 2H, CH_{Ar}), 7.34-7.31 (m, 3H, CH_{Ar}), 6.94 (br s, 1H, NH), 4.58 (d, *J* = 12 Hz, 1H, CH_2), 4.55 (d, *J* = 12 Hz, 1H, CH_2), 3.95 (td, *J* = 8.5 and 3.5 Hz, 1H, CH_2), 3.73-3.69 (m, 1H, CH_2), 3.71 (br s, 1H, OH), 2.47-2.42 (m, 1H, CH_2), 2.07 (ddd, *J* = 12.5, 8.5 and 4 Hz, 1H, CH_2).

^{13}C-NMR ($CDCl_3$, 125 MHz): δ = 169.0 (C=O), 149.0 (C_{Ar}), 137.5 (C_{Ar}), 133.0, 130.6 (C_{Ar}), 129.8, 128.8 ($2CH_{Ar}$), 128.2, 128.0 ($2CH_{Ar}$),123.8, 122.1, 87.1, 73.7, 66.2, 38.3.

HR-MS (ESI): m/z $[M+H]^+$ cald for $C_{17}H_{18}NO_3^+$: 284.1281, found: 284.1283.

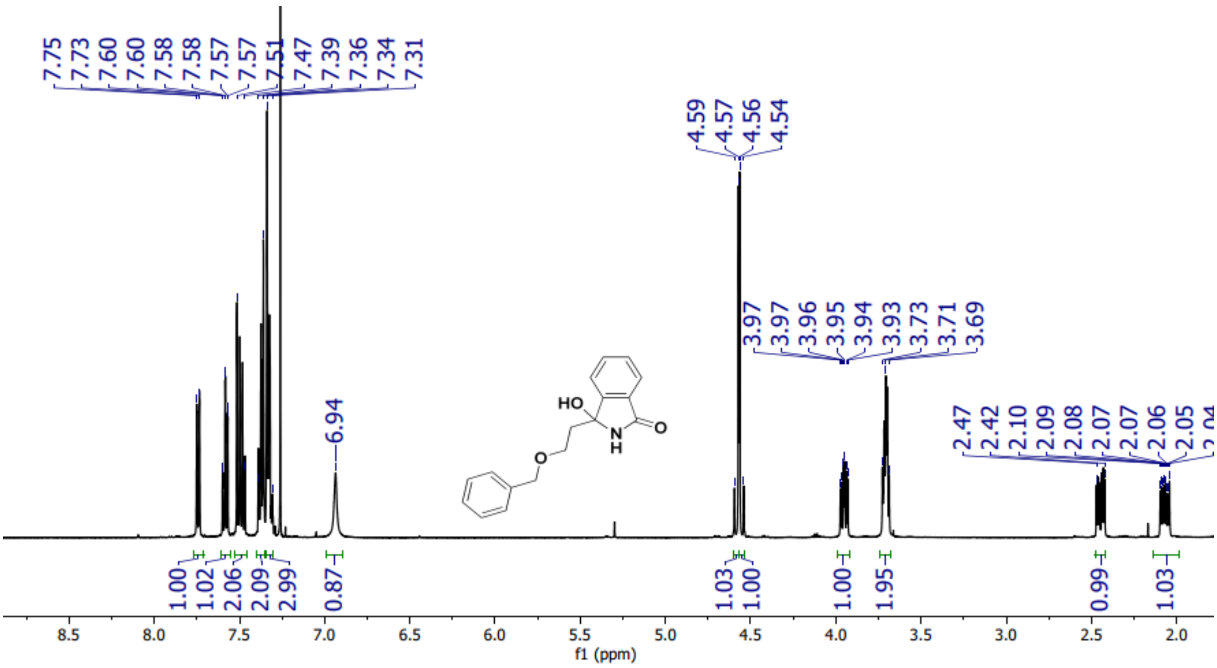
7.75
7.73
7.60
7.60
7.58
7.58
7.57
7.57
7.51
7.47
7.39
7.36
7.34
7.31
6.94
4.59
4.57
4.56
4.54
3.97
3.97
3.96
3.95
3.94
3.93
3.73
3.71
3.69
2.47
2.42
2.10
2.09
2.08
2.07
2.07
2.06
2.05
HO
N
H
O
O
1.00
1.02
2.06
2.09
2.99
0.87
1.03
1.00
1.00
1.95
0.99
1.03
f1 (ppm)

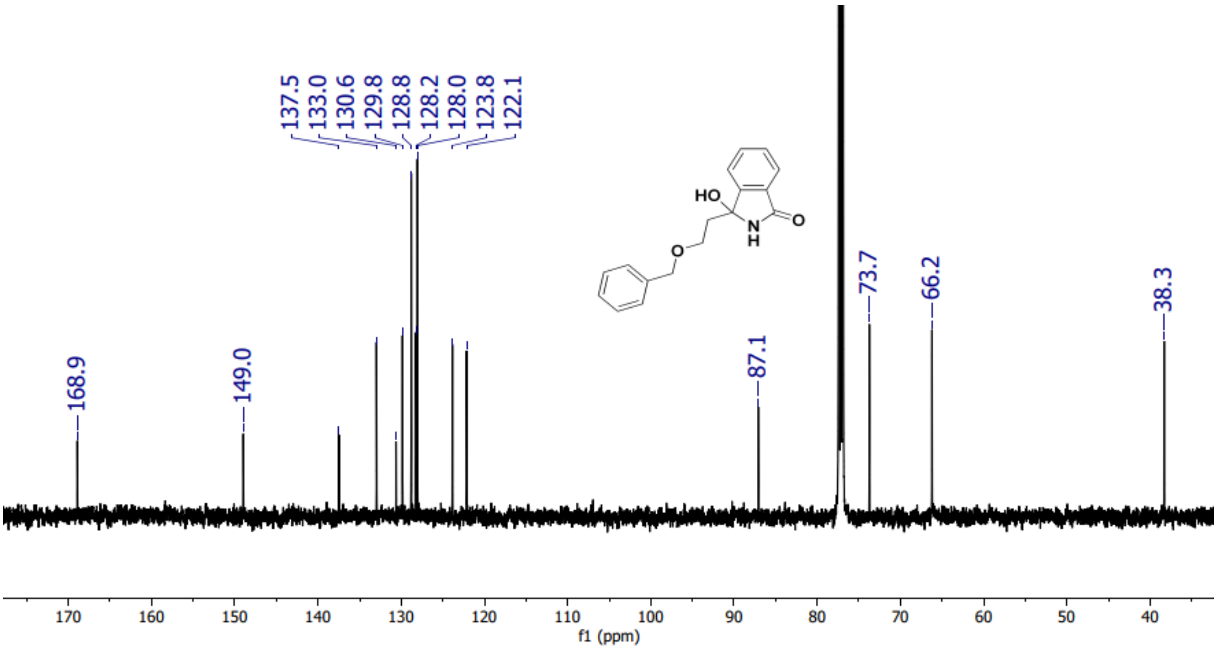
137.5
133.0
130.6
129.8
128.8
128.2
128.0
123.8
122.1
HO
N
H
O
O
168.9
149.0
87.1
73.7
66.2
38.3
f1 (ppm)

Synthesis of 3-Benzyl-2-butylisoindolin-1-one (8a)

*From 3-benzyl-2-butyl-3-hydroxyisoindolin-1-one (**1a**)*

Sodium cyanoborohydride (314 mg, 5 mmol, 10 equiv.) and *p*-toluene sulfonic acid monohydrate (950 mg, 5 mmol, 10 equiv.) were subsequently introduced to an iced-cooled solution of 3-benzyl-2-butyl-3-hydroxyisoindolin-1-one **1a** (148 mg, 0.5 mmol, 1 equiv.) in *iso*-propanol (2 mL). The reaction was conducted at 50 °C under either ultrasonic irradiation or conventional heating. The reaction was quench with the addition of the saturated aqueous solution of $NaHCO_3$, followed with the extraction with dichloromethane (3x5 mL). The combined organic layer was washed with brine, dried over Na_2SO_4, filtered and removed under vacuum. The crude product was purified by column chromatography using the eluent of *n*-hexane/ethyl acetate (9:1).

Ultrasonic irradiation: white solid; yield: 240 mg (86%), mp: 89-91 °C.

Heating process: white solid; 218 mg (78%), mp: 87-92 °C.

*From (*Z*)-3-benzylideneisobenzofuran-1(3H)-one (**2a**)*

(*Z*)-3-Benzylideneisobenzofuran-1(3*H*)-one **2a** (111 mg, 0.5 mmol, 1 equiv.) and *n*-butylamine (0.099 mL, 1 mmol, 2 equiv.) was dissolved in 2 mL of *iso*-propanol. The flask was placed in the pre-heated ultrasonic bath (50 °C) and the reaction was carried out for 30 min. The reaction mixture was placed in the ice bath, followed with the addition of $NaBH_3CN$ (314 mg, 5 mmol, 10 equiv.) and *p*-toluene sulfonic acid monohydrate (950 mg, 5 mmol, 10 equiv.). The reaction was continued under ultrasonic irradiation at 50 °C for 1h. The reaction was quench with the addition of the saturated aqueous solution of $NaHCO_3$, followed with the extraction with dichloromethane (3x5 mL). The combined organic layer was washed with brine, dried over Na_2SO_4, filtered and removed under vacuum. The crude product was purified by column chromatography using the eluent of *n*-hexane/ethyl acetate (9:1).

For scaled up synthesis of **8a**, the same procedure (small scale) of **8a** was conducted by using 2,22 g of (*Z*)-3-benzylideneisobenzofuran-1(3*H*)-one **2a** (10 mmol, 1 equiv.), 1,98 mL of *n*-butylamine (20 mmol, 2 equiv.), $NaBH_3CN$ (6,28 g, 100 mmol, 10 equiv.) and *p*-toluene sulfonic acid monohydrate (19 g, 100 mmol, 10 equiv.)

Small scale: white solid; yield: 121 mg (86%), mp: 88-91 °C.

Scaled up synthesis: white solid; 2.27 g (81%), mp: 88-92 °C.

IR (KBr): 3024, 2970, 2924, 2862, 1681, 1604, 1458, 1419, 1273, 1095, 702 cm^{-1}.

^{1}H-NMR ($CDCl_3$, 500 MHz): δ = 7.78-7.76 (m, 1H, CH_{Ar}), 7.40-7.36 (m, 2H, CH_{Ar}), 7.28-7.22 (m, 3H, CH_{Ar}), 7.09-7.07 (m, 2H, CH_{Ar}), 6.92-6.90 (m, 1H, CH_{Ar}), 4.80, (dd, *J* = 8 and 4.5 Hz, 1H, CH), 4.13-4.07 (m, 1H, CH_2), 3.40 (dd, *J* = 14 and 4.5 Hz, 1H, CH_2), 3.22 (ddd, *J* = 14, 8.5, 5.5 Hz, 1H, CH_2), 2.84 (dd, *J* = 14 and 8 Hz, 1H, CH_2), 1.71-1.59 (m, 2H, CH_2), 1.40-1.32 (m, 2H, CH_2), 0.96 (t, *J* = 7.5 Hz, 3H, CH_3).

^{13}C-NMR ($CDCl_3$, 125 MHz): δ = 168.3 (C=O), 144.9 (C_{Ar}), 136.1 (C_{Ar}), 132.5 (C_{Ar}), 130.8 (CH_{Ar}), 129.6 (2CH_{Ar}), 128.6 (2CH_{Ar}), 128.2 (CH_{Ar}), 127.1 (CH_{Ar}), 123.6 (CH_{Ar}), 123.0 (CH_{Ar}), 60.0 (CH_{Ar}), 40.0 (CH_2), 38.5 (CH_2), 30.5 (CH_2), 20.2 (CH_2), 13.9 (CH_3).

HR-MS (ESI): m/z $[M+Na]^+$ cald for $C_{19}H_{21}NONa^+$: 302.1515, found: 302.1517.

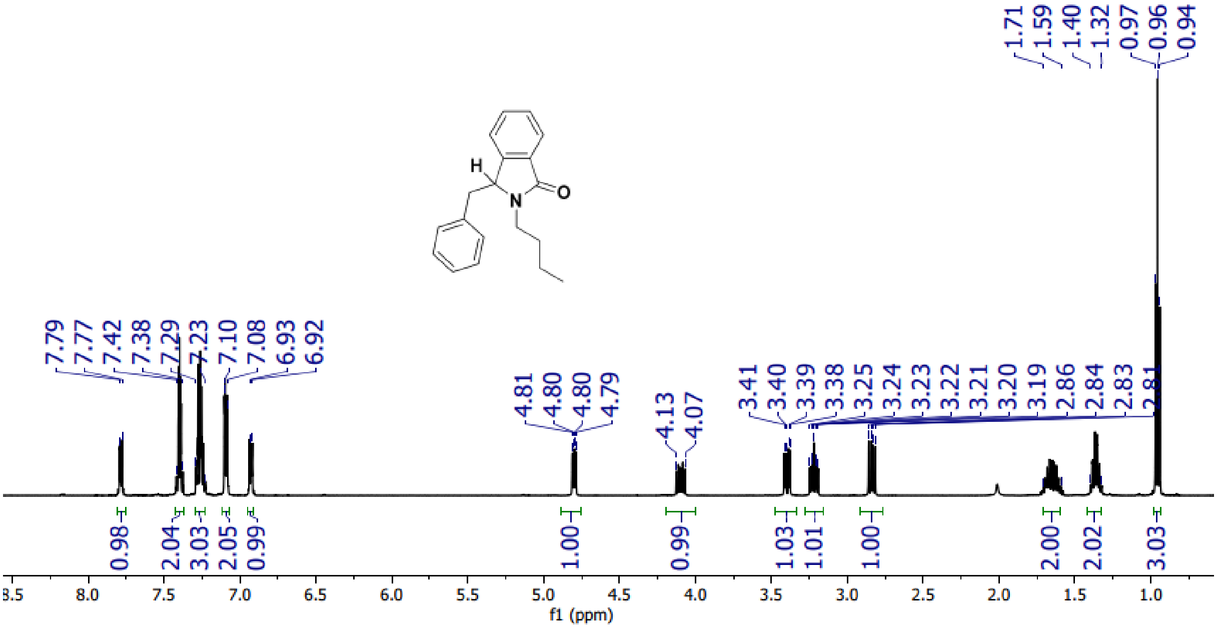
7.79
7.77
7.42
7.38
7.29
7.23
7.10
7.08
6.93
6.92
4.81
4.80
4.80
4.79
4.13
4.07
3.41
3.40
3.39
3.38
3.25
3.24
3.23
3.22
3.21
3.20
3.19
2.86
2.84
2.83
2.81
1.71
1.59
1.40
1.32
0.97
0.96
0.94
0.98
2.04
3.03
2.05
0.99
1.00
0.99
1.03
1.01
1.00
2.00
2.02
3.03
f1 (ppm)

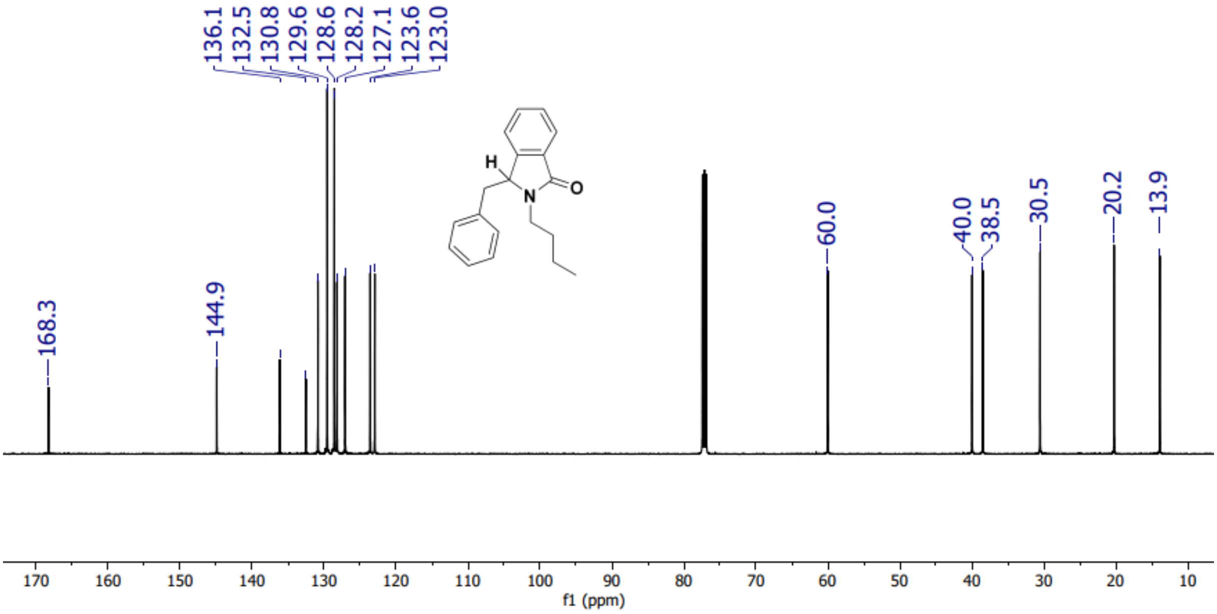
136.1
132.5
130.8
129.6
128.6
128.2
127.1
123.6
123.0
168.3
144.9
60.0
40.0
38.5
30.5
20.2
13.9
f1 (ppm)

Synthesis of (Z)-3-Benzylideneisoindolin-1-one (9u)

*From 3-benzyl-3-hydroxyisoindolin-1-one (**1u**)*

To the cooled solution of 3-benzyl-3-hydroxyisoindolin-1-one **1u** (119 mg, 0.5 mmol, 1 equiv.) in *iso*-propanol (2 mL), the aqueous solution of HCl (6M, 1.67 mL, 10 mmol, 20 equiv.). The reaction was conducted at 50 °C under either ultrasonic irradiation or conventional heating. The reaction was quenched with ethyl acetate (10 mL) and distilled water (10 mL). The aqueous phase was extracted with ethyl acetate (3x10 mL). The combined organic phase was washed with brine, dried over Na_2SO_4, and evaporated to dryness under reduced pressure. The desired product **9u** was isolated by column chromatography using the mixture of *n*-hexane/ethyl acetate (7:3).

Ultrasonic irradiation: light yellow solid; yield: 93 mg (85%), mp: 180-183 °C.

Heating process: light yellow solid; 85 mg (77%), mp: 183-187 °C.

From (Z)*-3-benzylideneisobenzofuran-1(3H)-one (**2a**)*

In 25 mL of round-bottomed-flask was added (*Z*)-3-benzylideneisobenzofuran-1(3*H*)-one **2a** (111 mg, 0.5 mmol, 1 equiv), ammonium acetate (193 mg, 2.5 mmol, 5 equiv.) and *iso*-propanol (2 mL). The mixture was reacted under ultrasonic irradiation at 50 °C for 30 min. The flask was then placed in the ice bath and the aqueous solution of HCl (6M, 1.67 mL, 10 mmol, 20 equiv.) was then subjected to the mixture. The reaction was continued under ultrasonic irradiation at 50 °C for 1h. After the consumption of the intermediate **1u** (based on TLC analysis), ethyl acetate (10 mL) and distilled water (10 mL) were added to the cooled reaction mixture. The aqueous phase was extracted with ethyl acetate (3x10 mL). The combined organic phase was washed with brine, dried over Na_2SO_4, and evaporated to dryness under reduced pressure. The desired product **9u** was isolated by column chromatography using the mixture of *n*-hexane/ethyl acetate (7:3).

For scaled up synthesis of **9u**, the same procedure (small scale) of **7u** was conducted by using 2,22 g of (*Z*)-3-benzylideneisobenzofuran-1(3*H*)-one **2a** (10 mmol, 1 equiv.), 3.86 g of ammonium acetate (50 mmol, 5 equiv.), and 34 mL of HCl (6M, 200 mmol, 20 equiv.).

Small scale: yellow solid; yield: 77 mg (70%), mp: 180-185 °C.

Scaled up synthesis: white solid; 1,42 g (64%), mp: 181-187 °C.

IR (KBr): 3264, 3055, 1697, 1612, 1458, 1312, 659 cm^{-1}.

^{1}H-NMR ($CDCl_3$, 500 MHz): δ = 8.55 (br s, 1H, NH), 7.88 (d, *J* = 7.5 Hz, 1H, CH_{Ar}), 7.78 (d, *J* = 7.5 Hz, 1H, CH_{Ar}), 7.63 (t, *J* = 7.5 Hz, 1H, CH_{Ar}), 7.52 (t, *J* = 7.5 Hz, 1H, CH_{Ar}), 7.49-7.42 (m, 4H, CH_{Ar}), 7.31 (t, *J* = 7.5 Hz, 1H, CH_{Ar}), 6.56 (s, 1H, -CH=).

^{13}C-NMR ($CDCl_3$, 125 MHz): δ = 169.3 (C=O), 138.4 (C_{Ar}), 135.1 (C_{Ar}), 133.2 (C_{Ar}), 132.4 (CH_{Ar}), 129.3 (2CH_{Ar}), 129.2 (CH_{Ar}), 128.8 (C_{Ar}), 128.7 (2CH_{Ar}), 127.8 (CH_{Ar}), 123.7 (CH_{Ar}), 119.9 (CH_{Ar}), 106.2 (-CH=).

HR-MS (ESI): m/z $[M+H]^+$ cald for $C_{15}H_{12}NO^+$: 222.0913, found: 222.0914.

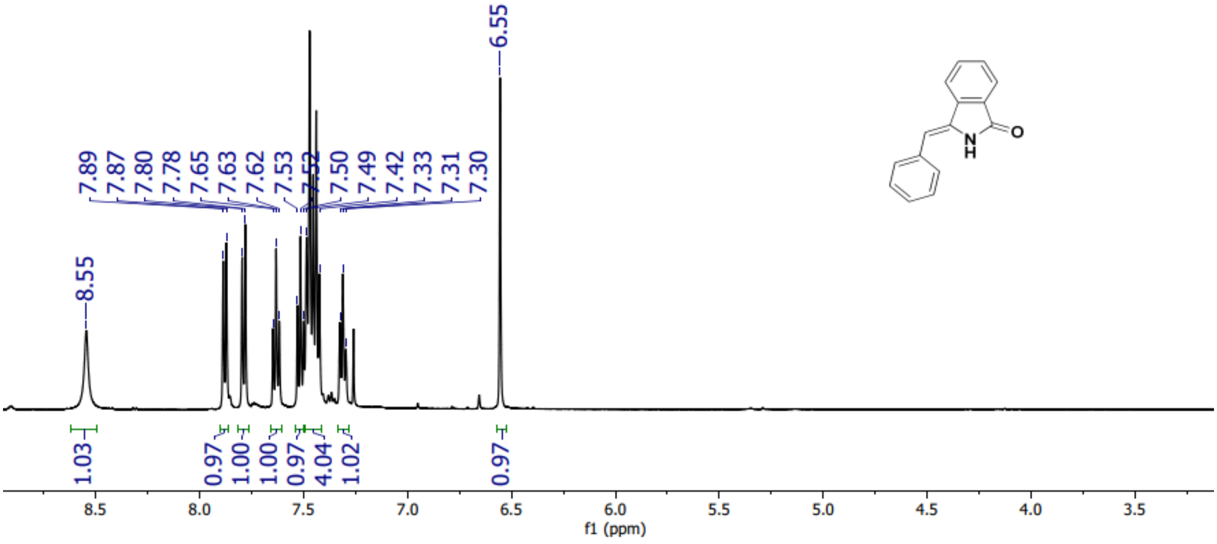
8.55
7.89
7.87
7.80
7.78
7.65
7.63
7.62
7.53
7.52
7.50
7.49
7.42
7.33
7.31
7.30
6.55
1.03
0.97
1.00
1.00
0.97
4.04
1.02
0.97
8.5
8.0
7.5
7.0
6.5
6.0
5.5
5.0
4.5
4.0
3.5
f1 (ppm)
N
H
O

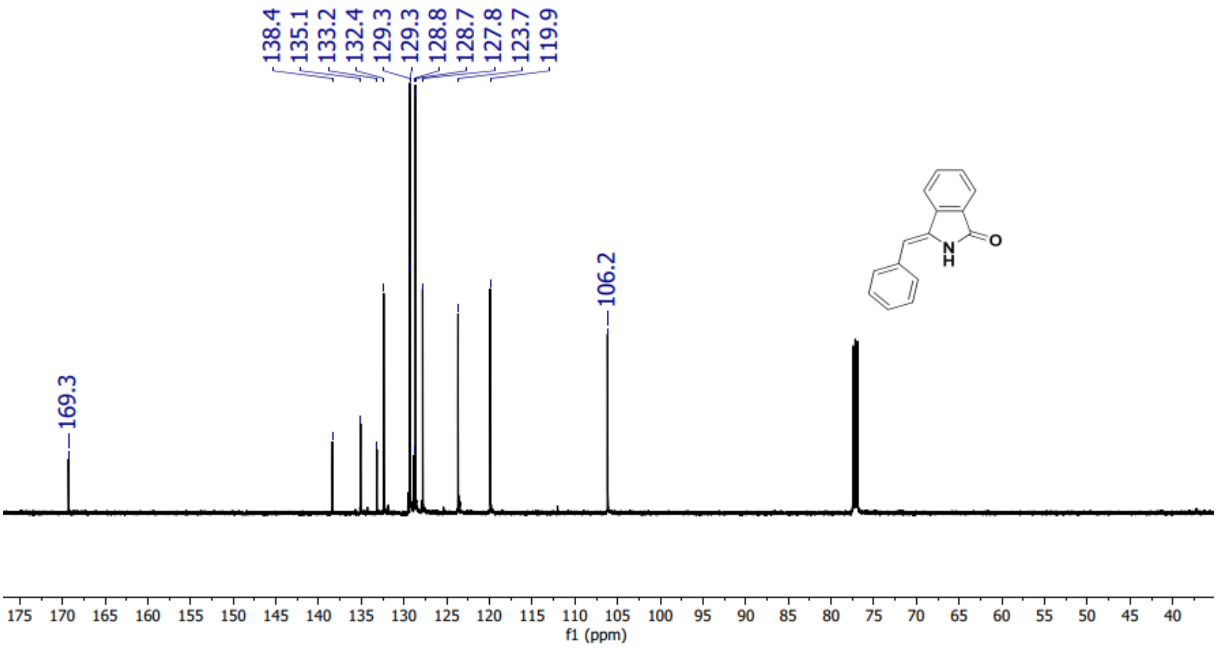
138.4
135.1
133.2
132.4
129.3
129.3
128.8
128.7
127.8
123.7
119.9
169.3
106.2
175
170
165
160
155
150
145
140
135
130
125
120
115
110
105
100
95
90
85
80
75
70
65
60
55
50
45
40
f1 (ppm)
N
H
O
